# Supplementary material for: Quantitative uniqueness of human brain evolution revealed through phylogenetic comparative analysis
Source: eLife. 2019 Jan 31;8:e41250. doi: 10.7554/eLife.41250 (PMC6379089; doi:10.7554/eLife.41250)
Supplement: Source data 2. — Contains the following files: 1. data set 1.csv 2. data set 2.csv 3. data set 3.csv 4. consensus.tree.txt 5. tree.block.txt 6. grafted.tree.txt [file elife-41250-data2.zip › results details/bayou results details.html]

 

 

 

 
 
 


 Bayou Results 

 
 
 
 
 
 
 
 
 
 
 
 

 
 
 


 


 

 

 


 

 
 

 


 

 

 
 
 
 
 
 

 


 


 Bayou Results 

 


 
 ECV ~ Body Mass 
 
 OU Un-weighted Predictor 
 
 Convergence 
   
 Plots show Gelman’s R statistics for log likelihood,  \(\alpha\) , and  \(\sigma^2\) . Solid lines indicate values Gelman’s R, and dashed lines indicate 95% CIs. 
 
 
 Likelihood 
   
 Plot shows the posterior probabilities of a shift occuring on each branch for each chain prior to merging. 
 
 
 Summary Statistics 
 
 chain1 
 
 
  
 Mean 
 SD 
 Naive SE 
 Time-series SE 
 Effective Size 
 HPD95Lower 
 HPD95Upper 
 
 
 
 
 lnL 
 238.1995802 
 122.4300308 
 0.1730555 
 1.2243573 
 9999.06838 
 220.7339537 
 257.0483448 
 
 
 prior 
 -79.4250054 
 9.4717222 
 0.0133883 
 0.4989513 
 360.36414 
 -97.5722573 
 -62.7195212 
 
 
 alpha 
 4.4671710 
 7.3078805 
 0.0103297 
 0.8032901 
 82.76335 
 0.0600634 
 18.1867214 
 
 
 sigma^2 
 0.0225446 
 0.0407490 
 0.0000576 
 0.0044556 
 83.64089 
 0.0005501 
 0.0840875 
 
 
 shift number 
 12.5040909 
 2.0410771 
 0.0028851 
 0.0924041 
 487.90626 
 9.0000000 
 16.0000000 
 
 
 N theta 
 13.5040909 
 2.0410771 
 0.0028851 
 0.0924041 
 487.90626 
 10.0000000 
 17.0000000 
 
 
 root theta 
 -0.8949691 
 0.2577805 
 0.0003644 
 0.0170615 
 228.27908 
 -1.2466628 
 -0.5250178 
 
 
 root beta 
 0.6871632 
 0.1181884 
 0.0001671 
 0.0066677 
 314.19731 
 0.5758711 
 0.8439854 
 
 
 all theta 
 -0.9918978 
 0.5895005 
 NA 
 NA 
 NA 
 NA 
 NA 
 
 
 all beta 
 0.7673352 
 0.2330846 
 NA 
 NA 
 NA 
 NA 
 NA 
 
 
 
 
 chain2 
 
 
  
 Mean 
 SD 
 Naive SE 
 Time-series SE 
 Effective Size 
 HPD95Lower 
 HPD95Upper 
 
 
 
 
 lnL 
 238.1166512 
 15.9358457 
 0.0225254 
 0.8746991 
 331.91957 
 224.3365057 
 253.4166558 
 
 
 prior 
 -79.6167181 
 8.7921682 
 0.0124278 
 0.4375569 
 403.75960 
 -97.4821998 
 -64.9896912 
 
 
 alpha 
 3.5321504 
 4.4306651 
 0.0062628 
 0.7400471 
 35.84422 
 0.3916937 
 10.9270386 
 
 
 sigma^2 
 0.0191945 
 0.0298866 
 0.0000422 
 0.0046974 
 40.48041 
 0.0022675 
 0.0530316 
 
 
 shift number 
 12.6600546 
 2.1089068 
 0.0029810 
 0.1036098 
 414.29824 
 10.0000000 
 17.0000000 
 
 
 N theta 
 13.6600546 
 2.1089068 
 0.0029810 
 0.1036098 
 414.29824 
 11.0000000 
 18.0000000 
 
 
 root theta 
 -0.7689019 
 0.2105803 
 0.0002977 
 0.0131899 
 254.89019 
 -0.9939623 
 -0.4361902 
 
 
 root beta 
 0.6729566 
 0.0976750 
 0.0001381 
 0.0044102 
 490.50384 
 0.5837124 
 0.7352264 
 
 
 all theta 
 -1.0875801 
 0.6176098 
 NA 
 NA 
 NA 
 NA 
 NA 
 
 
 all beta 
 0.7952158 
 0.2251197 
 NA 
 NA 
 NA 
 NA 
 NA 
 
 
 
 
 combined chains 
 
 
  
 Mean 
 SD 
 Naive SE 
 Time-series SE 
 Effective Size 
 HPD95Lower 
 HPD95Upper 
 
 
 
 
 lnL 
 239.4526688 
 6.4815984 
 0.0077431 
 0.2247830 
 831.45240 
 225.6856029 
 251.7243879 
 
 
 prior 
 -78.4965090 
 7.6618899 
 0.0091531 
 0.2985010 
 658.84054 
 -95.1556779 
 -66.0802773 
 
 
 alpha 
 3.1987946 
 4.6981872 
 0.0056126 
 0.4964562 
 89.55685 
 0.0651811 
 10.9043902 
 
 
 sigma^2 
 0.0156321 
 0.0223119 
 0.0000267 
 0.0026725 
 69.70143 
 0.0006273 
 0.0512932 
 
 
 shift number 
 12.4179982 
 1.7824208 
 0.0021293 
 0.0665229 
 717.92321 
 10.0000000 
 16.0000000 
 
 
 N theta 
 13.4179982 
 1.7824208 
 0.0021293 
 0.0665229 
 717.92321 
 11.0000000 
 17.0000000 
 
 
 root theta 
 -0.7758925 
 0.2306230 
 0.0002755 
 0.0123306 
 349.81145 
 -0.9966859 
 -0.4650088 
 
 
 root beta 
 0.6621634 
 0.0898212 
 0.0001073 
 0.0038586 
 541.86806 
 0.5804227 
 0.7233932 
 
 
 all theta 
 -1.0311697 
 0.5883117 
 NA 
 NA 
 NA 
 NA 
 NA 
 
 
 all beta 
 0.7759491 
 0.2254518 
 NA 
 NA 
 NA 
 NA 
 NA 
 
 
 
 
 
 Shifts 
      
 A shows the locations of the selection regimes on the phylogeny. B shows the optimum regression lines associated with each regime. C and D show the posterior distribution of  \(\beta\)  and  \(\theta\)  values associated with each regime. 
   
 Plot shows the median values for parameters associated with each selection regime, along with the posterior probability associeated with each regime.  \(\theta\)  is the intercept of the optimum regression line, and  \(\beta\)  is the coefficient of the predictor. 
 
 
 OU parameters 
 
 
 
  
 chain1 
 chain2 
 combined chains 
 
 
 
 
 alpha 
 4.4671710 
 3.5321504 
 3.1987946 
 
 
 sigma squared 
 0.0225446 
 0.0191945 
 0.0156321 
 
 
 stationary variance 
 0.0025234 
 0.0027171 
 0.0024434 
 
 
 phylogenetic halflife 
 0.1551647 
 0.1962394 
 0.2166901 
 
 
 phylogenetic half life in units of tree height 
 0.0021255 
 0.0026881 
 0.0029682 
 
 
 
 
 
 
 OU Weighted Predictor 
 
 Convergence 
   
 Plots show Gelman’s R statistics for log likelihood,  \(\alpha\) , and  \(\sigma^2\) . Solid lines indicate values Gelman’s R, and dashed lines indicate 95% CIs. 
 
 
 Likelihood 
   
 Plot shows the posterior probabilities of a shift occuring on each branch for each chain prior to merging. 
 
 
 Summary Statistics 
 
 chain1 
 
 
  
 Mean 
 SD 
 Naive SE 
 Time-series SE 
 Effective Size 
 HPD95Lower 
 HPD95Upper 
 
 
 
 
 lnL 
 237.9218364 
 19.7416313 
 0.0279049 
 0.6617010 
 890.107694 
 221.8920862 
 256.0552664 
 
 
 prior 
 -80.3339274 
 8.9460986 
 0.0126454 
 0.4374058 
 418.309945 
 -98.3921206 
 -64.9055705 
 
 
 alpha 
 5.0679840 
 5.1304656 
 0.0072519 
 0.7196491 
 50.824373 
 0.1436803 
 15.4605445 
 
 
 sigma^2 
 0.0255562 
 0.0251606 
 0.0000356 
 0.0079802 
 9.940587 
 0.0010899 
 0.0777228 
 
 
 shift number 
 12.7274411 
 2.0543322 
 0.0029038 
 0.0914053 
 505.124724 
 9.0000000 
 16.0000000 
 
 
 N theta 
 13.7274411 
 2.0543322 
 0.0029038 
 0.0914053 
 505.124724 
 10.0000000 
 17.0000000 
 
 
 root theta 
 -0.8862630 
 0.2704600 
 0.0003823 
 0.0232761 
 135.016424 
 -1.2755803 
 -0.5218193 
 
 
 root beta 
 0.6788669 
 0.1086343 
 0.0001536 
 0.0079276 
 187.780011 
 0.5763822 
 0.8559412 
 
 
 all theta 
 -1.0569796 
 0.5850637 
 NA 
 NA 
 NA 
 NA 
 NA 
 
 
 all beta 
 0.7812911 
 0.2295259 
 NA 
 NA 
 NA 
 NA 
 NA 
 
 
 
 
 chain2 
 
 
  
 Mean 
 SD 
 Naive SE 
 Time-series SE 
 Effective Size 
 HPD95Lower 
 HPD95Upper 
 
 
 
 
 lnL 
 223.2348819 
 20.1483948 
 0.0284799 
 1.2015290 
 281.19810 
 192.7599191 
 252.6241780 
 
 
 prior 
 -107.7001588 
 17.4343657 
 0.0246436 
 0.6687513 
 679.64634 
 -143.4763521 
 -74.9549714 
 
 
 alpha 
 0.0541054 
 0.0487127 
 0.0000689 
 0.0070444 
 47.81810 
 0.0109892 
 0.1592881 
 
 
 sigma^2 
 0.0006807 
 0.0012414 
 0.0000018 
 0.0000449 
 763.39316 
 0.0003380 
 0.0010336 
 
 
 shift number 
 18.6463564 
 3.8560876 
 0.0054506 
 0.1349536 
 816.44099 
 11.0000000 
 26.0000000 
 
 
 N theta 
 19.6463564 
 3.8560876 
 0.0054506 
 0.1349536 
 816.44099 
 12.0000000 
 27.0000000 
 
 
 root theta 
 -0.8922021 
 0.1337559 
 0.0001891 
 0.0112901 
 140.35559 
 -1.1360875 
 -0.6469820 
 
 
 root beta 
 0.6920913 
 0.0547437 
 0.0000774 
 0.0056320 
 94.47927 
 0.6027534 
 0.7966366 
 
 
 all theta 
 -1.1677733 
 0.8743207 
 NA 
 NA 
 NA 
 NA 
 NA 
 
 
 all beta 
 0.8300406 
 0.4082974 
 NA 
 NA 
 NA 
 NA 
 NA 
 
 
 
 
 combined chains 
 
 
  
 Mean 
 SD 
 Naive SE 
 Time-series SE 
 Effective Size 
 HPD95Lower 
 HPD95Upper 
 
 
 
 
 lnL 
 228.4745348 
 14.6627923 
 0.0175166 
 1.0690629 
 188.11647 
 196.7826223 
 252.0807142 
 
 
 prior 
 -95.2882451 
 21.0600772 
 0.0251590 
 1.0741921 
 384.37578 
 -134.2203000 
 -62.2455525 
 
 
 alpha 
 1.5826884 
 2.9676490 
 0.0035452 
 0.4610920 
 41.42380 
 0.0089454 
 8.4162423 
 
 
 sigma^2 
 0.0081899 
 0.0144365 
 0.0000172 
 0.0057199 
 6.37012 
 0.0002748 
 0.0441031 
 
 
 shift number 
 15.9444473 
 4.5471523 
 0.0054322 
 0.2061424 
 486.56884 
 8.0000000 
 24.0000000 
 
 
 N theta 
 16.9444473 
 4.5471523 
 0.0054322 
 0.2061424 
 486.56884 
 9.0000000 
 25.0000000 
 
 
 root theta 
 -0.8391474 
 0.1453729 
 0.0001737 
 0.0108936 
 178.08515 
 -1.0759083 
 -0.5340917 
 
 
 root beta 
 0.6618703 
 0.0521507 
 0.0000623 
 0.0041416 
 158.55613 
 0.5731643 
 0.7628291 
 
 
 all theta 
 -1.1224389 
 0.7885545 
 NA 
 NA 
 NA 
 NA 
 NA 
 
 
 all beta 
 0.8170445 
 0.3651531 
 NA 
 NA 
 NA 
 NA 
 NA 
 
 
 
 
 
 Shifts 
      
 A shows the locations of the selection regimes on the phylogeny. B shows the optimum regression lines associated with each regime. C and D show the posterior distribution of  \(\beta\)  and  \(\theta\)  values associated with each regime. 
   
 Plot shows the median values for parameters associated with each selection regime, along with the posterior probability associeated with each regime.  \(\theta\)  is the intercept of the optimum regression line, and  \(\beta\)  is the coefficient of the predictor. 
 
 
 
 OU parameters 
 
 
 
  
 chain1 
 chain2 
 combined chains 
 
 
 
 
 alpha 
 5.0679840 
 0.0541054 
 1.5826884 
 
 
 sigma squared 
 0.0255562 
 0.0006807 
 0.0081899 
 
 
 stationary variance 
 0.0025213 
 0.0062907 
 0.0025873 
 
 
 phylogenetic halflife 
 0.1367698 
 12.8110419 
 0.4379556 
 
 
 phylogenetic half life in units of tree height 
 0.0018735 
 0.1754862 
 0.0059991 
 
 
 
 
 
 BM 
 
 Convergence 
   
 Plots show Gelman’s R statistics for log likelihood,  \(\alpha\) , and  \(\sigma^2\) . Solid lines indicate values Gelman’s R, and dashed lines indicate 95% CIs. 
 
 
 Likelihood 
   
 Plot shows the posterior probabilities of a shift occuring on each branch for each chain prior to merging. 
 
 
 Summary Statistics 
 
 chain1 
 
 
  
 Mean 
 SD 
 Naive SE 
 Time-series SE 
 Effective Size 
 HPD95Lower 
 HPD95Upper 
 
 
 
 
 lnL 
 162.8501112 
 6.8640609 
 0.0097024 
 0.1626607 
 1780.7250 
 160.4734105 
 164.5401197 
 
 
 prior 
 0.2700209 
 0.1429087 
 0.0002020 
 0.0042257 
 1143.7290 
 -0.0015802 
 0.5213542 
 
 
 alpha 
 0.0000010 
 0.0000000 
 0.0000000 
 0.0000000 
 0.0000 
 0.0000010 
 0.0000010 
 
 
 sigma^2 
 0.0009615 
 0.0108924 
 0.0000154 
 0.0001380 
 6226.1991 
 0.0006387 
 0.0010156 
 
 
 shift number 
 0.0000000 
 0.0000000 
 0.0000000 
 0.0000000 
 0.0000 
 0.0000000 
 0.0000000 
 
 
 N theta 
 1.0000000 
 0.0000000 
 0.0000000 
 0.0000000 
 0.0000 
 1.0000000 
 1.0000000 
 
 
 root theta 
 -0.5479505 
 0.1441275 
 0.0002037 
 0.0047487 
 921.1743 
 -0.8325343 
 -0.2674380 
 
 
 root beta 
 0.5903931 
 0.0344444 
 0.0000487 
 0.0007467 
 2127.6723 
 0.5248260 
 0.6564211 
 
 
 all theta 
 -0.5479505 
 0.1441275 
 NA 
 NA 
 NA 
 NA 
 NA 
 
 
 all beta 
 0.5903931 
 0.0344444 
 NA 
 NA 
 NA 
 NA 
 NA 
 
 
 
 
 chain2 
 
 
  
 Mean 
 SD 
 Naive SE 
 Time-series SE 
 Effective Size 
 HPD95Lower 
 HPD95Upper 
 
 
 
 
 lnL 
 162.8178485 
 7.1347227 
 0.0100850 
 0.1754235 
 1654.1646 
 160.4389891 
 164.5406391 
 
 
 prior 
 0.2719569 
 0.1444983 
 0.0002042 
 0.0043446 
 1106.2020 
 0.0017662 
 0.5256841 
 
 
 alpha 
 0.0000010 
 0.0000000 
 0.0000000 
 0.0000000 
 0.0000 
 0.0000010 
 0.0000010 
 
 
 sigma^2 
 0.0009771 
 0.0117742 
 0.0000166 
 0.0001598 
 5427.6002 
 0.0006428 
 0.0010208 
 
 
 shift number 
 0.0000000 
 0.0000000 
 0.0000000 
 0.0000000 
 0.0000 
 0.0000000 
 0.0000000 
 
 
 N theta 
 1.0000000 
 0.0000000 
 0.0000000 
 0.0000000 
 0.0000 
 1.0000000 
 1.0000000 
 
 
 root theta 
 -0.5512008 
 0.1472396 
 0.0002081 
 0.0050064 
 864.9767 
 -0.8293682 
 -0.2657456 
 
 
 root beta 
 0.5904883 
 0.0349657 
 0.0000494 
 0.0007775 
 2022.5881 
 0.5236656 
 0.6566052 
 
 
 all theta 
 -0.5512008 
 0.1472396 
 NA 
 NA 
 NA 
 NA 
 NA 
 
 
 all beta 
 0.5904883 
 0.0349657 
 NA 
 NA 
 NA 
 NA 
 NA 
 
 
 
 
 combined chains 
 
 
  
 Mean 
 SD 
 Naive SE 
 Time-series SE 
 Effective Size 
 HPD95Lower 
 HPD95Upper 
 
 
 
 
 lnL 
 162.9886803 
 1.2564426 
 0.0015010 
 0.0129584 
 9401.136 
 160.5252452 
 164.5397193 
 
 
 prior 
 0.2705965 
 0.1359536 
 0.0001624 
 0.0038109 
 1272.723 
 -0.0012333 
 0.5194633 
 
 
 alpha 
 0.0000010 
 0.0000000 
 0.0000000 
 0.0000000 
 0.000 
 0.0000010 
 0.0000010 
 
 
 sigma^2 
 0.0008240 
 0.0000970 
 0.0000001 
 0.0000005 
 33103.019 
 0.0006410 
 0.0010168 
 
 
 shift number 
 0.0000000 
 0.0000000 
 0.0000000 
 0.0000000 
 0.000 
 0.0000000 
 0.0000000 
 
 
 N theta 
 1.0000000 
 0.0000000 
 0.0000000 
 0.0000000 
 0.000 
 1.0000000 
 1.0000000 
 
 
 root theta 
 -0.5480353 
 0.1429355 
 0.0001708 
 0.0039851 
 1286.453 
 -0.8246761 
 -0.2654532 
 
 
 root beta 
 0.5899000 
 0.0333908 
 0.0000399 
 0.0005885 
 3219.464 
 0.5248356 
 0.6557577 
 
 
 all theta 
 -0.5480353 
 0.1429355 
 NA 
 NA 
 NA 
 NA 
 NA 
 
 
 all beta 
 0.5899000 
 0.0333908 
 NA 
 NA 
 NA 
 NA 
 NA 
 
 
 
 
 
 Shifts 
No shifts were detected with a posterior probability above the cutoff.
 
 OU parameters 
 
 
 
  
 chain1 
 chain2 
 combined chains 
 
 
 
 
 alpha 
 1.000000e-06 
 1.000000e-06 
 1.000000e-06 
 
 
 sigma squared 
 9.615000e-04 
 9.771000e-04 
 8.240000e-04 
 
 
 stationary variance 
 4.807501e+02 
 4.885398e+02 
 4.119915e+02 
 
 
 phylogenetic halflife 
 6.931472e+05 
 6.931472e+05 
 6.931472e+05 
 
 
 phylogenetic half life in units of tree height 
 9.494761e+03 
 9.494761e+03 
 9.494761e+03 
 
 
 
 
 
 Model Selection 
 
 Bayes Factors 
 
 
  
 OU un-weighted predictor 
 OU weighted predictor 
 BM 
 
 
 
 
 OU un-weighted predictor 
 0.00000 
 14.65512 
 37.51062 
 
 
 OU weighted predictor 
 -14.65512 
 0.00000 
 22.85550 
 
 
 BM 
 -37.51062 
 -22.85550 
 0.00000 
 
 
 
 
 
 
 ECV ~ Body Mass (alternate hominin phylogeny) 
 
 OU Un-weighted Predictor 
 
 Convergence 
   
 Plots show Gelman’s R statistics for log likelihood,  \(\alpha\) , and  \(\sigma^2\) . Solid lines indicate values Gelman’s R, and dashed lines indicate 95% CIs. 
 
 
 Likelihood 
   
 Plot shows the posterior probabilities of a shift occuring on each branch for each chain prior to merging. 
 
 
 Summary Statistics 
 
 chain1 
 
 
  
 Mean 
 SD 
 Naive SE 
 Time-series SE 
 Effective Size 
 HPD95Lower 
 HPD95Upper 
 
 
 
 
 lnL 
 241.0540198 
 20.0391281 
 0.0283254 
 0.6023059 
 1106.93834 
 227.3495954 
 256.1030043 
 
 
 prior 
 -82.1802895 
 8.1332266 
 0.0114964 
 0.3561428 
 521.52784 
 -97.6786430 
 -69.0064584 
 
 
 alpha 
 10.2791710 
 11.6108181 
 0.0164120 
 1.4006483 
 68.71752 
 0.3844910 
 30.9124409 
 
 
 sigma^2 
 0.0504784 
 0.0559363 
 0.0000791 
 0.0109034 
 26.31837 
 0.0024549 
 0.1512941 
 
 
 shift number 
 12.7584520 
 1.9637988 
 0.0027758 
 0.0848774 
 535.31579 
 10.0000000 
 16.0000000 
 
 
 N theta 
 13.7584520 
 1.9637988 
 0.0027758 
 0.0848774 
 535.31579 
 11.0000000 
 17.0000000 
 
 
 root theta 
 -0.8688541 
 0.2190940 
 0.0003097 
 0.0140792 
 242.15982 
 -1.2569312 
 -0.5352781 
 
 
 root beta 
 0.6947123 
 0.0990825 
 0.0001401 
 0.0051270 
 373.48405 
 0.5764297 
 0.8562659 
 
 
 all theta 
 -0.9728190 
 0.5859950 
 NA 
 NA 
 NA 
 NA 
 NA 
 
 
 all beta 
 0.7697762 
 0.2195265 
 NA 
 NA 
 NA 
 NA 
 NA 
 
 
 
 
 chain2 
 
 
  
 Mean 
 SD 
 Naive SE 
 Time-series SE 
 Effective Size 
 HPD95Lower 
 HPD95Upper 
 
 
 
 
 lnL 
 666.4546797 
 932.9969059 
 1.3187963 
 509.361964 
 3.355115 
 194.7579414 
 3186.2540644 
 
 
 prior 
 -102.3526238 
 38.0175721 
 0.0537381 
 3.632901 
 109.511957 
 -192.2762596 
 -67.8976336 
 
 
 alpha 
 64.0992601 
 93.8180351 
 0.1326123 
 60.019601 
 2.443354 
 3.4180938 
 243.4524576 
 
 
 sigma^2 
 0.0561179 
 0.0525918 
 0.0000743 
 0.024788 
 4.501452 
 0.0000000 
 0.1594299 
 
 
 shift number 
 16.3551182 
 7.7078573 
 0.0108951 
 0.651271 
 140.069597 
 9.0000000 
 34.0000000 
 
 
 N theta 
 17.3551182 
 7.7078573 
 0.0108951 
 0.651271 
 140.069597 
 10.0000000 
 35.0000000 
 
 
 root theta 
 -0.9353539 
 0.3505115 
 0.0004954 
 0.023291 
 226.478610 
 -1.6639440 
 -0.3997513 
 
 
 root beta 
 0.7202371 
 0.1426225 
 0.0002016 
 0.006797 
 440.287942 
 0.5534514 
 1.0253371 
 
 
 all theta 
 -1.1417433 
 0.8053504 
 NA 
 NA 
 NA 
 NA 
 NA 
 
 
 all beta 
 0.8048163 
 0.3516405 
 NA 
 NA 
 NA 
 NA 
 NA 
 
 
 
 
 combined chains 
 
 
  
 Mean 
 SD 
 Naive SE 
 Time-series SE 
 Effective Size 
 HPD95Lower 
 HPD95Upper 
 
 
 
 
 lnL 
 545.7824104 
 811.1537182 
 0.9690270 
 384.6283192 
 4.44758 
 214.3624018 
 2823.6849728 
 
 
 prior 
 -96.5382603 
 33.6101817 
 0.0401517 
 2.7978053 
 144.31343 
 -183.1634318 
 -66.3568036 
 
 
 alpha 
 49.5266984 
 82.9385960 
 0.0990808 
 40.9536624 
 4.10136 
 1.2302728 
 242.7536655 
 
 
 sigma^2 
 0.0581702 
 0.0577500 
 0.0000690 
 0.0133303 
 18.76836 
 0.0000000 
 0.1640829 
 
 
 shift number 
 15.2770870 
 6.8007837 
 0.0081244 
 0.4965903 
 187.55191 
 8.0000000 
 32.0000000 
 
 
 N theta 
 16.2770870 
 6.8007837 
 0.0081244 
 0.4965903 
 187.55191 
 9.0000000 
 33.0000000 
 
 
 root theta 
 -0.8795743 
 0.3046272 
 0.0003639 
 0.0165234 
 339.89058 
 -1.6642355 
 -0.4587519 
 
 
 root beta 
 0.6956257 
 0.1332446 
 0.0001592 
 0.0052147 
 652.89661 
 0.5450482 
 0.9923559 
 
 
 all theta 
 -1.0893371 
 0.7624987 
 NA 
 NA 
 NA 
 NA 
 NA 
 
 
 all beta 
 0.7957006 
 0.3246703 
 NA 
 NA 
 NA 
 NA 
 NA 
 
 
 
 
 
 Shifts 
      
 A shows the locations of the selection regimes on the phylogeny. B shows the optimum regression lines associated with each regime. C and D show the posterior distribution of  \(\beta\)  and  \(\theta\)  values associated with each regime. 
   
 Plot shows the median values for parameters associated with each selection regime, along with the posterior probability associeated with each regime.  \(\theta\)  is the intercept of the optimum regression line, and  \(\beta\)  is the coefficient of the predictor. 
 
 
 OU parameters 
 
 
 
  
 chain1 
 chain2 
 combined chains 
 
 
 
 
 alpha 
 10.2791710 
 64.0992601 
 49.5266984 
 
 
 sigma squared 
 0.0504784 
 0.0561179 
 0.0581702 
 
 
 stationary variance 
 0.0024554 
 0.0004377 
 0.0005873 
 
 
 phylogenetic halflife 
 0.0674322 
 0.0108137 
 0.0139954 
 
 
 phylogenetic half life in units of tree height 
 0.0009221 
 0.0001479 
 0.0001914 
 
 
 
 
 
 
 OU Weighted Predictor 
 
 Convergence 
   
 Plots show Gelman’s R statistics for log likelihood,  \(\alpha\) , and  \(\sigma^2\) . Solid lines indicate values Gelman’s R, and dashed lines indicate 95% CIs. 
 
 
 Likelihood 
   
 Plot shows the posterior probabilities of a shift occuring on each branch for each chain prior to merging. 
 
 
 Summary Statistics 
 
 chain1 
 
 
  
 Mean 
 SD 
 Naive SE 
 Time-series SE 
 Effective Size 
 HPD95Lower 
 HPD95Upper 
 
 
 
 
 lnL 
 205.9727727 
 3109.8776608 
 4.3958294 
 24.2784040 
 16407.64844 
 200.1220614 
 255.3505572 
 
 
 prior 
 -93.7790313 
 16.0963880 
 0.0227523 
 0.7439134 
 468.17920 
 -124.7413254 
 -65.6647883 
 
 
 alpha 
 0.1793578 
 0.3604229 
 0.0005095 
 0.0092843 
 1507.05208 
 0.0120895 
 0.6594773 
 
 
 sigma^2 
 0.0011933 
 0.0016560 
 0.0000023 
 0.0000879 
 355.17360 
 0.0003434 
 0.0033960 
 
 
 shift number 
 15.8801701 
 3.5821213 
 0.0050633 
 0.1412347 
 643.27646 
 9.0000000 
 22.0000000 
 
 
 N theta 
 16.8801701 
 3.5821213 
 0.0050633 
 0.1412347 
 643.27646 
 10.0000000 
 23.0000000 
 
 
 root theta 
 -0.9365646 
 0.1171756 
 0.0001656 
 0.0085537 
 187.65772 
 -1.1516056 
 -0.7076672 
 
 
 root beta 
 0.7117140 
 0.0495784 
 0.0000701 
 0.0052052 
 90.72048 
 0.6167695 
 0.8004940 
 
 
 all theta 
 -1.1249988 
 0.8252109 
 NA 
 NA 
 NA 
 NA 
 NA 
 
 
 all beta 
 0.8155196 
 0.3259149 
 NA 
 NA 
 NA 
 NA 
 NA 
 
 
 
 
 chain2 
 
 
  
 Mean 
 SD 
 Naive SE 
 Time-series SE 
 Effective Size 
 HPD95Lower 
 HPD95Upper 
 
 
 
 
 lnL 
 237.9578950 
 216.6191804 
 0.3061924 
 2.2183967 
 9534.883311 
 223.4885566 
 257.9912425 
 
 
 prior 
 -82.3162170 
 9.9562004 
 0.0140731 
 0.4995316 
 397.247712 
 -101.0524954 
 -63.8737574 
 
 
 alpha 
 3.7915154 
 4.1301477 
 0.0058380 
 0.5583518 
 54.716133 
 0.1117776 
 12.4305982 
 
 
 sigma^2 
 0.0187940 
 0.0201610 
 0.0000285 
 0.0076801 
 6.891108 
 0.0006779 
 0.0625128 
 
 
 shift number 
 13.0956042 
 2.2711927 
 0.0032103 
 0.1045702 
 471.728476 
 9.0000000 
 17.0000000 
 
 
 N theta 
 14.0956042 
 2.2711927 
 0.0032103 
 0.1045702 
 471.728476 
 10.0000000 
 18.0000000 
 
 
 root theta 
 -0.9874591 
 0.2634702 
 0.0003724 
 0.0169862 
 240.586182 
 -1.3015174 
 -0.5409743 
 
 
 root beta 
 0.7294942 
 0.0918124 
 0.0001298 
 0.0060437 
 230.775367 
 0.5766925 
 0.8602008 
 
 
 all theta 
 -1.0165693 
 0.6576055 
 NA 
 NA 
 NA 
 NA 
 NA 
 
 
 all beta 
 0.7712305 
 0.2541231 
 NA 
 NA 
 NA 
 NA 
 NA 
 
 
 
 
 combined chains 
 
 
  
 Mean 
 SD 
 Naive SE 
 Time-series SE 
 Effective Size 
 HPD95Lower 
 HPD95Upper 
 
 
 
 
 lnL 
 238.1775393 
 9.5775587 
 0.0114416 
 0.4823353 
 394.28623 
 219.6433164 
 257.2940222 
 
 
 prior 
 -85.1495045 
 12.9606547 
 0.0154832 
 0.5806385 
 498.24416 
 -111.9806970 
 -62.1644231 
 
 
 alpha 
 1.0746598 
 1.8739581 
 0.0022387 
 0.2582479 
 52.65578 
 0.0187830 
 5.8819767 
 
 
 sigma^2 
 0.0053300 
 0.0087385 
 0.0000104 
 0.0024885 
 12.33084 
 0.0003362 
 0.0285592 
 
 
 shift number 
 14.0672495 
 3.0108756 
 0.0035969 
 0.1262370 
 568.86927 
 9.0000000 
 20.0000000 
 
 
 N theta 
 15.0672495 
 3.0108756 
 0.0035969 
 0.1262370 
 568.86927 
 10.0000000 
 21.0000000 
 
 
 root theta 
 -0.9511648 
 0.2148681 
 0.0002567 
 0.0116788 
 338.48919 
 -1.2453660 
 -0.5577720 
 
 
 root beta 
 0.7183411 
 0.0708559 
 0.0000846 
 0.0043567 
 264.50556 
 0.5958968 
 0.8487927 
 
 
 all theta 
 -1.0263472 
 0.6909202 
 NA 
 NA 
 NA 
 NA 
 NA 
 
 
 all beta 
 0.7907559 
 0.2768146 
 NA 
 NA 
 NA 
 NA 
 NA 
 
 
 
 
 
 Shifts 
      
 A shows the locations of the selection regimes on the phylogeny. B shows the optimum regression lines associated with each regime. C and D show the posterior distribution of  \(\beta\)  and  \(\theta\)  values associated with each regime. 
   
 Plot shows the median values for parameters associated with each selection regime, along with the posterior probability associeated with each regime.  \(\theta\)  is the intercept of the optimum regression line, and  \(\beta\)  is the coefficient of the predictor. 
 
 
 
 OU parameters 
 
 
 
  
 chain1 
 chain2 
 combined chains 
 
 
 
 
 alpha 
 0.1793578 
 3.7915154 
 1.0746598 
 
 
 sigma squared 
 0.0011933 
 0.0187940 
 0.0053300 
 
 
 stationary variance 
 0.0033265 
 0.0024784 
 0.0024798 
 
 
 phylogenetic halflife 
 3.8646054 
 0.1828153 
 0.6449922 
 
 
 phylogenetic half life in units of tree height 
 0.0528462 
 0.0024999 
 0.0088199 
 
 
 
 
 
 BM 
 
 Convergence 
   
 Plots show Gelman’s R statistics for log likelihood,  \(\alpha\) , and  \(\sigma^2\) . Solid lines indicate values Gelman’s R, and dashed lines indicate 95% CIs. 
 
 
 Likelihood 
   
 Plot shows the posterior probabilities of a shift occuring on each branch for each chain prior to merging. 
 
 
 Summary Statistics 
 
 chain1 
 
 
  
 Mean 
 SD 
 Naive SE 
 Time-series SE 
 Effective Size 
 HPD95Lower 
 HPD95Upper 
 
 
 
 
 lnL 
 163.1894353 
 12.0707883 
 0.0170621 
 0.5242884 
 530.0670 
 161.1020518 
 165.1376862 
 
 
 prior 
 0.2803633 
 0.1566279 
 0.0002214 
 0.0050543 
 960.3272 
 0.0165250 
 0.5243048 
 
 
 alpha 
 0.0000010 
 0.0000000 
 0.0000000 
 0.0000000 
 0.0000 
 0.0000010 
 0.0000010 
 
 
 sigma^2 
 0.0013045 
 0.0240732 
 0.0000340 
 0.0004710 
 2612.4653 
 0.0006245 
 0.0009925 
 
 
 shift number 
 0.0000000 
 0.0000000 
 0.0000000 
 0.0000000 
 0.0000 
 0.0000000 
 0.0000000 
 
 
 N theta 
 1.0000000 
 0.0000000 
 0.0000000 
 0.0000000 
 0.0000 
 1.0000000 
 1.0000000 
 
 
 root theta 
 -0.5586863 
 0.1427652 
 0.0002018 
 0.0047490 
 903.7194 
 -0.8350485 
 -0.2824194 
 
 
 root beta 
 0.5938608 
 0.0336408 
 0.0000476 
 0.0007124 
 2229.9361 
 0.5289287 
 0.6570275 
 
 
 all theta 
 -0.5586863 
 0.1427652 
 NA 
 NA 
 NA 
 NA 
 NA 
 
 
 all beta 
 0.5938608 
 0.0336408 
 NA 
 NA 
 NA 
 NA 
 NA 
 
 
 
 
 chain2 
 
 
  
 Mean 
 SD 
 Naive SE 
 Time-series SE 
 Effective Size 
 HPD95Lower 
 HPD95Upper 
 
 
 
 
 lnL 
 163.2535340 
 11.1414943 
 0.0157486 
 0.4335143 
 660.5109 
 161.1728961 
 165.1364740 
 
 
 prior 
 0.2831262 
 0.1539420 
 0.0002176 
 0.0046095 
 1115.3272 
 0.0191449 
 0.5178852 
 
 
 alpha 
 0.0000010 
 0.0000000 
 0.0000000 
 0.0000000 
 0.0000 
 0.0000010 
 0.0000010 
 
 
 sigma^2 
 0.0012573 
 0.0240250 
 0.0000340 
 0.0004579 
 2753.1532 
 0.0006219 
 0.0009859 
 
 
 shift number 
 0.0000000 
 0.0000000 
 0.0000000 
 0.0000000 
 0.0000 
 0.0000000 
 0.0000000 
 
 
 N theta 
 1.0000000 
 0.0000000 
 0.0000000 
 0.0000000 
 0.0000 
 1.0000000 
 1.0000000 
 
 
 root theta 
 -0.5610413 
 0.1417960 
 0.0002004 
 0.0046804 
 917.8314 
 -0.8330775 
 -0.2849565 
 
 
 root beta 
 0.5942846 
 0.0330173 
 0.0000467 
 0.0006966 
 2246.6902 
 0.5304492 
 0.6581839 
 
 
 all theta 
 -0.5610413 
 0.1417960 
 NA 
 NA 
 NA 
 NA 
 NA 
 
 
 all beta 
 0.5942846 
 0.0330173 
 NA 
 NA 
 NA 
 NA 
 NA 
 
 
 
 
 combined chains 
 
 
  
 Mean 
 SD 
 Naive SE 
 Time-series SE 
 Effective Size 
 HPD95Lower 
 HPD95Upper 
 
 
 
 
 lnL 
 163.5992852 
 1.2498141 
 0.0014931 
 0.0144825 
 7447.335 
 161.1426428 
 165.1362301 
 
 
 prior 
 0.2854568 
 0.1303117 
 0.0001557 
 0.0036554 
 1270.833 
 0.0173953 
 0.5205409 
 
 
 alpha 
 0.0000010 
 0.0000000 
 0.0000000 
 0.0000000 
 0.000 
 0.0000010 
 0.0000010 
 
 
 sigma^2 
 0.0007979 
 0.0000938 
 0.0000001 
 0.0000009 
 10645.040 
 0.0006221 
 0.0009855 
 
 
 shift number 
 0.0000000 
 0.0000000 
 0.0000000 
 0.0000000 
 0.000 
 0.0000000 
 0.0000000 
 
 
 N theta 
 1.0000000 
 0.0000000 
 0.0000000 
 0.0000000 
 0.000 
 1.0000000 
 1.0000000 
 
 
 root theta 
 -0.5610922 
 0.1401196 
 0.0001674 
 0.0038897 
 1297.652 
 -0.8331344 
 -0.2837343 
 
 
 root beta 
 0.5943018 
 0.0326535 
 0.0000390 
 0.0005712 
 3268.229 
 0.5300882 
 0.6579688 
 
 
 all theta 
 -0.5610922 
 0.1401196 
 NA 
 NA 
 NA 
 NA 
 NA 
 
 
 all beta 
 0.5943018 
 0.0326535 
 NA 
 NA 
 NA 
 NA 
 NA 
 
 
 
 
 
 Shifts 
No shifts were detected with a posterior probability above the cutoff.
 
 OU parameters 
 
 
 
  
 chain1 
 chain2 
 combined chains 
 
 
 
 
 alpha 
 1.000000e-06 
 1.000000e-06 
 1.000000e-06 
 
 
 sigma squared 
 1.304500e-03 
 1.257300e-03 
 7.979000e-04 
 
 
 stationary variance 
 6.522382e+02 
 6.286470e+02 
 3.989616e+02 
 
 
 phylogenetic halflife 
 6.931472e+05 
 6.931472e+05 
 6.931472e+05 
 
 
 phylogenetic half life in units of tree height 
 9.478386e+03 
 9.478386e+03 
 9.478386e+03 
 
 
 
 
 
 Model Selection 
 
 Bayes Factors 
 
 
  
 OU un-weighted predictor 
 OU weighted predictor 
 BM 
 
 
 
 
 OU un-weighted predictor 
 0.00000 
 -65.92240 
 -42.24757 
 
 
 OU weighted predictor 
 65.92240 
 0.00000 
 23.67484 
 
 
 BM 
 42.24757 
 -23.67484 
 0.00000 
 
 
 
 
 
 
 ECV 
 
 OU Un-weighted Predictor 
 
 Convergence 
   
 Plots show Gelman’s R statistics for log likelihood,  \(\alpha\) , and  \(\sigma^2\) . Solid lines indicate values Gelman’s R, and dashed lines indicate 95% CIs. 
 
 
 Likelihood 
   
 Plot shows the posterior probabilities of a shift occuring on each branch for each chain prior to merging. 
 
 
 Summary Statistics 
 
 chain1 
 
 
  
 Mean 
 SD 
 Naive SE 
 Time-series SE 
 Effective Size 
 HPD95Lower 
 HPD95Upper 
 
 
 
 
 lnL 
 174.6214372 
 21.7986946 
 0.0308126 
 1.9473736 
 125.303279 
 139.1266144 
 211.3312720 
 
 
 prior 
 -147.8590327 
 14.9149016 
 0.0210823 
 0.5350832 
 776.959223 
 -176.3518343 
 -117.9163615 
 
 
 alpha 
 3.6219062 
 6.6094932 
 0.0093426 
 1.3015221 
 25.788922 
 0.0389104 
 18.8488394 
 
 
 sigma^2 
 0.0353459 
 0.0631765 
 0.0000893 
 0.0256385 
 6.071904 
 0.0009980 
 0.1702755 
 
 
 shift number 
 28.4165966 
 3.5212424 
 0.0049773 
 0.1223056 
 828.894222 
 22.0000000 
 35.0000000 
 
 
 N theta 
 29.4165966 
 3.5212424 
 0.0049773 
 0.1223056 
 828.894222 
 23.0000000 
 36.0000000 
 
 
 root theta 
 1.3480445 
 0.5757473 
 0.0008138 
 0.0405189 
 201.905978 
 0.4447422 
 1.9137552 
 
 
 root beta 
 -0.0311308 
 0.2131406 
 0.0003013 
 0.0129118 
 272.495534 
 -0.5124734 
 0.4104057 
 
 
 all theta 
 1.4944570 
 0.8417976 
 NA 
 NA 
 NA 
 NA 
 NA 
 
 
 all beta 
 -0.0102943 
 0.3882724 
 NA 
 NA 
 NA 
 NA 
 NA 
 
 
 
 
 chain2 
 
 
  
 Mean 
 SD 
 Naive SE 
 Time-series SE 
 Effective Size 
 HPD95Lower 
 HPD95Upper 
 
 
 
 
 lnL 
 181.4026098 
 27.7985558 
 0.0392934 
 3.4180908 
 66.141993 
 129.0944327 
 221.7753231 
 
 
 prior 
 -150.1209745 
 15.3975558 
 0.0217645 
 0.6050480 
 647.625366 
 -181.0985482 
 -118.7383513 
 
 
 alpha 
 4.0728791 
 5.4190179 
 0.0076598 
 0.8457653 
 41.052670 
 0.0297459 
 16.1351651 
 
 
 sigma^2 
 0.0365275 
 0.0463503 
 0.0000655 
 0.0218552 
 4.497754 
 0.0012069 
 0.1355008 
 
 
 shift number 
 28.8274669 
 3.6433570 
 0.0051499 
 0.1407473 
 670.074766 
 20.0000000 
 35.0000000 
 
 
 N theta 
 29.8274669 
 3.6433570 
 0.0051499 
 0.1407473 
 670.074766 
 21.0000000 
 36.0000000 
 
 
 root theta 
 1.4650016 
 0.6003564 
 0.0008486 
 0.0394836 
 231.198679 
 0.3937086 
 2.5150004 
 
 
 root beta 
 0.0050696 
 0.2380053 
 0.0003364 
 0.0149054 
 254.966903 
 -0.5038056 
 0.4760795 
 
 
 all theta 
 1.4828809 
 0.8080249 
 NA 
 NA 
 NA 
 NA 
 NA 
 
 
 all beta 
 0.0274549 
 0.4055927 
 NA 
 NA 
 NA 
 NA 
 NA 
 
 
 
 
 combined chains 
 
 
  
 Mean 
 SD 
 Naive SE 
 Time-series SE 
 Effective Size 
 HPD95Lower 
 HPD95Upper 
 
 
 
 
 lnL 
 171.7258943 
 22.1196797 
 0.0264248 
 2.3800482 
 86.374621 
 131.5196106 
 212.2920745 
 
 
 prior 
 -147.5622771 
 16.2029266 
 0.0193565 
 0.4724873 
 1175.997978 
 -179.1930662 
 -115.6217879 
 
 
 alpha 
 0.7101747 
 1.1182121 
 0.0013358 
 0.1828494 
 37.399117 
 0.0335257 
 3.1631011 
 
 
 sigma^2 
 0.0070453 
 0.0090573 
 0.0000108 
 0.0034185 
 7.019986 
 0.0009930 
 0.0279767 
 
 
 shift number 
 28.2572627 
 3.7563094 
 0.0044874 
 0.1055087 
 1267.495319 
 21.0000000 
 35.0000000 
 
 
 N theta 
 29.2572627 
 3.7563094 
 0.0044874 
 0.1055087 
 1267.495319 
 22.0000000 
 36.0000000 
 
 
 root theta 
 1.3838629 
 0.5783772 
 0.0006909 
 0.0354791 
 265.753051 
 0.4353998 
 1.9268027 
 
 
 root beta 
 -0.0019358 
 0.1962923 
 0.0002345 
 0.0096032 
 417.805872 
 -0.4458041 
 0.3438042 
 
 
 all theta 
 1.4923729 
 0.8636315 
 NA 
 NA 
 NA 
 NA 
 NA 
 
 
 all beta 
 0.0121347 
 0.4083712 
 NA 
 NA 
 NA 
 NA 
 NA 
 
 
 
 
 
 Shifts 
      
 A shows the locations of the selection regimes on the phylogeny. B shows the optimum regression lines associated with each regime. C and D show the posterior distribution of  \(\beta\)  and  \(\theta\)  values associated with each regime. 
   
 Plot shows the median values for parameters associated with each selection regime, along with the posterior probability associeated with each regime.  \(\theta\)  is the intercept of the optimum regression line, and  \(\beta\)  is the coefficient of the predictor. 
 
 
 OU parameters 
 
 
 
  
 chain1 
 chain2 
 combined chains 
 
 
 
 
 alpha 
 3.6219062 
 4.0728791 
 0.7101747 
 
 
 sigma squared 
 0.0353459 
 0.0365275 
 0.0070453 
 
 
 stationary variance 
 0.0048795 
 0.0044842 
 0.0049602 
 
 
 phylogenetic halflife 
 0.1913763 
 0.1701860 
 0.9760235 
 
 
 phylogenetic half life in units of tree height 
 0.0026215 
 0.0023312 
 0.0133696 
 
 
 
 
 
 
 BM 
 
 Convergence 
   
 Plots show Gelman’s R statistics for log likelihood,  \(\alpha\) , and  \(\sigma^2\) . Solid lines indicate values Gelman’s R, and dashed lines indicate 95% CIs. 
 
 
 Likelihood 
   
 Plot shows the posterior probabilities of a shift occuring on each branch for each chain prior to merging. 
 
 
 Summary Statistics 
 
 chain1 
 
 
  
 Mean 
 SD 
 Naive SE 
 Time-series SE 
 Effective Size 
 HPD95Lower 
 HPD95Upper 
 
 
 
 
 lnL 
 77.3453046 
 5.0946299 
 0.0072013 
 0.0977274 
 2717.64263 
 75.4206236 
 78.4466005 
 
 
 prior 
 0.1350335 
 0.6554343 
 0.0009265 
 0.0799092 
 67.27672 
 -1.2538328 
 0.7055855 
 
 
 alpha 
 0.0000010 
 0.0000000 
 0.0000000 
 0.0000000 
 0.00000 
 0.0000010 
 0.0000010 
 
 
 sigma^2 
 0.0027076 
 0.0127084 
 0.0000180 
 0.0001405 
 8184.67285 
 0.0019890 
 0.0031682 
 
 
 shift number 
 0.0000000 
 0.0000000 
 0.0000000 
 0.0000000 
 0.00000 
 0.0000000 
 0.0000000 
 
 
 N theta 
 1.0000000 
 0.0000000 
 0.0000000 
 0.0000000 
 0.00000 
 1.0000000 
 1.0000000 
 
 
 root theta 
 1.2268522 
 0.1783562 
 0.0002521 
 0.0073183 
 593.96650 
 0.8796723 
 1.5778747 
 
 
 root beta 
 -0.1690987 
 0.4411284 
 0.0006235 
 0.0857306 
 26.47637 
 -1.0153639 
 0.6921253 
 
 
 all theta 
 1.2268522 
 0.1783562 
 NA 
 NA 
 NA 
 NA 
 NA 
 
 
 all beta 
 -0.1690987 
 0.4411284 
 NA 
 NA 
 NA 
 NA 
 NA 
 
 
 
 
 chain2 
 
 
  
 Mean 
 SD 
 Naive SE 
 Time-series SE 
 Effective Size 
 HPD95Lower 
 HPD95Upper 
 
 
 
 
 lnL 
 77.2992166 
 4.6730409 
 0.0066054 
 0.1053644 
 1967.03292 
 75.2779899 
 78.4465954 
 
 
 prior 
 -0.0150455 
 0.7236895 
 0.0010229 
 0.0919432 
 61.95338 
 -1.5896430 
 0.7046351 
 
 
 alpha 
 0.0000010 
 0.0000000 
 0.0000000 
 0.0000000 
 0.00000 
 0.0000010 
 0.0000010 
 
 
 sigma^2 
 0.0026485 
 0.0083585 
 0.0000118 
 0.0000870 
 9237.90501 
 0.0020093 
 0.0031712 
 
 
 shift number 
 0.0000000 
 0.0000000 
 0.0000000 
 0.0000000 
 0.00000 
 0.0000000 
 0.0000000 
 
 
 N theta 
 1.0000000 
 0.0000000 
 0.0000000 
 0.0000000 
 0.00000 
 1.0000000 
 1.0000000 
 
 
 root theta 
 1.2185557 
 0.1894400 
 0.0002678 
 0.0082231 
 530.73390 
 0.8534836 
 1.5921821 
 
 
 root beta 
 0.0142040 
 0.5432906 
 0.0007679 
 0.1292278 
 17.67472 
 -1.0551329 
 1.0040725 
 
 
 all theta 
 1.2185557 
 0.1894400 
 NA 
 NA 
 NA 
 NA 
 NA 
 
 
 all beta 
 0.0142040 
 0.5432906 
 NA 
 NA 
 NA 
 NA 
 NA 
 
 
 
 
 combined chains 
 
 
  
 Mean 
 SD 
 Naive SE 
 Time-series SE 
 Effective Size 
 HPD95Lower 
 HPD95Upper 
 
 
 
 
 lnL 
 77.4049901 
 1.0332654 
 0.0012344 
 0.0148397 
 4848.11431 
 75.3612431 
 78.4466005 
 
 
 prior 
 0.0474492 
 0.6836918 
 0.0008168 
 0.0753117 
 82.41295 
 -1.4653720 
 0.7055366 
 
 
 alpha 
 0.0000010 
 0.0000000 
 0.0000000 
 0.0000000 
 0.00000 
 0.0000010 
 0.0000010 
 
 
 sigma^2 
 0.0025608 
 0.0003037 
 0.0000004 
 0.0000013 
 57498.47407 
 0.0019968 
 0.0031664 
 
 
 shift number 
 0.0000000 
 0.0000000 
 0.0000000 
 0.0000000 
 0.00000 
 0.0000000 
 0.0000000 
 
 
 N theta 
 1.0000000 
 0.0000000 
 0.0000000 
 0.0000000 
 0.00000 
 1.0000000 
 1.0000000 
 
 
 root theta 
 1.2293587 
 0.1841855 
 0.0002200 
 0.0065921 
 780.65078 
 0.8748883 
 1.5921417 
 
 
 root beta 
 -0.0617955 
 0.5133787 
 0.0006133 
 0.0966758 
 28.19940 
 -1.0810888 
 0.9005571 
 
 
 all theta 
 1.2293587 
 0.1841855 
 NA 
 NA 
 NA 
 NA 
 NA 
 
 
 all beta 
 -0.0617955 
 0.5133787 
 NA 
 NA 
 NA 
 NA 
 NA 
 
 
 
 
 
 Shifts 
No shifts were detected with a posterior probability above the cutoff.
 
 OU parameters 
 
 
 
  
 chain1 
 chain2 
 combined chains 
 
 
 
 
 alpha 
 1.000000e-06 
 1.000000e-06 
 1.000000e-06 
 
 
 sigma squared 
 2.707600e-03 
 2.648500e-03 
 2.560800e-03 
 
 
 stationary variance 
 1.353775e+03 
 1.324266e+03 
 1.280384e+03 
 
 
 phylogenetic halflife 
 6.931472e+05 
 6.931472e+05 
 6.931472e+05 
 
 
 phylogenetic half life in units of tree height 
 9.494761e+03 
 9.494761e+03 
 9.494761e+03 
 
 
 
 
 
 Model Selection 
 
 Bayes Factors 
 
 
  
 OU un-weighted predictor 
 BM 
 
 
 
 
 OU un-weighted predictor 
 0.00000 
 10.33027 
 
 
 BM 
 -10.33027 
 0.00000 
 
 
 
 
 
 
 ECV (alternate hominin phylogeny) 
 
 OU Un-weighted Predictor 
 
 Convergence 
   
 Plots show Gelman’s R statistics for log likelihood,  \(\alpha\) , and  \(\sigma^2\) . Solid lines indicate values Gelman’s R, and dashed lines indicate 95% CIs. 
 
 
 Likelihood 
   
 Plot shows the posterior probabilities of a shift occuring on each branch for each chain prior to merging. 
 
 
 Summary Statistics 
 
 chain1 
 
 
  
 Mean 
 SD 
 Naive SE 
 Time-series SE 
 Effective Size 
 HPD95Lower 
 HPD95Upper 
 
 
 
 
 lnL 
 176.9476542 
 21.3921933 
 0.0302380 
 1.7686473 
 146.29455 
 142.0030639 
 218.2957649 
 
 
 prior 
 -152.6639443 
 16.3640578 
 0.0231307 
 0.6161128 
 705.44247 
 -184.2026613 
 -119.7086741 
 
 
 alpha 
 1.1211028 
 3.3728059 
 0.0047675 
 0.6561153 
 26.42545 
 0.0389917 
 8.6316867 
 
 
 sigma^2 
 0.0137244 
 0.0452582 
 0.0000640 
 0.0079137 
 32.70622 
 0.0008221 
 0.0981914 
 
 
 shift number 
 29.4152879 
 3.9301172 
 0.0055552 
 0.1375691 
 816.14807 
 22.0000000 
 37.0000000 
 
 
 N theta 
 30.4152879 
 3.9301172 
 0.0055552 
 0.1375691 
 816.14807 
 23.0000000 
 38.0000000 
 
 
 root theta 
 1.2581681 
 0.5319136 
 0.0007519 
 0.0336661 
 249.62932 
 0.5023069 
 1.9053142 
 
 
 root beta 
 -0.0267969 
 0.1915037 
 0.0002707 
 0.0106305 
 324.52133 
 -0.4016019 
 0.3530201 
 
 
 all theta 
 1.5048462 
 0.8945219 
 NA 
 NA 
 NA 
 NA 
 NA 
 
 
 all beta 
 0.0029253 
 0.3997772 
 NA 
 NA 
 NA 
 NA 
 NA 
 
 
 
 
 chain2 
 
 
  
 Mean 
 SD 
 Naive SE 
 Time-series SE 
 Effective Size 
 HPD95Lower 
 HPD95Upper 
 
 
 
 
 lnL 
 172.4067062 
 25.8575056 
 0.0365497 
 2.8417075 
 82.79698 
 122.7756336 
 215.0405743 
 
 
 prior 
 -151.7218012 
 16.1925867 
 0.0228883 
 0.5479957 
 873.12840 
 -185.5566625 
 -121.5529627 
 
 
 alpha 
 0.5826597 
 1.7719813 
 0.0025047 
 0.3713733 
 22.76653 
 0.0240476 
 3.7040336 
 
 
 sigma^2 
 0.0061988 
 0.0160970 
 0.0000228 
 0.0035815 
 20.20068 
 0.0008460 
 0.0394493 
 
 
 shift number 
 28.9683098 
 3.8842489 
 0.0054904 
 0.1307877 
 882.02319 
 21.0000000 
 36.0000000 
 
 
 N theta 
 29.9683098 
 3.8842489 
 0.0054904 
 0.1307877 
 882.02319 
 22.0000000 
 37.0000000 
 
 
 root theta 
 1.3741725 
 0.5605885 
 0.0007924 
 0.0371864 
 227.25788 
 0.4623881 
 1.9165479 
 
 
 root beta 
 0.0014975 
 0.1977468 
 0.0002795 
 0.0103962 
 361.80022 
 -0.3735185 
 0.3913545 
 
 
 all theta 
 1.4749374 
 0.9179613 
 NA 
 NA 
 NA 
 NA 
 NA 
 
 
 all beta 
 -0.0059581 
 0.4061569 
 NA 
 NA 
 NA 
 NA 
 NA 
 
 
 
 
 combined chains 
 
 
  
 Mean 
 SD 
 Naive SE 
 Time-series SE 
 Effective Size 
 HPD95Lower 
 HPD95Upper 
 
 
 
 
 lnL 
 170.0996299 
 20.3578485 
 0.0243201 
 1.9309192 
 111.1567 
 123.5051358 
 206.8698422 
 
 
 prior 
 -152.4099065 
 16.7462273 
 0.0200055 
 0.4719433 
 1259.0829 
 -185.8307039 
 -119.0731173 
 
 
 alpha 
 0.0920603 
 0.0271479 
 0.0000324 
 0.0026513 
 104.8449 
 0.0391477 
 0.1455830 
 
 
 sigma^2 
 0.0016867 
 0.0003742 
 0.0000004 
 0.0000246 
 231.7616 
 0.0010345 
 0.0024136 
 
 
 shift number 
 29.0411871 
 3.9977269 
 0.0047758 
 0.1117011 
 1280.8895 
 22.0000000 
 37.0000000 
 
 
 N theta 
 30.0411871 
 3.9977269 
 0.0047758 
 0.1117011 
 1280.8895 
 23.0000000 
 38.0000000 
 
 
 root theta 
 1.2980816 
 0.5285026 
 0.0006314 
 0.0313604 
 284.0089 
 0.5057147 
 1.9101070 
 
 
 root beta 
 -0.0061110 
 0.1884236 
 0.0002251 
 0.0086320 
 476.4789 
 -0.3761089 
 0.3411697 
 
 
 all theta 
 1.4905268 
 0.9460229 
 NA 
 NA 
 NA 
 NA 
 NA 
 
 
 all beta 
 0.0042702 
 0.4064725 
 NA 
 NA 
 NA 
 NA 
 NA 
 
 
 
 
 
 Shifts 
      
 A shows the locations of the selection regimes on the phylogeny. B shows the optimum regression lines associated with each regime. C and D show the posterior distribution of  \(\beta\)  and  \(\theta\)  values associated with each regime. 
   
 Plot shows the median values for parameters associated with each selection regime, along with the posterior probability associeated with each regime.  \(\theta\)  is the intercept of the optimum regression line, and  \(\beta\)  is the coefficient of the predictor. 
 
 
 OU parameters 
 
 
 
  
 chain1 
 chain2 
 combined chains 
 
 
 
 
 alpha 
 1.1211028 
 0.5826597 
 0.0920603 
 
 
 sigma squared 
 0.0137244 
 0.0061988 
 0.0016867 
 
 
 stationary variance 
 0.0061209 
 0.0053194 
 0.0091606 
 
 
 phylogenetic halflife 
 0.6182726 
 1.1896261 
 7.5292716 
 
 
 phylogenetic half life in units of tree height 
 0.0084545 
 0.0162674 
 0.1029584 
 
 
 
 
 
 
 BM 
 
 Convergence 
   
 Plots show Gelman’s R statistics for log likelihood,  \(\alpha\) , and  \(\sigma^2\) . Solid lines indicate values Gelman’s R, and dashed lines indicate 95% CIs. 
 
 
 Likelihood 
   
 Plot shows the posterior probabilities of a shift occuring on each branch for each chain prior to merging. 
 
 
 Summary Statistics 
 
 chain1 
 
 
  
 Mean 
 SD 
 Naive SE 
 Time-series SE 
 Effective Size 
 HPD95Lower 
 HPD95Upper 
 
 
 
 
 lnL 
 74.9738061 
 7.0976421 
 0.0100326 
 0.2167094 
 1072.68574 
 73.1575497 
 76.1321140 
 
 
 prior 
 0.0456658 
 0.7074042 
 0.0009999 
 0.0905378 
 61.04861 
 -1.5649174 
 0.7042503 
 
 
 alpha 
 0.0000010 
 0.0000000 
 0.0000000 
 0.0000000 
 0.00000 
 0.0000010 
 0.0000010 
 
 
 sigma^2 
 0.0028452 
 0.0156300 
 0.0000221 
 0.0002838 
 3033.50841 
 0.0020177 
 0.0031884 
 
 
 shift number 
 0.0000000 
 0.0000000 
 0.0000000 
 0.0000000 
 0.00000 
 0.0000000 
 0.0000000 
 
 
 N theta 
 1.0000000 
 0.0000000 
 0.0000000 
 0.0000000 
 0.00000 
 1.0000000 
 1.0000000 
 
 
 root theta 
 1.2287395 
 0.1766699 
 0.0002497 
 0.0071445 
 611.47415 
 0.8791971 
 1.5736220 
 
 
 root beta 
 -0.0537153 
 0.5149835 
 0.0007279 
 0.1173667 
 19.25293 
 -1.0102430 
 1.0547852 
 
 
 all theta 
 1.2287395 
 0.1766699 
 NA 
 NA 
 NA 
 NA 
 NA 
 
 
 all beta 
 -0.0537153 
 0.5149835 
 NA 
 NA 
 NA 
 NA 
 NA 
 
 
 
 
 chain2 
 
 
  
 Mean 
 SD 
 Naive SE 
 Time-series SE 
 Effective Size 
 HPD95Lower 
 HPD95Upper 
 
 
 
 
 lnL 
 74.9768890 
 6.2645802 
 0.0088550 
 0.1565561 
 1601.19357 
 73.1190336 
 76.1321137 
 
 
 prior 
 0.2572294 
 0.5091031 
 0.0007196 
 0.0522164 
 95.06006 
 -0.6035923 
 0.7054618 
 
 
 alpha 
 0.0000010 
 0.0000000 
 0.0000000 
 0.0000000 
 0.00000 
 0.0000010 
 0.0000010 
 
 
 sigma^2 
 0.0027943 
 0.0157252 
 0.0000222 
 0.0001990 
 6244.80830 
 0.0020047 
 0.0031792 
 
 
 shift number 
 0.0000000 
 0.0000000 
 0.0000000 
 0.0000000 
 0.00000 
 0.0000000 
 0.0000000 
 
 
 N theta 
 1.0000000 
 0.0000000 
 0.0000000 
 0.0000000 
 0.00000 
 1.0000000 
 1.0000000 
 
 
 root theta 
 1.2240672 
 0.1831020 
 0.0002588 
 0.0076392 
 574.50031 
 0.8654063 
 1.5774800 
 
 
 root beta 
 -0.0304771 
 0.3998772 
 0.0005652 
 0.0716526 
 31.14504 
 -0.8069548 
 0.7253226 
 
 
 all theta 
 1.2240672 
 0.1831020 
 NA 
 NA 
 NA 
 NA 
 NA 
 
 
 all beta 
 -0.0304771 
 0.3998772 
 NA 
 NA 
 NA 
 NA 
 NA 
 
 
 
 
 combined chains 
 
 
  
 Mean 
 SD 
 Naive SE 
 Time-series SE 
 Effective Size 
 HPD95Lower 
 HPD95Upper 
 
 
 
 
 lnL 
 75.1102665 
 1.0059315 
 0.0012017 
 0.0139145 
 5226.40511 
 73.0838470 
 76.1321140 
 
 
 prior 
 0.1393694 
 0.6454463 
 0.0007711 
 0.0750993 
 73.86674 
 -1.3272661 
 0.7056844 
 
 
 alpha 
 0.0000010 
 0.0000000 
 0.0000000 
 0.0000000 
 0.00000 
 0.0000010 
 0.0000010 
 
 
 sigma^2 
 0.0025748 
 0.0003028 
 0.0000004 
 0.0000021 
 21084.01496 
 0.0020125 
 0.0031804 
 
 
 shift number 
 0.0000000 
 0.0000000 
 0.0000000 
 0.0000000 
 0.00000 
 0.0000000 
 0.0000000 
 
 
 N theta 
 1.0000000 
 0.0000000 
 0.0000000 
 0.0000000 
 0.00000 
 1.0000000 
 1.0000000 
 
 
 root theta 
 1.2290797 
 0.1833525 
 0.0002190 
 0.0064793 
 800.79983 
 0.8665186 
 1.5871384 
 
 
 root beta 
 -0.0806832 
 0.4635894 
 0.0005538 
 0.0800483 
 33.53993 
 -1.1368225 
 0.7192357 
 
 
 all theta 
 1.2290797 
 0.1833525 
 NA 
 NA 
 NA 
 NA 
 NA 
 
 
 all beta 
 -0.0806832 
 0.4635894 
 NA 
 NA 
 NA 
 NA 
 NA 
 
 
 
 
 
 Shifts 
No shifts were detected with a posterior probability above the cutoff.
 
 OU parameters 
 
 
 
  
 chain1 
 chain2 
 combined chains 
 
 
 
 
 alpha 
 1.000000e-06 
 1.000000e-06 
 1.000000e-06 
 
 
 sigma squared 
 2.845200e-03 
 2.794300e-03 
 2.574800e-03 
 
 
 stationary variance 
 1.422615e+03 
 1.397139e+03 
 1.287402e+03 
 
 
 phylogenetic halflife 
 6.931472e+05 
 6.931472e+05 
 6.931472e+05 
 
 
 phylogenetic half life in units of tree height 
 9.478386e+03 
 9.478386e+03 
 9.478386e+03 
 
 
 
 
 
 Model Selection 
 
 Bayes Factors 
 
 
  
 OU un-weighted predictor 
 BM 
 
 
 
 
 OU un-weighted predictor 
 0.00000 
 15.13087 
 
 
 BM 
 -15.13087 
 0.00000 
 
 
 
 
 
 
 Rest of Brain ~ Body Mass 
 
 OU Un-weighted Predictor 
 
 Convergence 
   
 Plots show Gelman’s R statistics for log likelihood,  \(\alpha\) , and  \(\sigma^2\) . Solid lines indicate values Gelman’s R, and dashed lines indicate 95% CIs. 
 
 
 Likelihood 
   
 Plot shows the posterior probabilities of a shift occuring on each branch for each chain prior to merging. 
 
 
 Summary Statistics 
 
 chain1 
 
 
  
 Mean 
 SD 
 Naive SE 
 Time-series SE 
 Effective Size 
 HPD95Lower 
 HPD95Upper 
 
 
 
 
 lnL 
 35.0937340 
 6.0299857 
 0.0085234 
 0.0649782 
 8611.8647 
 30.1929017 
 40.6405504 
 
 
 prior 
 -17.7202080 
 4.9446584 
 0.0069893 
 0.0982557 
 2532.5455 
 -26.7869443 
 -10.1532887 
 
 
 alpha 
 3.0112156 
 4.1903372 
 0.0059231 
 0.3104779 
 182.1531 
 0.0443452 
 8.4652205 
 
 
 sigma^2 
 0.0948241 
 0.1305784 
 0.0001846 
 0.0117059 
 124.4320 
 0.0021275 
 0.2638584 
 
 
 shift number 
 1.3026228 
 1.1294804 
 0.0015965 
 0.0192860 
 3429.8312 
 0.0000000 
 3.0000000 
 
 
 N theta 
 2.3026228 
 1.1294804 
 0.0015965 
 0.0192860 
 3429.8312 
 1.0000000 
 4.0000000 
 
 
 root theta 
 1.7827258 
 0.0872482 
 0.0001233 
 0.0029554 
 871.5519 
 1.6181396 
 1.9561358 
 
 
 root beta 
 0.6365952 
 0.0266114 
 0.0000376 
 0.0009060 
 862.7057 
 0.5853532 
 0.6872355 
 
 
 all theta 
 1.8511293 
 0.6545371 
 NA 
 NA 
 NA 
 NA 
 NA 
 
 
 all beta 
 0.6227724 
 0.1992435 
 NA 
 NA 
 NA 
 NA 
 NA 
 
 
 
 
 chain2 
 
 
  
 Mean 
 SD 
 Naive SE 
 Time-series SE 
 Effective Size 
 HPD95Lower 
 HPD95Upper 
 
 
 
 
 lnL 
 35.0392824 
 4.5948791 
 0.0064949 
 0.0761639 
 3639.5693 
 30.1469878 
 40.5747266 
 
 
 prior 
 -17.1633009 
 4.9348755 
 0.0069755 
 0.0988879 
 2490.3829 
 -25.9686790 
 -10.1514070 
 
 
 alpha 
 2.4245096 
 2.8645725 
 0.0040491 
 0.1765854 
 263.1541 
 0.0521792 
 7.4081067 
 
 
 sigma^2 
 0.0767684 
 0.0908343 
 0.0001284 
 0.0062350 
 212.2385 
 0.0019014 
 0.2338630 
 
 
 shift number 
 1.2701074 
 1.1438839 
 0.0016169 
 0.0194881 
 3445.2968 
 0.0000000 
 3.0000000 
 
 
 N theta 
 2.2701074 
 1.1438839 
 0.0016169 
 0.0194881 
 3445.2968 
 1.0000000 
 4.0000000 
 
 
 root theta 
 1.7751165 
 0.0885335 
 0.0001251 
 0.0030382 
 849.1326 
 1.6009166 
 1.9523789 
 
 
 root beta 
 0.6391139 
 0.0271911 
 0.0000384 
 0.0009071 
 898.5340 
 0.5869471 
 0.6924215 
 
 
 all theta 
 1.8313309 
 0.6389266 
 NA 
 NA 
 NA 
 NA 
 NA 
 
 
 all beta 
 0.6276917 
 0.1910389 
 NA 
 NA 
 NA 
 NA 
 NA 
 
 
 
 
 combined chains 
 
 
  
 Mean 
 SD 
 Naive SE 
 Time-series SE 
 Effective Size 
 HPD95Lower 
 HPD95Upper 
 
 
 
 
 lnL 
 35.1136970 
 2.5579851 
 0.0030558 
 0.0475565 
 2893.1841 
 30.1153025 
 40.5792956 
 
 
 prior 
 -17.3707357 
 4.8374886 
 0.0057790 
 0.0797489 
 3679.5183 
 -26.2353334 
 -10.1527919 
 
 
 alpha 
 2.4883053 
 2.6221166 
 0.0031325 
 0.1341058 
 382.3042 
 0.0584858 
 7.1128795 
 
 
 sigma^2 
 0.0783042 
 0.0805598 
 0.0000962 
 0.0051205 
 247.5177 
 0.0019579 
 0.2218412 
 
 
 shift number 
 1.2927113 
 1.1249445 
 0.0013439 
 0.0167169 
 4528.4773 
 0.0000000 
 3.0000000 
 
 
 N theta 
 2.2927113 
 1.1249445 
 0.0013439 
 0.0167169 
 4528.4773 
 1.0000000 
 4.0000000 
 
 
 root theta 
 1.7789407 
 0.0883499 
 0.0001055 
 0.0025610 
 1190.0935 
 1.6084118 
 1.9534330 
 
 
 root beta 
 0.6378374 
 0.0265910 
 0.0000318 
 0.0007850 
 1147.5760 
 0.5850746 
 0.6888699 
 
 
 all theta 
 1.8429826 
 0.6467617 
 NA 
 NA 
 NA 
 NA 
 NA 
 
 
 all beta 
 0.6250019 
 0.1963772 
 NA 
 NA 
 NA 
 NA 
 NA 
 
 
 
 
 
 Shifts 
No shifts were detected with a posterior probability above the cutoff.
 
 
 OU parameters 
 
 
 
  
 chain1 
 chain2 
 combined chains 
 
 
 
 
 alpha 
 3.0112156 
 2.4245096 
 2.4883053 
 
 
 sigma squared 
 0.0948241 
 0.0767684 
 0.0783042 
 
 
 stationary variance 
 0.0157451 
 0.0158317 
 0.0157344 
 
 
 phylogenetic halflife 
 0.2301885 
 0.2858917 
 0.2785619 
 
 
 phylogenetic half life in units of tree height 
 0.0031531 
 0.0039162 
 0.0038158 
 
 
 
 
 
 OU Weighted Predictor 
 
 Convergence 
   
 Plots show Gelman’s R statistics for log likelihood,  \(\alpha\) , and  \(\sigma^2\) . Solid lines indicate values Gelman’s R, and dashed lines indicate 95% CIs. 
 
 
 Likelihood 
   
 Plot shows the posterior probabilities of a shift occuring on each branch for each chain prior to merging. 
 
 
 Summary Statistics 
 
 chain1 
 
 
  
 Mean 
 SD 
 Naive SE 
 Time-series SE 
 Effective Size 
 HPD95Lower 
 HPD95Upper 
 
 
 
 
 lnL 
 34.9817404 
 34.0805196 
 0.0481730 
 0.2102461 
 26275.83766 
 30.1645086 
 40.9718681 
 
 
 prior 
 -17.4475603 
 5.0030986 
 0.0070719 
 0.0952160 
 2760.94615 
 -26.3314566 
 -10.1604904 
 
 
 alpha 
 2.4104040 
 2.5347325 
 0.0035829 
 0.2158898 
 137.84791 
 0.0996141 
 6.7482358 
 
 
 sigma^2 
 0.0759462 
 0.0787575 
 0.0001113 
 0.0145134 
 29.44729 
 0.0039144 
 0.2100656 
 
 
 shift number 
 1.3282651 
 1.1795765 
 0.0016673 
 0.0206004 
 3278.68844 
 0.0000000 
 3.0000000 
 
 
 N theta 
 2.3282651 
 1.1795765 
 0.0016673 
 0.0206004 
 3278.68844 
 1.0000000 
 4.0000000 
 
 
 root theta 
 1.7829262 
 0.0887056 
 0.0001254 
 0.0029828 
 884.42628 
 1.6125958 
 1.9623709 
 
 
 root beta 
 0.6363281 
 0.0304438 
 0.0000430 
 0.0009794 
 966.27086 
 0.5826177 
 0.6876583 
 
 
 all theta 
 1.8603783 
 0.6456922 
 NA 
 NA 
 NA 
 NA 
 NA 
 
 
 all beta 
 0.6196096 
 0.1963656 
 NA 
 NA 
 NA 
 NA 
 NA 
 
 
 
 
 chain2 
 
 
  
 Mean 
 SD 
 Naive SE 
 Time-series SE 
 Effective Size 
 HPD95Lower 
 HPD95Upper 
 
 
 
 
 lnL 
 35.2581918 
 3.1166710 
 0.0044054 
 0.0677628 
 2115.43009 
 30.1449338 
 41.0102256 
 
 
 prior 
 -17.7353482 
 4.9462905 
 0.0069916 
 0.0997299 
 2459.84792 
 -26.8515410 
 -10.1709847 
 
 
 alpha 
 2.6838008 
 2.4766979 
 0.0035008 
 0.1901318 
 169.68223 
 0.1388916 
 7.6123205 
 
 
 sigma^2 
 0.0840199 
 0.0759003 
 0.0001073 
 0.0122242 
 38.55211 
 0.0053879 
 0.2318710 
 
 
 shift number 
 1.3425088 
 1.1561857 
 0.0016343 
 0.0210889 
 3005.70931 
 0.0000000 
 3.0000000 
 
 
 N theta 
 2.3425088 
 1.1561857 
 0.0016343 
 0.0210889 
 3005.70931 
 1.0000000 
 4.0000000 
 
 
 root theta 
 1.7836321 
 0.0882633 
 0.0001248 
 0.0030532 
 835.67737 
 1.6115112 
 1.9562768 
 
 
 root beta 
 0.6361178 
 0.0270232 
 0.0000382 
 0.0009401 
 826.24225 
 0.5840623 
 0.6882896 
 
 
 all theta 
 1.8569272 
 0.6439409 
 NA 
 NA 
 NA 
 NA 
 NA 
 
 
 all beta 
 0.6208193 
 0.1904165 
 NA 
 NA 
 NA 
 NA 
 NA 
 
 
 
 
 combined chains 
 
 
  
 Mean 
 SD 
 Naive SE 
 Time-series SE 
 Effective Size 
 HPD95Lower 
 HPD95Upper 
 
 
 
 
 lnL 
 35.2812900 
 2.6589546 
 0.0031765 
 0.0515555 
 2659.94061 
 30.1616298 
 40.9963255 
 
 
 prior 
 -17.5213760 
 4.9272709 
 0.0058863 
 0.0834153 
 3489.16662 
 -26.6309811 
 -10.1597370 
 
 
 alpha 
 2.4490592 
 2.6466302 
 0.0031617 
 0.1927261 
 188.58433 
 0.1026100 
 7.4990343 
 
 
 sigma^2 
 0.0766546 
 0.0816180 
 0.0000975 
 0.0123166 
 43.91285 
 0.0039715 
 0.2328824 
 
 
 shift number 
 1.3489990 
 1.1444863 
 0.0013672 
 0.0168757 
 4599.37818 
 0.0000000 
 3.0000000 
 
 
 N theta 
 2.3489990 
 1.1444863 
 0.0013672 
 0.0168757 
 4599.37818 
 1.0000000 
 4.0000000 
 
 
 root theta 
 1.7846413 
 0.0868189 
 0.0001037 
 0.0025226 
 1184.53069 
 1.6147808 
 1.9586763 
 
 
 root beta 
 0.6359932 
 0.0262720 
 0.0000314 
 0.0007701 
 1163.99060 
 0.5840369 
 0.6876032 
 
 
 all theta 
 1.8581881 
 0.6461108 
 NA 
 NA 
 NA 
 NA 
 NA 
 
 
 all beta 
 0.6201451 
 0.1929334 
 NA 
 NA 
 NA 
 NA 
 NA 
 
 
 
 
 
 Shifts 
No shifts were detected with a posterior probability above the cutoff.
 
 OU parameters 
 
 
 
  
 chain1 
 chain2 
 combined chains 
 
 
 
 
 alpha 
 2.4104040 
 2.6838008 
 2.4490592 
 
 
 sigma squared 
 0.0759462 
 0.0840199 
 0.0766546 
 
 
 stationary variance 
 0.0157538 
 0.0156532 
 0.0156498 
 
 
 phylogenetic halflife 
 0.2875647 
 0.2582707 
 0.2830259 
 
 
 phylogenetic half life in units of tree height 
 0.0039391 
 0.0035378 
 0.0038769 
 
 
 
 
 
 BM 
 
 Convergence 
   
 Plots show Gelman’s R statistics for log likelihood,  \(\alpha\) , and  \(\sigma^2\) . Solid lines indicate values Gelman’s R, and dashed lines indicate 95% CIs. 
 
 
 Likelihood 
   
 Plot shows the posterior probabilities of a shift occuring on each branch for each chain prior to merging. 
 
 
 Summary Statistics 
 
 chain1 
 
 
  
 Mean 
 SD 
 Naive SE 
 Time-series SE 
 Effective Size 
 HPD95Lower 
 HPD95Upper 
 
 
 
 
 lnL 
 18.2775948 
 7.4803682 
 0.0105735 
 0.5631936 
 176.4130 
 15.7843822 
 20.4164477 
 
 
 prior 
 0.6164713 
 0.2044164 
 0.0002889 
 0.0112881 
 327.9374 
 0.4440760 
 0.7061801 
 
 
 alpha 
 0.0000010 
 0.0000000 
 0.0000000 
 0.0000000 
 0.0000 
 0.0000010 
 0.0000010 
 
 
 sigma^2 
 0.0032838 
 0.0505091 
 0.0000714 
 0.0020181 
 626.3865 
 0.0008291 
 0.0019532 
 
 
 shift number 
 0.0000000 
 0.0000000 
 0.0000000 
 0.0000000 
 0.0000 
 0.0000000 
 0.0000000 
 
 
 N theta 
 1.0000000 
 0.0000000 
 0.0000000 
 0.0000000 
 0.0000 
 1.0000000 
 1.0000000 
 
 
 root theta 
 2.0113992 
 0.2309762 
 0.0003265 
 0.0119320 
 374.7224 
 1.5601547 
 2.4697285 
 
 
 root beta 
 0.5573060 
 0.0603019 
 0.0000852 
 0.0020121 
 898.1489 
 0.4406410 
 0.6764485 
 
 
 all theta 
 2.0113992 
 0.2309762 
 NA 
 NA 
 NA 
 NA 
 NA 
 
 
 all beta 
 0.5573060 
 0.0603019 
 NA 
 NA 
 NA 
 NA 
 NA 
 
 
 
 
 chain2 
 
 
  
 Mean 
 SD 
 Naive SE 
 Time-series SE 
 Effective Size 
 HPD95Lower 
 HPD95Upper 
 
 
 
 
 lnL 
 18.3064079 
 7.3184538 
 0.0103447 
 0.5598556 
 170.8782 
 16.0688213 
 20.4153472 
 
 
 prior 
 0.6237749 
 0.1850790 
 0.0002616 
 0.0096982 
 364.1915 
 0.4663646 
 0.7061923 
 
 
 alpha 
 0.0000010 
 0.0000000 
 0.0000000 
 0.0000000 
 0.0000 
 0.0000010 
 0.0000010 
 
 
 sigma^2 
 0.0029100 
 0.0376909 
 0.0000533 
 0.0013298 
 803.3977 
 0.0008499 
 0.0019774 
 
 
 shift number 
 0.0000000 
 0.0000000 
 0.0000000 
 0.0000000 
 0.0000 
 0.0000000 
 0.0000000 
 
 
 N theta 
 1.0000000 
 0.0000000 
 0.0000000 
 0.0000000 
 0.0000 
 1.0000000 
 1.0000000 
 
 
 root theta 
 2.0012299 
 0.2160071 
 0.0003053 
 0.0104336 
 428.6126 
 1.5771577 
 2.4281743 
 
 
 root beta 
 0.5607403 
 0.0609605 
 0.0000862 
 0.0020502 
 884.0677 
 0.4467587 
 0.6751603 
 
 
 all theta 
 2.0012299 
 0.2160071 
 NA 
 NA 
 NA 
 NA 
 NA 
 
 
 all beta 
 0.5607403 
 0.0609605 
 NA 
 NA 
 NA 
 NA 
 NA 
 
 
 
 
 combined chains 
 
 
  
 Mean 
 SD 
 Naive SE 
 Time-series SE 
 Effective Size 
 HPD95Lower 
 HPD95Upper 
 
 
 
 
 lnL 
 18.7783360 
 1.3500690 
 0.0016128 
 0.0278327 
 2352.8896 
 16.1364436 
 20.4158315 
 
 
 prior 
 0.6284968 
 0.0834715 
 0.0000997 
 0.0032951 
 641.6917 
 0.4629641 
 0.7061923 
 
 
 alpha 
 0.0000010 
 0.0000000 
 0.0000000 
 0.0000000 
 0.0000 
 0.0000010 
 0.0000010 
 
 
 sigma^2 
 0.0013603 
 0.0002890 
 0.0000003 
 0.0000090 
 1035.1674 
 0.0008578 
 0.0019345 
 
 
 shift number 
 0.0000000 
 0.0000000 
 0.0000000 
 0.0000000 
 0.0000 
 0.0000000 
 0.0000000 
 
 
 N theta 
 1.0000000 
 0.0000000 
 0.0000000 
 0.0000000 
 0.0000 
 1.0000000 
 1.0000000 
 
 
 root theta 
 2.0101932 
 0.2202032 
 0.0002631 
 0.0091532 
 578.7599 
 1.5771642 
 2.4407832 
 
 
 root beta 
 0.5584174 
 0.0584850 
 0.0000699 
 0.0015991 
 1337.5705 
 0.4467370 
 0.6754541 
 
 
 all theta 
 2.0101932 
 0.2202032 
 NA 
 NA 
 NA 
 NA 
 NA 
 
 
 all beta 
 0.5584174 
 0.0584850 
 NA 
 NA 
 NA 
 NA 
 NA 
 
 
 
 
 
 Shifts 
No shifts were detected with a posterior probability above the cutoff.
 
 OU parameters 
 
 
 
  
 chain1 
 chain2 
 combined chains 
 
 
 
 
 alpha 
 1.000000e-06 
 1.000000e-06 
 1.000000e-06 
 
 
 sigma squared 
 3.283800e-03 
 2.910000e-03 
 1.360300e-03 
 
 
 stationary variance 
 1.641894e+03 
 1.455002e+03 
 6.801678e+02 
 
 
 phylogenetic halflife 
 6.931472e+05 
 6.931472e+05 
 6.931472e+05 
 
 
 phylogenetic half life in units of tree height 
 9.494774e+03 
 9.494774e+03 
 9.494774e+03 
 
 
 
 
 
 Model Selection 
 
 Bayes Factors 
 
 
  
 OU un-weighted predictor 
 OU weighted predictor 
 BM 
 
 
 
 
 OU un-weighted predictor 
 0.0000000 
 0.2036888 
 -13.20949 
 
 
 OU weighted predictor 
 -0.2036888 
 0.0000000 
 -13.41318 
 
 
 BM 
 13.2094895 
 13.4131783 
 0.00000 
 
 
 
 
 
 
 Neocortex ~ Body Mass 
 
 OU Un-weighted Predictor 
 
 Convergence 
   
 Plots show Gelman’s R statistics for log likelihood,  \(\alpha\) , and  \(\sigma^2\) . Solid lines indicate values Gelman’s R, and dashed lines indicate 95% CIs. 
 
 
 Likelihood 
   
 Plot shows the posterior probabilities of a shift occuring on each branch for each chain prior to merging. 
 
 
 Summary Statistics 
 
 chain1 
 
 
  
 Mean 
 SD 
 Naive SE 
 Time-series SE 
 Effective Size 
 HPD95Lower 
 HPD95Upper 
 
 
 
 
 lnL 
 32.8168851 
 325.3523158 
 0.4598873 
 2.4571315 
 17532.7883 
 23.2103516 
 45.1411171 
 
 
 prior 
 -24.4030877 
 6.2068539 
 0.0087734 
 0.1341944 
 2139.3137 
 -35.5309484 
 -13.6087716 
 
 
 alpha 
 0.1716046 
 0.8369939 
 0.0011831 
 0.0578385 
 209.4166 
 0.0000044 
 0.8436107 
 
 
 sigma^2 
 0.0084372 
 0.0391459 
 0.0000553 
 0.0028598 
 187.3763 
 0.0003062 
 0.0400217 
 
 
 shift number 
 3.5202887 
 1.5232459 
 0.0021531 
 0.0325417 
 2191.0895 
 1.0000000 
 6.0000000 
 
 
 N theta 
 4.5202887 
 1.5232459 
 0.0021531 
 0.0325417 
 2191.0895 
 2.0000000 
 7.0000000 
 
 
 root theta 
 1.6313836 
 0.1631586 
 0.0002306 
 0.0074549 
 479.0057 
 1.3181201 
 1.9548490 
 
 
 root beta 
 0.7629408 
 0.0503600 
 0.0000712 
 0.0024286 
 429.9871 
 0.6575935 
 0.8571897 
 
 
 all theta 
 1.3251069 
 0.8957646 
 NA 
 NA 
 NA 
 NA 
 NA 
 
 
 all beta 
 0.7960448 
 0.1136990 
 NA 
 NA 
 NA 
 NA 
 NA 
 
 
 
 
 chain2 
 
 
  
 Mean 
 SD 
 Naive SE 
 Time-series SE 
 Effective Size 
 HPD95Lower 
 HPD95Upper 
 
 
 
 
 lnL 
 34.7617046 
 7.5023820 
 0.0106047 
 0.1727186 
 1886.7743 
 22.2552417 
 45.0002571 
 
 
 prior 
 -24.5015083 
 6.3791129 
 0.0090169 
 0.1516194 
 1770.1548 
 -36.1846758 
 -13.6001526 
 
 
 alpha 
 0.3187654 
 1.1402167 
 0.0016117 
 0.0651448 
 306.3484 
 0.0000040 
 1.9669025 
 
 
 sigma^2 
 0.0129646 
 0.0430854 
 0.0000609 
 0.0026391 
 266.5399 
 0.0002867 
 0.0779887 
 
 
 shift number 
 3.5402487 
 1.5478234 
 0.0021879 
 0.0354216 
 1909.4418 
 1.0000000 
 6.0000000 
 
 
 N theta 
 4.5402487 
 1.5478234 
 0.0021879 
 0.0354216 
 1909.4418 
 2.0000000 
 7.0000000 
 
 
 root theta 
 1.6256985 
 0.1594735 
 0.0002254 
 0.0070665 
 509.2962 
 1.3082366 
 1.9456518 
 
 
 root beta 
 0.7663215 
 0.0528718 
 0.0000747 
 0.0028236 
 350.6163 
 0.6648005 
 0.8696241 
 
 
 all theta 
 1.4345007 
 0.8570816 
 NA 
 NA 
 NA 
 NA 
 NA 
 
 
 all beta 
 0.7966503 
 0.1280298 
 NA 
 NA 
 NA 
 NA 
 NA 
 
 
 
 
 combined chains 
 
 
  
 Mean 
 SD 
 Naive SE 
 Time-series SE 
 Effective Size 
 HPD95Lower 
 HPD95Upper 
 
 
 
 
 lnL 
 35.9948247 
 4.7117431 
 0.0056288 
 0.1297377 
 1318.9570 
 27.2722514 
 45.8533264 
 
 
 prior 
 -24.5317310 
 6.1340891 
 0.0073280 
 0.1147180 
 2859.1482 
 -35.6316553 
 -13.6113687 
 
 
 alpha 
 0.0098306 
 0.0164825 
 0.0000197 
 0.0007413 
 494.4096 
 0.0000044 
 0.0264806 
 
 
 sigma^2 
 0.0009273 
 0.0006842 
 0.0000008 
 0.0000256 
 715.2035 
 0.0003999 
 0.0015633 
 
 
 shift number 
 3.5703093 
 1.5090296 
 0.0018027 
 0.0277791 
 2950.9401 
 1.0000000 
 6.0000000 
 
 
 N theta 
 4.5703093 
 1.5090296 
 0.0018027 
 0.0277791 
 2950.9401 
 2.0000000 
 7.0000000 
 
 
 root theta 
 1.6290920 
 0.1649816 
 0.0001971 
 0.0065984 
 625.1631 
 1.3070906 
 1.9535991 
 
 
 root beta 
 0.7646730 
 0.0490338 
 0.0000586 
 0.0025157 
 379.8992 
 0.6680403 
 0.8593100 
 
 
 all theta 
 1.3535442 
 0.9228891 
 NA 
 NA 
 NA 
 NA 
 NA 
 
 
 all beta 
 0.7934831 
 0.1016272 
 NA 
 NA 
 NA 
 NA 
 NA 
 
 
 
 
 
 Shifts 
      
 A shows the locations of the selection regimes on the phylogeny. B shows the optimum regression lines associated with each regime. C and D show the posterior distribution of  \(\beta\)  and  \(\theta\)  values associated with each regime. 
   
 Plot shows the median values for parameters associated with each selection regime, along with the posterior probability associeated with each regime.  \(\theta\)  is the intercept of the optimum regression line, and  \(\beta\)  is the coefficient of the predictor. 
 
 
 
 OU parameters 
 
 
 
  
 chain1 
 chain2 
 combined chains 
 
 
 
 
 alpha 
 0.1716046 
 0.3187654 
 0.0098306 
 
 
 sigma squared 
 0.0084372 
 0.0129646 
 0.0009273 
 
 
 stationary variance 
 0.0245834 
 0.0203356 
 0.0471645 
 
 
 phylogenetic halflife 
 4.0392108 
 2.1744741 
 70.5089047 
 
 
 phylogenetic half life in units of tree height 
 0.0553294 
 0.0297861 
 0.9658355 
 
 
 
 
 
 OU Weighted Predictor 
 
 Convergence 
   
 Plots show Gelman’s R statistics for log likelihood,  \(\alpha\) , and  \(\sigma^2\) . Solid lines indicate values Gelman’s R, and dashed lines indicate 95% CIs. 
 
 
 Likelihood 
   
 Plot shows the posterior probabilities of a shift occuring on each branch for each chain prior to merging. 
 
 
 Summary Statistics 
 
 chain1 
 
 
  
 Mean 
 SD 
 Naive SE 
 Time-series SE 
 Effective Size 
 HPD95Lower 
 HPD95Upper 
 
 
 
 
 lnL 
 27.7299483 
 7.2727090 
 0.0102800 
 0.3319691 
 479.95107 
 18.1191079 
 45.5480671 
 
 
 prior 
 -25.5238969 
 7.2465200 
 0.0102430 
 0.2043728 
 1257.22388 
 -39.1606209 
 -13.7117725 
 
 
 alpha 
 1.9745880 
 1.2575676 
 0.0017776 
 0.0639144 
 387.13761 
 0.1682837 
 4.1813244 
 
 
 sigma^2 
 0.0791454 
 0.0386295 
 0.0000546 
 0.0095792 
 16.26233 
 0.0101921 
 0.1471391 
 
 
 shift number 
 3.5076134 
 1.7655332 
 0.0024956 
 0.0484508 
 1327.85008 
 1.0000000 
 7.0000000 
 
 
 N theta 
 4.5076134 
 1.7655332 
 0.0024956 
 0.0484508 
 1327.85008 
 2.0000000 
 8.0000000 
 
 
 root theta 
 1.5983688 
 0.1599716 
 0.0002261 
 0.0058570 
 746.00240 
 1.3091754 
 1.9102546 
 
 
 root beta 
 0.7810255 
 0.0784384 
 0.0001109 
 0.0027180 
 832.83177 
 0.6404256 
 0.9096880 
 
 
 all theta 
 1.5037504 
 0.6343943 
 NA 
 NA 
 NA 
 NA 
 NA 
 
 
 all beta 
 0.8314348 
 0.2018003 
 NA 
 NA 
 NA 
 NA 
 NA 
 
 
 
 
 chain2 
 
 
  
 Mean 
 SD 
 Naive SE 
 Time-series SE 
 Effective Size 
 HPD95Lower 
 HPD95Upper 
 
 
 
 
 lnL 
 26.8093017 
 25.7970518 
 0.0364643 
 0.2287368 
 12719.44549 
 17.4827060 
 42.9254696 
 
 
 prior 
 -25.0632333 
 6.9830689 
 0.0098706 
 0.1805274 
 1496.25721 
 -38.4131284 
 -14.0243474 
 
 
 alpha 
 1.9492654 
 1.3485050 
 0.0019061 
 0.0738196 
 333.70466 
 0.4129059 
 4.8562957 
 
 
 sigma^2 
 0.0813729 
 0.0533122 
 0.0000754 
 0.0167145 
 10.17336 
 0.0241168 
 0.2133917 
 
 
 shift number 
 3.3965606 
 1.7061457 
 0.0024116 
 0.0427553 
 1592.40296 
 1.0000000 
 7.0000000 
 
 
 N theta 
 4.3965606 
 1.7061457 
 0.0024116 
 0.0427553 
 1592.40296 
 2.0000000 
 8.0000000 
 
 
 root theta 
 1.5829906 
 0.1601966 
 0.0002264 
 0.0063977 
 626.99035 
 1.2700234 
 1.8832959 
 
 
 root beta 
 0.7873342 
 0.0779077 
 0.0001101 
 0.0028171 
 764.81433 
 0.6397492 
 0.9140903 
 
 
 all theta 
 1.4860431 
 0.6366793 
 NA 
 NA 
 NA 
 NA 
 NA 
 
 
 all beta 
 0.8357730 
 0.2032378 
 NA 
 NA 
 NA 
 NA 
 NA 
 
 
 
 
 combined chains 
 
 
  
 Mean 
 SD 
 Naive SE 
 Time-series SE 
 Effective Size 
 HPD95Lower 
 HPD95Upper 
 
 
 
 
 lnL 
 27.4931909 
 6.7523610 
 0.0080666 
 0.2651818 
 648.37136 
 17.8932531 
 44.5848315 
 
 
 prior 
 -25.1563465 
 7.0231426 
 0.0083900 
 0.1587772 
 1956.53108 
 -38.3626798 
 -13.7289268 
 
 
 alpha 
 1.7700885 
 1.1364938 
 0.0013577 
 0.0498612 
 519.52864 
 0.1944426 
 3.9083460 
 
 
 sigma^2 
 0.0722819 
 0.0407478 
 0.0000487 
 0.0092439 
 19.43098 
 0.0101921 
 0.1513429 
 
 
 shift number 
 3.4719725 
 1.7267705 
 0.0020628 
 0.0385442 
 2007.01300 
 1.0000000 
 7.0000000 
 
 
 N theta 
 4.4719725 
 1.7267705 
 0.0020628 
 0.0385442 
 2007.01300 
 2.0000000 
 8.0000000 
 
 
 root theta 
 1.5883577 
 0.1578281 
 0.0001885 
 0.0051290 
 946.88858 
 1.2879207 
 1.8932525 
 
 
 root beta 
 0.7856376 
 0.0772600 
 0.0000923 
 0.0023906 
 1044.49521 
 0.6438340 
 0.9126602 
 
 
 all theta 
 1.4912459 
 0.6334104 
 NA 
 NA 
 NA 
 NA 
 NA 
 
 
 all beta 
 0.8340280 
 0.2015946 
 NA 
 NA 
 NA 
 NA 
 NA 
 
 
 
 
 
 Shifts 
      
 A shows the locations of the selection regimes on the phylogeny. B shows the optimum regression lines associated with each regime. C and D show the posterior distribution of  \(\beta\)  and  \(\theta\)  values associated with each regime. 
   
 Plot shows the median values for parameters associated with each selection regime, along with the posterior probability associeated with each regime.  \(\theta\)  is the intercept of the optimum regression line, and  \(\beta\)  is the coefficient of the predictor. 
 
 
 
 OU parameters 
 
 
 
  
 chain1 
 chain2 
 combined chains 
 
 
 
 
 alpha 
 1.9745880 
 1.9492654 
 1.7700885 
 
 
 sigma squared 
 0.0791454 
 0.0813729 
 0.0722819 
 
 
 stationary variance 
 0.0200410 
 0.0208727 
 0.0204176 
 
 
 phylogenetic halflife 
 0.3510338 
 0.3555941 
 0.3915890 
 
 
 phylogenetic half life in units of tree height 
 0.0048085 
 0.0048709 
 0.0053640 
 
 
 
 
 
 BM 
 
 Convergence 
   
 Plots show Gelman’s R statistics for log likelihood,  \(\alpha\) , and  \(\sigma^2\) . Solid lines indicate values Gelman’s R, and dashed lines indicate 95% CIs. 
 
 
 Likelihood 
   
 Plot shows the posterior probabilities of a shift occuring on each branch for each chain prior to merging. 
 
 
 Summary Statistics 
 
 chain1 
 
 
  
 Mean 
 SD 
 Naive SE 
 Time-series SE 
 Effective Size 
 HPD95Lower 
 HPD95Upper 
 
 
 
 
 lnL 
 20.8715295 
 5.8345207 
 0.0082471 
 0.3297257 
 313.1157 
 18.3840937 
 22.8589064 
 
 
 prior 
 0.5385952 
 0.1854439 
 0.0002621 
 0.0086007 
 464.8978 
 0.2897727 
 0.7061340 
 
 
 alpha 
 0.0000010 
 0.0000000 
 0.0000000 
 0.0000000 
 0.0000 
 0.0000010 
 0.0000010 
 
 
 sigma^2 
 0.0023161 
 0.0334421 
 0.0000473 
 0.0009781 
 1169.0522 
 0.0007949 
 0.0017988 
 
 
 shift number 
 0.0000000 
 0.0000000 
 0.0000000 
 0.0000000 
 0.0000 
 0.0000000 
 0.0000000 
 
 
 N theta 
 1.0000000 
 0.0000000 
 0.0000000 
 0.0000000 
 0.0000 
 1.0000000 
 1.0000000 
 
 
 root theta 
 1.7390091 
 0.2242857 
 0.0003170 
 0.0117307 
 365.5590 
 1.3267016 
 2.1903187 
 
 
 root beta 
 0.7418696 
 0.0601616 
 0.0000850 
 0.0020899 
 828.6437 
 0.6237639 
 0.8507326 
 
 
 all theta 
 1.7390091 
 0.2242857 
 NA 
 NA 
 NA 
 NA 
 NA 
 
 
 all beta 
 0.7418696 
 0.0601616 
 NA 
 NA 
 NA 
 NA 
 NA 
 
 
 
 
 chain2 
 
 
  
 Mean 
 SD 
 Naive SE 
 Time-series SE 
 Effective Size 
 HPD95Lower 
 HPD95Upper 
 
 
 
 
 lnL 
 21.0961872 
 5.8271612 
 0.0082367 
 0.3416098 
 290.9734 
 18.9708934 
 22.8607812 
 
 
 prior 
 0.5374127 
 0.1708326 
 0.0002415 
 0.0076535 
 498.2259 
 0.3308981 
 0.7057571 
 
 
 alpha 
 0.0000010 
 0.0000000 
 0.0000000 
 0.0000000 
 0.0000 
 0.0000010 
 0.0000010 
 
 
 sigma^2 
 0.0021657 
 0.0289035 
 0.0000409 
 0.0009220 
 982.7194 
 0.0007761 
 0.0017194 
 
 
 shift number 
 0.0000000 
 0.0000000 
 0.0000000 
 0.0000000 
 0.0000 
 0.0000000 
 0.0000000 
 
 
 N theta 
 1.0000000 
 0.0000000 
 0.0000000 
 0.0000000 
 0.0000 
 1.0000000 
 1.0000000 
 
 
 root theta 
 1.7551085 
 0.1884625 
 0.0002664 
 0.0083070 
 514.7030 
 1.3846736 
 2.1215177 
 
 
 root beta 
 0.7354261 
 0.0512223 
 0.0000724 
 0.0015023 
 1162.4672 
 0.6354001 
 0.8339267 
 
 
 all theta 
 1.7551085 
 0.1884625 
 NA 
 NA 
 NA 
 NA 
 NA 
 
 
 all beta 
 0.7354261 
 0.0512223 
 NA 
 NA 
 NA 
 NA 
 NA 
 
 
 
 
 combined chains 
 
 
  
 Mean 
 SD 
 Naive SE 
 Time-series SE 
 Effective Size 
 HPD95Lower 
 HPD95Upper 
 
 
 
 
 lnL 
 21.2581897 
 1.3037861 
 0.0015575 
 0.0242721 
 2885.3370 
 18.6966750 
 22.8616275 
 
 
 prior 
 0.5448964 
 0.1197458 
 0.0001431 
 0.0049407 
 587.4213 
 0.3132632 
 0.7061011 
 
 
 alpha 
 0.0000010 
 0.0000000 
 0.0000000 
 0.0000000 
 0.0000 
 0.0000010 
 0.0000010 
 
 
 sigma^2 
 0.0012331 
 0.0002535 
 0.0000003 
 0.0000060 
 1807.7957 
 0.0007878 
 0.0017434 
 
 
 shift number 
 0.0000000 
 0.0000000 
 0.0000000 
 0.0000000 
 0.0000 
 0.0000000 
 0.0000000 
 
 
 N theta 
 1.0000000 
 0.0000000 
 0.0000000 
 0.0000000 
 0.0000 
 1.0000000 
 1.0000000 
 
 
 root theta 
 1.7437475 
 0.2079345 
 0.0002484 
 0.0086983 
 571.4539 
 1.3434474 
 2.1630782 
 
 
 root beta 
 0.7393104 
 0.0548553 
 0.0000655 
 0.0014734 
 1386.1299 
 0.6306284 
 0.8450501 
 
 
 all theta 
 1.7437475 
 0.2079345 
 NA 
 NA 
 NA 
 NA 
 NA 
 
 
 all beta 
 0.7393104 
 0.0548553 
 NA 
 NA 
 NA 
 NA 
 NA 
 
 
 
 
 
 Shifts 
No shifts were detected with a posterior probability above the cutoff.
 
 OU parameters 
 
 
 
  
 chain1 
 chain2 
 combined chains 
 
 
 
 
 alpha 
 1.000000e-06 
 1.000000e-06 
 1.000000e-06 
 
 
 sigma squared 
 2.316100e-03 
 2.165700e-03 
 1.233100e-03 
 
 
 stationary variance 
 1.158037e+03 
 1.082865e+03 
 6.165456e+02 
 
 
 phylogenetic halflife 
 6.931472e+05 
 6.931472e+05 
 6.931472e+05 
 
 
 phylogenetic half life in units of tree height 
 9.494774e+03 
 9.494774e+03 
 9.494774e+03 
 
 
 
 
 
 Model Selection 
 
 Bayes Factors 
 
 
  
 OU un-weighted predictor 
 OU weighted predictor 
 BM 
 
 
 
 
 OU un-weighted predictor 
 0.000000 
 -3.995941 
 18.21388 
 
 
 OU weighted predictor 
 3.995941 
 0.000000 
 22.20983 
 
 
 BM 
 -18.213884 
 -22.209825 
 0.00000 
 
 
 
 
 
 
 Neocortex ~ Rest of Brain 
 
 OU Un-weighted Predictor 
 
 Convergence 
   
 Plots show Gelman’s R statistics for log likelihood,  \(\alpha\) , and  \(\sigma^2\) . Solid lines indicate values Gelman’s R, and dashed lines indicate 95% CIs. 
 
 
 Likelihood 
   
 Plot shows the posterior probabilities of a shift occuring on each branch for each chain prior to merging. 
 
 
 Summary Statistics 
 
 chain1 
 
 
  
 Mean 
 SD 
 Naive SE 
 Time-series SE 
 Effective Size 
 HPD95Lower 
 HPD95Upper 
 
 
 
 
 lnL 
 41.8484663 
 968.1801624 
 1.3685281 
 2.5734017 
 141545.86190 
 35.7727162 
 51.5812024 
 
 
 prior 
 -23.2532256 
 4.5627496 
 0.0064495 
 0.1013316 
 2027.51208 
 -31.5383428 
 -13.8553269 
 
 
 alpha 
 2.1636622 
 1.7379749 
 0.0024566 
 0.1382129 
 158.12120 
 0.0062162 
 5.6566696 
 
 
 sigma^2 
 0.0472127 
 0.0363335 
 0.0000514 
 0.0098932 
 13.48773 
 0.0010836 
 0.1276905 
 
 
 shift number 
 2.9008673 
 1.0744340 
 0.0015187 
 0.0220908 
 2365.57596 
 1.0000000 
 5.0000000 
 
 
 N theta 
 3.9008673 
 1.0744340 
 0.0015187 
 0.0220908 
 2365.57596 
 2.0000000 
 6.0000000 
 
 
 root theta 
 -0.6339715 
 0.2108443 
 0.0002980 
 0.0083834 
 632.53131 
 -1.0156088 
 -0.2434912 
 
 
 root beta 
 1.2418326 
 0.0814384 
 0.0001151 
 0.0025560 
 1015.15219 
 1.1179615 
 1.3615942 
 
 
 all theta 
 -0.7405324 
 0.6921804 
 NA 
 NA 
 NA 
 NA 
 NA 
 
 
 all beta 
 1.2713242 
 0.1649926 
 NA 
 NA 
 NA 
 NA 
 NA 
 
 
 
 
 chain2 
 
 
  
 Mean 
 SD 
 Naive SE 
 Time-series SE 
 Effective Size 
 HPD95Lower 
 HPD95Upper 
 
 
 
 
 lnL 
 43.8279981 
 6.5804940 
 0.0093016 
 0.1134002 
 3367.35852 
 35.5612005 
 51.2919730 
 
 
 prior 
 -23.7055845 
 4.5388822 
 0.0064157 
 0.0925822 
 2403.49505 
 -32.5613466 
 -15.0578766 
 
 
 alpha 
 3.3374555 
 1.8237175 
 0.0025778 
 0.1051125 
 301.02788 
 0.4217073 
 6.7914446 
 
 
 sigma^2 
 0.0754616 
 0.0370812 
 0.0000524 
 0.0065471 
 32.07837 
 0.0096908 
 0.1453442 
 
 
 shift number 
 2.7738666 
 1.0584480 
 0.0014961 
 0.0201765 
 2752.00582 
 1.0000000 
 5.0000000 
 
 
 N theta 
 3.7738666 
 1.0584480 
 0.0014961 
 0.0201765 
 2752.00582 
 2.0000000 
 6.0000000 
 
 
 root theta 
 -0.6552091 
 0.1876256 
 0.0002652 
 0.0159671 
 138.08124 
 -1.0318872 
 -0.2518178 
 
 
 root beta 
 1.2564397 
 0.0626718 
 0.0000886 
 0.0062666 
 100.01786 
 1.1307464 
 1.3691711 
 
 
 all theta 
 -0.8135768 
 0.6943325 
 NA 
 NA 
 NA 
 NA 
 NA 
 
 
 all beta 
 1.2883986 
 0.1660847 
 NA 
 NA 
 NA 
 NA 
 NA 
 
 
 
 
 combined chains 
 
 
  
 Mean 
 SD 
 Naive SE 
 Time-series SE 
 Effective Size 
 HPD95Lower 
 HPD95Upper 
 
 
 
 
 lnL 
 44.2063127 
 3.9656266 
 0.0047374 
 0.1018626 
 1515.63351 
 35.8230725 
 51.3585512 
 
 
 prior 
 -23.5228093 
 4.4778750 
 0.0053494 
 0.0810203 
 3054.61546 
 -32.4301834 
 -14.9626555 
 
 
 alpha 
 2.8489993 
 1.6936491 
 0.0020233 
 0.0919749 
 339.08457 
 0.2918139 
 6.1444828 
 
 
 sigma^2 
 0.0633741 
 0.0349860 
 0.0000418 
 0.0061284 
 32.59057 
 0.0079628 
 0.1317232 
 
 
 shift number 
 2.8171182 
 1.0398971 
 0.0012423 
 0.0172423 
 3637.38195 
 1.0000000 
 5.0000000 
 
 
 N theta 
 3.8171182 
 1.0398971 
 0.0012423 
 0.0172423 
 3637.38195 
 2.0000000 
 6.0000000 
 
 
 root theta 
 -0.6374330 
 0.1974473 
 0.0002359 
 0.0085295 
 535.86534 
 -1.0119052 
 -0.2304020 
 
 
 root beta 
 1.2526926 
 0.0729528 
 0.0000872 
 0.0026681 
 747.60055 
 1.1146628 
 1.3610931 
 
 
 all theta 
 -0.7952536 
 0.7046166 
 NA 
 NA 
 NA 
 NA 
 NA 
 
 
 all beta 
 1.2832230 
 0.1673607 
 NA 
 NA 
 NA 
 NA 
 NA 
 
 
 
 
 
 Shifts 
      
 A shows the locations of the selection regimes on the phylogeny. B shows the optimum regression lines associated with each regime. C and D show the posterior distribution of  \(\beta\)  and  \(\theta\)  values associated with each regime. 
   
 Plot shows the median values for parameters associated with each selection regime, along with the posterior probability associeated with each regime.  \(\theta\)  is the intercept of the optimum regression line, and  \(\beta\)  is the coefficient of the predictor. 
 
 
 
 OU parameters 
 
 
 
  
 chain1 
 chain2 
 combined chains 
 
 
 
 
 alpha 
 2.1636622 
 3.3374555 
 2.8489993 
 
 
 sigma squared 
 0.0472127 
 0.0754616 
 0.0633741 
 
 
 stationary variance 
 0.0109104 
 0.0113053 
 0.0111222 
 
 
 phylogenetic halflife 
 0.3203583 
 0.2076873 
 0.2432950 
 
 
 phylogenetic half life in units of tree height 
 0.0043883 
 0.0028449 
 0.0033327 
 
 
 
 
 
 OU Weighted Predictor 
 
 Convergence 
   
 Plots show Gelman’s R statistics for log likelihood,  \(\alpha\) , and  \(\sigma^2\) . Solid lines indicate values Gelman’s R, and dashed lines indicate 95% CIs. 
 
 
 Likelihood 
   
 Plot shows the posterior probabilities of a shift occuring on each branch for each chain prior to merging. 
 
 
 Summary Statistics 
 
 chain1 
 
 
  
 Mean 
 SD 
 Naive SE 
 Time-series SE 
 Effective Size 
 HPD95Lower 
 HPD95Upper 
 
 
 
 
 lnL 
 43.8979990 
 4.4951105 
 0.0063539 
 0.1397838 
 1034.11020 
 35.4665226 
 51.2292886 
 
 
 prior 
 -23.8578417 
 4.8785533 
 0.0068959 
 0.1162950 
 1759.78704 
 -32.6891924 
 -13.9740455 
 
 
 alpha 
 3.5254850 
 3.3528039 
 0.0047392 
 0.2360772 
 201.70103 
 0.2385005 
 9.6435901 
 
 
 sigma^2 
 0.0797647 
 0.0732062 
 0.0001035 
 0.0096366 
 57.70999 
 0.0058361 
 0.2125637 
 
 
 shift number 
 2.8162541 
 1.0660587 
 0.0015069 
 0.0216442 
 2425.93778 
 1.0000000 
 5.0000000 
 
 
 N theta 
 3.8162541 
 1.0660587 
 0.0015069 
 0.0216442 
 2425.93778 
 2.0000000 
 6.0000000 
 
 
 root theta 
 -0.6657844 
 0.2274737 
 0.0003215 
 0.0088728 
 657.26922 
 -1.0893730 
 -0.1897840 
 
 
 root beta 
 1.2472358 
 0.0712471 
 0.0001007 
 0.0028328 
 632.57222 
 1.0979957 
 1.3728979 
 
 
 all theta 
 -0.7906672 
 0.7157514 
 NA 
 NA 
 NA 
 NA 
 NA 
 
 
 all beta 
 1.2814128 
 0.1727140 
 NA 
 NA 
 NA 
 NA 
 NA 
 
 
 
 
 chain2 
 
 
  
 Mean 
 SD 
 Naive SE 
 Time-series SE 
 Effective Size 
 HPD95Lower 
 HPD95Upper 
 
 
 
 
 lnL 
 39.8941116 
 1060.6268466 
 1.4992020 
 4.2083819 
 63517.72602 
 35.5189152 
 51.6070603 
 
 
 prior 
 -23.5907037 
 4.7007118 
 0.0066445 
 0.0999258 
 2212.95041 
 -32.0702708 
 -14.0855715 
 
 
 alpha 
 3.0880861 
 2.9184596 
 0.0041253 
 0.2036243 
 205.42261 
 0.1653919 
 8.6588482 
 
 
 sigma^2 
 0.0698243 
 0.0635938 
 0.0000899 
 0.0083592 
 57.87616 
 0.0037153 
 0.1950417 
 
 
 shift number 
 2.8634029 
 1.0683404 
 0.0015101 
 0.0201095 
 2822.37812 
 1.0000000 
 5.0000000 
 
 
 N theta 
 3.8634029 
 1.0683404 
 0.0015101 
 0.0201095 
 2822.37812 
 2.0000000 
 6.0000000 
 
 
 root theta 
 -0.6804767 
 0.2052922 
 0.0002902 
 0.0178454 
 132.33971 
 -1.0563787 
 -0.2436789 
 
 
 root beta 
 1.2460685 
 0.0660245 
 0.0000933 
 0.0058992 
 125.26233 
 1.1226679 
 1.3698521 
 
 
 all theta 
 -0.8217033 
 0.6744663 
 NA 
 NA 
 NA 
 NA 
 NA 
 
 
 all beta 
 1.2899395 
 0.1654361 
 NA 
 NA 
 NA 
 NA 
 NA 
 
 
 
 
 combined chains 
 
 
  
 Mean 
 SD 
 Naive SE 
 Time-series SE 
 Effective Size 
 HPD95Lower 
 HPD95Upper 
 
 
 
 
 lnL 
 44.1407053 
 4.0499875 
 0.0048382 
 0.1062974 
 1451.6504 
 35.7025606 
 51.4354126 
 
 
 prior 
 -23.7346579 
 4.7996548 
 0.0057338 
 0.0949231 
 2556.6780 
 -32.4161028 
 -13.9690050 
 
 
 alpha 
 3.3856772 
 3.4595838 
 0.0041329 
 0.2157652 
 257.0898 
 0.1605903 
 9.9835032 
 
 
 sigma^2 
 0.0761431 
 0.0742844 
 0.0000887 
 0.0086867 
 73.1277 
 0.0037153 
 0.2226821 
 
 
 shift number 
 2.8347433 
 1.0601508 
 0.0012665 
 0.0179683 
 3481.1499 
 1.0000000 
 5.0000000 
 
 
 N theta 
 3.8347433 
 1.0601508 
 0.0012665 
 0.0179683 
 3481.1499 
 2.0000000 
 6.0000000 
 
 
 root theta 
 -0.6680813 
 0.2048490 
 0.0002447 
 0.0091215 
 504.3520 
 -1.0502625 
 -0.2483801 
 
 
 root beta 
 1.2471524 
 0.0672071 
 0.0000803 
 0.0031225 
 463.2499 
 1.1173492 
 1.3699408 
 
 
 all theta 
 -0.7952818 
 0.7000289 
 NA 
 NA 
 NA 
 NA 
 NA 
 
 
 all beta 
 1.2827302 
 0.1707245 
 NA 
 NA 
 NA 
 NA 
 NA 
 
 
 
 
 
 Shifts 
      
 A shows the locations of the selection regimes on the phylogeny. B shows the optimum regression lines associated with each regime. C and D show the posterior distribution of  \(\beta\)  and  \(\theta\)  values associated with each regime. 
   
 Plot shows the median values for parameters associated with each selection regime, along with the posterior probability associeated with each regime.  \(\theta\)  is the intercept of the optimum regression line, and  \(\beta\)  is the coefficient of the predictor. 
 
 
 
 OU parameters 
 
 
 
  
 chain1 
 chain2 
 combined chains 
 
 
 
 
 alpha 
 3.5254850 
 3.0880861 
 3.3856772 
 
 
 sigma squared 
 0.0797647 
 0.0698243 
 0.0761431 
 
 
 stationary variance 
 0.0113126 
 0.0113054 
 0.0112449 
 
 
 phylogenetic halflife 
 0.1966104 
 0.2244585 
 0.2047293 
 
 
 phylogenetic half life in units of tree height 
 0.0026932 
 0.0030746 
 0.0028044 
 
 
 
 
 
 BM 
 
 Convergence 
   
 Plots show Gelman’s R statistics for log likelihood,  \(\alpha\) , and  \(\sigma^2\) . Solid lines indicate values Gelman’s R, and dashed lines indicate 95% CIs. 
 
 
 Likelihood 
   
 Plot shows the posterior probabilities of a shift occuring on each branch for each chain prior to merging. 
 
 
 Summary Statistics 
 
 chain1 
 
 
  
 Mean 
 SD 
 Naive SE 
 Time-series SE 
 Effective Size 
 HPD95Lower 
 HPD95Upper 
 
 
 
 
 lnL 
 17.5150011 
 3.6378668 
 0.0051421 
 0.1257875 
 836.4088 
 15.0151934 
 19.2080224 
 
 
 prior 
 0.0165669 
 0.3883399 
 0.0005489 
 0.0257268 
 227.8525 
 -0.7030907 
 0.6884311 
 
 
 alpha 
 0.0000010 
 0.0000000 
 0.0000000 
 0.0000000 
 0.0000 
 0.0000010 
 0.0000010 
 
 
 sigma^2 
 0.0018120 
 0.0205364 
 0.0000290 
 0.0004406 
 2172.3401 
 0.0009041 
 0.0020257 
 
 
 shift number 
 0.0000000 
 0.0000000 
 0.0000000 
 0.0000000 
 0.0000 
 0.0000000 
 0.0000000 
 
 
 N theta 
 1.0000000 
 0.0000000 
 0.0000000 
 0.0000000 
 0.0000 
 1.0000000 
 1.0000000 
 
 
 root theta 
 -0.0609930 
 0.3097906 
 0.0004379 
 0.0224731 
 190.0242 
 -0.6826560 
 0.5377753 
 
 
 root beta 
 1.0859743 
 0.0786945 
 0.0001112 
 0.0037377 
 443.2905 
 0.9333284 
 1.2395857 
 
 
 all theta 
 -0.0609930 
 0.3097906 
 NA 
 NA 
 NA 
 NA 
 NA 
 
 
 all beta 
 1.0859743 
 0.0786945 
 NA 
 NA 
 NA 
 NA 
 NA 
 
 
 
 
 chain2 
 
 
  
 Mean 
 SD 
 Naive SE 
 Time-series SE 
 Effective Size 
 HPD95Lower 
 HPD95Upper 
 
 
 
 
 lnL 
 17.3634726 
 3.4730740 
 0.0049092 
 0.1065983 
 1061.5174 
 14.3767024 
 19.2089078 
 
 
 prior 
 0.0313275 
 0.4147713 
 0.0005863 
 0.0287710 
 207.8292 
 -0.7075689 
 0.7059280 
 
 
 alpha 
 0.0000010 
 0.0000000 
 0.0000000 
 0.0000000 
 0.0000 
 0.0000010 
 0.0000010 
 
 
 sigma^2 
 0.0018395 
 0.0240165 
 0.0000339 
 0.0004343 
 3058.6033 
 0.0008981 
 0.0020419 
 
 
 shift number 
 0.0000000 
 0.0000000 
 0.0000000 
 0.0000000 
 0.0000 
 0.0000000 
 0.0000000 
 
 
 N theta 
 1.0000000 
 0.0000000 
 0.0000000 
 0.0000000 
 0.0000 
 1.0000000 
 1.0000000 
 
 
 root theta 
 -0.0887087 
 0.3567984 
 0.0005043 
 0.0288710 
 152.7290 
 -0.8569471 
 0.5581567 
 
 
 root beta 
 1.0925840 
 0.0892841 
 0.0001262 
 0.0048934 
 332.9064 
 0.9224502 
 1.2750628 
 
 
 all theta 
 -0.0887087 
 0.3567984 
 NA 
 NA 
 NA 
 NA 
 NA 
 
 
 all beta 
 1.0925840 
 0.0892841 
 NA 
 NA 
 NA 
 NA 
 NA 
 
 
 
 
 combined chains 
 
 
  
 Mean 
 SD 
 Naive SE 
 Time-series SE 
 Effective Size 
 HPD95Lower 
 HPD95Upper 
 
 
 
 
 lnL 
 17.6151197 
 1.3390684 
 0.0015997 
 0.0202266 
 4382.8985 
 14.9845493 
 19.2093645 
 
 
 prior 
 0.0204465 
 0.3772335 
 0.0004507 
 0.0232925 
 262.2946 
 -0.6797911 
 0.7018271 
 
 
 alpha 
 0.0000010 
 0.0000000 
 0.0000000 
 0.0000000 
 0.0000 
 0.0000010 
 0.0000010 
 
 
 sigma^2 
 0.0014139 
 0.0002979 
 0.0000004 
 0.0000032 
 8523.5930 
 0.0008871 
 0.0020107 
 
 
 shift number 
 0.0000000 
 0.0000000 
 0.0000000 
 0.0000000 
 0.0000 
 0.0000000 
 0.0000000 
 
 
 N theta 
 1.0000000 
 0.0000000 
 0.0000000 
 0.0000000 
 0.0000 
 1.0000000 
 1.0000000 
 
 
 root theta 
 -0.0620764 
 0.3078858 
 0.0003678 
 0.0187659 
 269.1786 
 -0.6691354 
 0.5533120 
 
 
 root beta 
 1.0860732 
 0.0779255 
 0.0000931 
 0.0031770 
 601.6116 
 0.9318730 
 1.2387710 
 
 
 all theta 
 -0.0620764 
 0.3078858 
 NA 
 NA 
 NA 
 NA 
 NA 
 
 
 all beta 
 1.0860732 
 0.0779255 
 NA 
 NA 
 NA 
 NA 
 NA 
 
 
 
 
 
 Shifts 
No shifts were detected with a posterior probability above the cutoff.
 
 OU parameters 
 
 
 
  
 chain1 
 chain2 
 combined chains 
 
 
 
 
 alpha 
 1.000000e-06 
 1.000000e-06 
 1.000000e-06 
 
 
 sigma squared 
 1.812000e-03 
 1.839500e-03 
 1.413900e-03 
 
 
 stationary variance 
 9.059836e+02 
 9.197657e+02 
 7.069329e+02 
 
 
 phylogenetic halflife 
 6.931472e+05 
 6.931472e+05 
 6.931472e+05 
 
 
 phylogenetic half life in units of tree height 
 9.494774e+03 
 9.494774e+03 
 9.494774e+03 
 
 
 
 
 
 Model Selection 
 
 Bayes Factors 
 
 
  
 OU un-weighted predictor 
 OU weighted predictor 
 BM 
 
 
 
 
 OU un-weighted predictor 
 0.000000 
 9.287355 
 -5.080023 
 
 
 OU weighted predictor 
 -9.287355 
 0.000000 
 -14.367379 
 
 
 BM 
 5.080023 
 14.367379 
 0.000000 
 
 
 
 
 
 
 Cerebellum ~ Body Mass 
 
 OU Un-weighted Predictor 
 
 Convergence 
   
 Plots show Gelman’s R statistics for log likelihood,  \(\alpha\) , and  \(\sigma^2\) . Solid lines indicate values Gelman’s R, and dashed lines indicate 95% CIs. 
 
 
 Likelihood 
   
 Plot shows the posterior probabilities of a shift occuring on each branch for each chain prior to merging. 
 
 
 Summary Statistics 
 
 chain1 
 
 
  
 Mean 
 SD 
 Naive SE 
 Time-series SE 
 Effective Size 
 HPD95Lower 
 HPD95Upper 
 
 
 
 
 lnL 
 36.4566292 
 5.2871738 
 0.0074734 
 0.1968457 
 721.43166 
 26.6512833 
 44.8588394 
 
 
 prior 
 -22.6710409 
 5.6442399 
 0.0079782 
 0.1343198 
 1765.75752 
 -35.4633553 
 -13.5989196 
 
 
 alpha 
 3.3967852 
 5.9438755 
 0.0084017 
 0.5952794 
 99.70060 
 0.0038035 
 9.9937069 
 
 
 sigma^2 
 0.1050424 
 0.2063346 
 0.0002917 
 0.0338398 
 37.17824 
 0.0010685 
 0.2946486 
 
 
 shift number 
 2.6200347 
 1.2740112 
 0.0018008 
 0.0261663 
 2370.62251 
 0.0000000 
 5.0000000 
 
 
 N theta 
 3.6200347 
 1.2740112 
 0.0018008 
 0.0261663 
 2370.62251 
 1.0000000 
 6.0000000 
 
 
 root theta 
 1.0382549 
 0.1167466 
 0.0001650 
 0.0061941 
 355.25057 
 0.8001524 
 1.2559433 
 
 
 root beta 
 0.7520156 
 0.0388820 
 0.0000550 
 0.0022372 
 302.06664 
 0.6811840 
 0.8329248 
 
 
 all theta 
 0.9931324 
 0.6417818 
 NA 
 NA 
 NA 
 NA 
 NA 
 
 
 all beta 
 0.7950026 
 0.2009048 
 NA 
 NA 
 NA 
 NA 
 NA 
 
 
 
 
 chain2 
 
 
  
 Mean 
 SD 
 Naive SE 
 Time-series SE 
 Effective Size 
 HPD95Lower 
 HPD95Upper 
 
 
 
 
 lnL 
 36.4622926 
 5.0899284 
 0.0071946 
 0.1642591 
 960.20555 
 27.1691024 
 44.0587734 
 
 
 prior 
 -22.9663043 
 5.6445773 
 0.0079786 
 0.1368128 
 1702.19561 
 -35.4640343 
 -13.5986330 
 
 
 alpha 
 3.9902225 
 5.2405207 
 0.0074075 
 0.5069369 
 106.86637 
 0.0019369 
 13.5508251 
 
 
 sigma^2 
 0.1188536 
 0.1542000 
 0.0002180 
 0.0398436 
 14.97788 
 0.0008494 
 0.3952444 
 
 
 shift number 
 2.5623925 
 1.2276378 
 0.0017353 
 0.0245591 
 2498.71025 
 1.0000000 
 5.0000000 
 
 
 N theta 
 3.5623925 
 1.2276378 
 0.0017353 
 0.0245591 
 2498.71025 
 2.0000000 
 6.0000000 
 
 
 root theta 
 1.0432452 
 0.1097785 
 0.0001552 
 0.0056271 
 380.59146 
 0.8192346 
 1.2560838 
 
 
 root beta 
 0.7507707 
 0.0363652 
 0.0000514 
 0.0019621 
 343.51663 
 0.6822760 
 0.8259890 
 
 
 all theta 
 1.0150288 
 0.6476237 
 NA 
 NA 
 NA 
 NA 
 NA 
 
 
 all beta 
 0.7856301 
 0.2000887 
 NA 
 NA 
 NA 
 NA 
 NA 
 
 
 
 
 combined chains 
 
 
  
 Mean 
 SD 
 Naive SE 
 Time-series SE 
 Effective Size 
 HPD95Lower 
 HPD95Upper 
 
 
 
 
 lnL 
 36.6716941 
 4.8719617 
 0.0058202 
 0.1620806 
 903.53652 
 27.0331859 
 44.7378321 
 
 
 prior 
 -22.4925545 
 5.6129220 
 0.0067054 
 0.1150156 
 2381.57458 
 -35.6923940 
 -13.5974380 
 
 
 alpha 
 3.0593154 
 4.3930424 
 0.0052481 
 0.3765281 
 136.12444 
 0.0033618 
 8.4583884 
 
 
 sigma^2 
 0.0899002 
 0.1297257 
 0.0001550 
 0.0294812 
 19.36251 
 0.0008476 
 0.2364548 
 
 
 shift number 
 2.6073349 
 1.2534005 
 0.0014973 
 0.0219005 
 3275.45712 
 0.0000000 
 5.0000000 
 
 
 N theta 
 3.6073349 
 1.2534005 
 0.0014973 
 0.0219005 
 3275.45712 
 1.0000000 
 6.0000000 
 
 
 root theta 
 1.0458358 
 0.1124104 
 0.0001343 
 0.0049341 
 519.04028 
 0.8172568 
 1.2596879 
 
 
 root beta 
 0.7494989 
 0.0372117 
 0.0000445 
 0.0017705 
 441.74762 
 0.6808556 
 0.8273908 
 
 
 all theta 
 1.0080017 
 0.6410605 
 NA 
 NA 
 NA 
 NA 
 NA 
 
 
 all beta 
 0.7891335 
 0.1974405 
 NA 
 NA 
 NA 
 NA 
 NA 
 
 
 
 
 
 Shifts 
      
 A shows the locations of the selection regimes on the phylogeny. B shows the optimum regression lines associated with each regime. C and D show the posterior distribution of  \(\beta\)  and  \(\theta\)  values associated with each regime. 
   
 Plot shows the median values for parameters associated with each selection regime, along with the posterior probability associeated with each regime.  \(\theta\)  is the intercept of the optimum regression line, and  \(\beta\)  is the coefficient of the predictor. 
 
 
 
 OU parameters 
 
 
 
  
 chain1 
 chain2 
 combined chains 
 
 
 
 
 alpha 
 3.3967852 
 3.9902225 
 3.0593154 
 
 
 sigma squared 
 0.1050424 
 0.1188536 
 0.0899002 
 
 
 stationary variance 
 0.0154620 
 0.0148931 
 0.0146929 
 
 
 phylogenetic halflife 
 0.2040598 
 0.1737114 
 0.2265694 
 
 
 phylogenetic half life in units of tree height 
 0.0027952 
 0.0023795 
 0.0031036 
 
 
 
 
 
 OU Weighted Predictor 
 
 Convergence 
   
 Plots show Gelman’s R statistics for log likelihood,  \(\alpha\) , and  \(\sigma^2\) . Solid lines indicate values Gelman’s R, and dashed lines indicate 95% CIs. 
 
 
 Likelihood 
   
 Plot shows the posterior probabilities of a shift occuring on each branch for each chain prior to merging. 
 
 
 Summary Statistics 
 
 chain1 
 
 
  
 Mean 
 SD 
 Naive SE 
 Time-series SE 
 Effective Size 
 HPD95Lower 
 HPD95Upper 
 
 
 
 
 lnL 
 34.0371561 
 16.9841906 
 0.0240072 
 0.1725718 
 9686.12060 
 23.4572121 
 45.0021240 
 
 
 prior 
 -43.0414028 
 17.3366744 
 0.0245055 
 0.5320266 
 1061.85433 
 -74.6248220 
 -13.6006896 
 
 
 alpha 
 0.0862955 
 0.4436760 
 0.0006271 
 0.0475792 
 86.95550 
 0.0000168 
 0.1818922 
 
 
 sigma^2 
 0.0031725 
 0.0132612 
 0.0000187 
 0.0030428 
 18.99399 
 0.0003987 
 0.0053736 
 
 
 shift number 
 7.7708516 
 4.0149894 
 0.0056752 
 0.1203739 
 1112.51084 
 1.0000000 
 15.0000000 
 
 
 N theta 
 8.7708516 
 4.0149894 
 0.0056752 
 0.1203739 
 1112.51084 
 2.0000000 
 16.0000000 
 
 
 root theta 
 1.0471759 
 0.1539052 
 0.0002175 
 0.0043775 
 1236.10923 
 0.7245957 
 1.3450879 
 
 
 root beta 
 0.7434921 
 0.0493741 
 0.0000698 
 0.0012742 
 1501.54666 
 0.6455499 
 0.8458869 
 
 
 all theta 
 0.9581601 
 0.8701998 
 NA 
 NA 
 NA 
 NA 
 NA 
 
 
 all beta 
 0.8192586 
 0.4168436 
 NA 
 NA 
 NA 
 NA 
 NA 
 
 
 
 
 chain2 
 
 
  
 Mean 
 SD 
 Naive SE 
 Time-series SE 
 Effective Size 
 HPD95Lower 
 HPD95Upper 
 
 
 
 
 lnL 
 36.2193168 
 5.3442745 
 0.0075542 
 0.2045992 
 682.29093 
 26.1425057 
 45.0325194 
 
 
 prior 
 -27.3900948 
 11.1956702 
 0.0158251 
 0.4123270 
 737.25312 
 -51.1514961 
 -10.1458263 
 
 
 alpha 
 4.2963449 
 6.6751439 
 0.0094354 
 0.5421482 
 151.59515 
 0.0003357 
 17.6570872 
 
 
 sigma^2 
 0.1248236 
 0.1804488 
 0.0002551 
 0.0381314 
 22.39451 
 0.0004158 
 0.5436612 
 
 
 shift number 
 3.6037271 
 2.7171061 
 0.0038406 
 0.1007678 
 727.05938 
 0.0000000 
 10.0000000 
 
 
 N theta 
 4.6037271 
 2.7171061 
 0.0038406 
 0.1007678 
 727.05938 
 1.0000000 
 11.0000000 
 
 
 root theta 
 1.0294418 
 0.1260774 
 0.0001782 
 0.0050066 
 634.13821 
 0.7858762 
 1.2785029 
 
 
 root beta 
 0.7534692 
 0.0426523 
 0.0000603 
 0.0017945 
 564.94620 
 0.6713536 
 0.8382244 
 
 
 all theta 
 0.9896406 
 0.7275289 
 NA 
 NA 
 NA 
 NA 
 NA 
 
 
 all beta 
 0.8054296 
 0.2992028 
 NA 
 NA 
 NA 
 NA 
 NA 
 
 
 
 
 combined chains 
 
 
  
 Mean 
 SD 
 Naive SE 
 Time-series SE 
 Effective Size 
 HPD95Lower 
 HPD95Upper 
 
 
 
 
 lnL 
 35.3269162 
 5.7027183 
 0.0068126 
 0.2043558 
 778.73515 
 24.5540173 
 45.3901024 
 
 
 prior 
 -36.6378793 
 16.7264913 
 0.0199819 
 0.5212416 
 1029.74934 
 -71.0768777 
 -13.5984721 
 
 
 alpha 
 2.1458487 
 5.8823090 
 0.0070272 
 0.4698360 
 156.74839 
 0.0000168 
 13.8846515 
 
 
 sigma^2 
 0.0621376 
 0.1604863 
 0.0001917 
 0.0324137 
 24.51429 
 0.0003820 
 0.4285442 
 
 
 shift number 
 6.0538573 
 4.0295435 
 0.0048138 
 0.1279281 
 992.15559 
 1.0000000 
 14.0000000 
 
 
 N theta 
 7.0538573 
 4.0295435 
 0.0048138 
 0.1279281 
 992.15559 
 2.0000000 
 15.0000000 
 
 
 root theta 
 1.0395540 
 0.1418021 
 0.0001694 
 0.0037238 
 1450.07772 
 0.7542402 
 1.3213660 
 
 
 root beta 
 0.7471385 
 0.0462549 
 0.0000553 
 0.0011885 
 1514.76778 
 0.6541054 
 0.8395345 
 
 
 all theta 
 0.9645586 
 0.8295112 
 NA 
 NA 
 NA 
 NA 
 NA 
 
 
 all beta 
 0.8174347 
 0.3907971 
 NA 
 NA 
 NA 
 NA 
 NA 
 
 
 
 
 
 Shifts 
      
 A shows the locations of the selection regimes on the phylogeny. B shows the optimum regression lines associated with each regime. C and D show the posterior distribution of  \(\beta\)  and  \(\theta\)  values associated with each regime. 
   
 Plot shows the median values for parameters associated with each selection regime, along with the posterior probability associeated with each regime.  \(\theta\)  is the intercept of the optimum regression line, and  \(\beta\)  is the coefficient of the predictor. 
 
 
 
 OU parameters 
 
 
 
  
 chain1 
 chain2 
 combined chains 
 
 
 
 
 alpha 
 0.0862955 
 4.2963449 
 2.1458487 
 
 
 sigma squared 
 0.0031725 
 0.1248236 
 0.0621376 
 
 
 stationary variance 
 0.0183817 
 0.0145267 
 0.0144786 
 
 
 phylogenetic halflife 
 8.0322527 
 0.1613342 
 0.3230177 
 
 
 phylogenetic half life in units of tree height 
 0.1100263 
 0.0022100 
 0.0044247 
 
 
 
 
 
 BM 
 
 Convergence 
   
 Plots show Gelman’s R statistics for log likelihood,  \(\alpha\) , and  \(\sigma^2\) . Solid lines indicate values Gelman’s R, and dashed lines indicate 95% CIs. 
 
 
 Likelihood 
   
 Plot shows the posterior probabilities of a shift occuring on each branch for each chain prior to merging. 
 
 
 Summary Statistics 
 
 chain1 
 
 
  
 Mean 
 SD 
 Naive SE 
 Time-series SE 
 Effective Size 
 HPD95Lower 
 HPD95Upper 
 
 
 
 
 lnL 
 26.5485381 
 5.0509005 
 0.0071395 
 0.2360795 
 457.7421 
 24.1094743 
 28.3620457 
 
 
 prior 
 0.6108970 
 0.1417179 
 0.0002003 
 0.0051922 
 744.9891 
 0.4426606 
 0.7061943 
 
 
 alpha 
 0.0000010 
 0.0000000 
 0.0000000 
 0.0000000 
 0.0000 
 0.0000010 
 0.0000010 
 
 
 sigma^2 
 0.0017322 
 0.0289745 
 0.0000410 
 0.0006944 
 1741.0983 
 0.0006316 
 0.0014304 
 
 
 shift number 
 0.0000000 
 0.0000000 
 0.0000000 
 0.0000000 
 0.0000 
 0.0000000 
 0.0000000 
 
 
 N theta 
 1.0000000 
 0.0000000 
 0.0000000 
 0.0000000 
 0.0000 
 1.0000000 
 1.0000000 
 
 
 root theta 
 1.2015515 
 0.1875839 
 0.0002652 
 0.0084088 
 497.6499 
 0.8415834 
 1.5800614 
 
 
 root beta 
 0.6993194 
 0.0499740 
 0.0000706 
 0.0015356 
 1059.1323 
 0.6016079 
 0.8002787 
 
 
 all theta 
 1.2015515 
 0.1875839 
 NA 
 NA 
 NA 
 NA 
 NA 
 
 
 all beta 
 0.6993194 
 0.0499740 
 NA 
 NA 
 NA 
 NA 
 NA 
 
 
 
 
 chain2 
 
 
  
 Mean 
 SD 
 Naive SE 
 Time-series SE 
 Effective Size 
 HPD95Lower 
 HPD95Upper 
 
 
 
 
 lnL 
 26.5629428 
 5.4926810 
 0.0077639 
 0.2882235 
 363.1700 
 24.2528337 
 28.3631810 
 
 
 prior 
 0.6183126 
 0.1445437 
 0.0002043 
 0.0057300 
 636.3396 
 0.4722565 
 0.7062234 
 
 
 alpha 
 0.0000010 
 0.0000000 
 0.0000000 
 0.0000000 
 0.0000 
 0.0000010 
 0.0000010 
 
 
 sigma^2 
 0.0018346 
 0.0293708 
 0.0000415 
 0.0007758 
 1433.3601 
 0.0006274 
 0.0014132 
 
 
 shift number 
 0.0000000 
 0.0000000 
 0.0000000 
 0.0000000 
 0.0000 
 0.0000000 
 0.0000000 
 
 
 N theta 
 1.0000000 
 0.0000000 
 0.0000000 
 0.0000000 
 0.0000 
 1.0000000 
 1.0000000 
 
 
 root theta 
 1.1845039 
 0.1806239 
 0.0002553 
 0.0076412 
 558.7645 
 0.8240929 
 1.5464148 
 
 
 root beta 
 0.7028933 
 0.0484279 
 0.0000685 
 0.0014403 
 1130.4657 
 0.6066532 
 0.7999328 
 
 
 all theta 
 1.1845039 
 0.1806239 
 NA 
 NA 
 NA 
 NA 
 NA 
 
 
 all beta 
 0.7028933 
 0.0484279 
 NA 
 NA 
 NA 
 NA 
 NA 
 
 
 
 
 combined chains 
 
 
  
 Mean 
 SD 
 Naive SE 
 Time-series SE 
 Effective Size 
 HPD95Lower 
 HPD95Upper 
 
 
 
 
 lnL 
 26.7706289 
 1.2912353 
 0.0015425 
 0.0201380 
 4111.2956 
 24.2360618 
 28.3620457 
 
 
 prior 
 0.6170407 
 0.0808391 
 0.0000966 
 0.0031029 
 678.7535 
 0.4545461 
 0.7062101 
 
 
 alpha 
 0.0000010 
 0.0000000 
 0.0000000 
 0.0000000 
 0.0000 
 0.0000010 
 0.0000010 
 
 
 sigma^2 
 0.0009983 
 0.0002071 
 0.0000002 
 0.0000036 
 3376.4739 
 0.0006390 
 0.0014160 
 
 
 shift number 
 0.0000000 
 0.0000000 
 0.0000000 
 0.0000000 
 0.0000 
 0.0000000 
 0.0000000 
 
 
 N theta 
 1.0000000 
 0.0000000 
 0.0000000 
 0.0000000 
 0.0000 
 1.0000000 
 1.0000000 
 
 
 root theta 
 1.1954987 
 0.1863705 
 0.0002226 
 0.0068648 
 737.0504 
 0.8428894 
 1.5792490 
 
 
 root beta 
 0.7006512 
 0.0495059 
 0.0000591 
 0.0012905 
 1471.7183 
 0.6033083 
 0.8003800 
 
 
 all theta 
 1.1954987 
 0.1863705 
 NA 
 NA 
 NA 
 NA 
 NA 
 
 
 all beta 
 0.7006512 
 0.0495059 
 NA 
 NA 
 NA 
 NA 
 NA 
 
 
 
 
 
 Shifts 
No shifts were detected with a posterior probability above the cutoff.
 
 OU parameters 
 
 
 
  
 chain1 
 chain2 
 combined chains 
 
 
 
 
 alpha 
 1.000000e-06 
 1.000000e-06 
 1.000000e-06 
 
 
 sigma squared 
 1.732200e-03 
 1.834600e-03 
 9.983000e-04 
 
 
 stationary variance 
 8.661245e+02 
 9.172910e+02 
 4.991699e+02 
 
 
 phylogenetic halflife 
 6.931472e+05 
 6.931472e+05 
 6.931472e+05 
 
 
 phylogenetic half life in units of tree height 
 9.494774e+03 
 9.494774e+03 
 9.494774e+03 
 
 
 
 
 
 Model Selection 
 
 Bayes Factors 
 
 
  
 OU un-weighted predictor 
 OU weighted predictor 
 BM 
 
 
 
 
 OU un-weighted predictor 
 0.00000 
 10.83171 
 22.78693 
 
 
 OU weighted predictor 
 -10.83171 
 0.00000 
 11.95522 
 
 
 BM 
 -22.78693 
 -11.95522 
 0.00000 
 
 
 
 
 
 
 Cerebellum ~ Rest of Brain 
 
 OU Un-weighted Predictor 
 
 Convergence 
   
 Plots show Gelman’s R statistics for log likelihood,  \(\alpha\) , and  \(\sigma^2\) . Solid lines indicate values Gelman’s R, and dashed lines indicate 95% CIs. 
 
 
 Likelihood 
   
 Plot shows the posterior probabilities of a shift occuring on each branch for each chain prior to merging. 
 
 
 Summary Statistics 
 
 chain1 
 
 
  
 Mean 
 SD 
 Naive SE 
 Time-series SE 
 Effective Size 
 HPD95Lower 
 HPD95Upper 
 
 
 
 
 lnL 
 49.3269425 
 3.9357571 
 0.0055632 
 0.1012674 
 1510.4870 
 44.7023903 
 54.3961533 
 
 
 prior 
 -20.9461595 
 4.7152885 
 0.0066651 
 0.1177747 
 1602.9213 
 -29.6111772 
 -13.8759109 
 
 
 alpha 
 6.8740976 
 11.0784825 
 0.0156595 
 0.9947652 
 124.0279 
 0.2779672 
 25.3686391 
 
 
 sigma^2 
 0.1275789 
 0.2041039 
 0.0002885 
 0.0335094 
 37.0996 
 0.0070807 
 0.4817957 
 
 
 shift number 
 1.8596786 
 0.9390306 
 0.0013273 
 0.0162547 
 3337.3617 
 1.0000000 
 4.0000000 
 
 
 N theta 
 2.8596786 
 0.9390306 
 0.0013273 
 0.0162547 
 3337.3617 
 2.0000000 
 5.0000000 
 
 
 root theta 
 -0.8119928 
 0.1345697 
 0.0001902 
 0.0081744 
 271.0109 
 -1.0893914 
 -0.5548172 
 
 
 root beta 
 1.1142681 
 0.0367249 
 0.0000519 
 0.0017818 
 424.8286 
 1.0456704 
 1.1869901 
 
 
 all theta 
 -0.8537486 
 0.5772799 
 NA 
 NA 
 NA 
 NA 
 NA 
 
 
 all beta 
 1.1533021 
 0.1446264 
 NA 
 NA 
 NA 
 NA 
 NA 
 
 
 
 
 chain2 
 
 
  
 Mean 
 SD 
 Naive SE 
 Time-series SE 
 Effective Size 
 HPD95Lower 
 HPD95Upper 
 
 
 
 
 lnL 
 49.2069385 
 24.8321423 
 0.0351004 
 0.2001908 
 15386.51547 
 44.7336228 
 54.3495102 
 
 
 prior 
 -20.6981186 
 4.5007928 
 0.0063619 
 0.0991912 
 2058.88521 
 -28.9382894 
 -14.0955850 
 
 
 alpha 
 5.7780187 
 9.4741714 
 0.0133918 
 0.8217482 
 132.92448 
 0.4593220 
 14.1225901 
 
 
 sigma^2 
 0.1065654 
 0.1706065 
 0.0002412 
 0.0373301 
 20.88682 
 0.0107418 
 0.2464464 
 
 
 shift number 
 1.8447236 
 0.9458469 
 0.0013370 
 0.0161186 
 3443.38114 
 1.0000000 
 4.0000000 
 
 
 N theta 
 2.8447236 
 0.9458469 
 0.0013370 
 0.0161186 
 3443.38114 
 2.0000000 
 5.0000000 
 
 
 root theta 
 -0.8175726 
 0.1336386 
 0.0001889 
 0.0084307 
 251.26502 
 -1.0821132 
 -0.5492164 
 
 
 root beta 
 1.1157420 
 0.0354847 
 0.0000502 
 0.0021581 
 270.34832 
 1.0438563 
 1.1854522 
 
 
 all theta 
 -0.8297152 
 0.5830575 
 NA 
 NA 
 NA 
 NA 
 NA 
 
 
 all beta 
 1.1484814 
 0.1445109 
 NA 
 NA 
 NA 
 NA 
 NA 
 
 
 
 
 combined chains 
 
 
  
 Mean 
 SD 
 Naive SE 
 Time-series SE 
 Effective Size 
 HPD95Lower 
 HPD95Upper 
 
 
 
 
 lnL 
 49.4082883 
 2.3190157 
 0.0027704 
 0.0330285 
 4929.79499 
 44.8529090 
 54.3869963 
 
 
 prior 
 -20.2735808 
 4.0849296 
 0.0048800 
 0.0728433 
 3144.77495 
 -27.9431388 
 -13.9333419 
 
 
 alpha 
 4.3403781 
 4.0391746 
 0.0048253 
 0.2440361 
 273.95365 
 0.3622365 
 11.2181190 
 
 
 sigma^2 
 0.0795458 
 0.0712826 
 0.0000852 
 0.0107704 
 43.80264 
 0.0077395 
 0.1954588 
 
 
 shift number 
 1.8437600 
 0.9015113 
 0.0010770 
 0.0128477 
 4923.73617 
 1.0000000 
 4.0000000 
 
 
 N theta 
 2.8437600 
 0.9015113 
 0.0010770 
 0.0128477 
 4923.73617 
 2.0000000 
 5.0000000 
 
 
 root theta 
 -0.8121158 
 0.1336923 
 0.0001597 
 0.0071305 
 351.53857 
 -1.0924216 
 -0.5548172 
 
 
 root beta 
 1.1143510 
 0.0354722 
 0.0000424 
 0.0018812 
 355.53859 
 1.0457706 
 1.1888983 
 
 
 all theta 
 -0.8479819 
 0.5802528 
 NA 
 NA 
 NA 
 NA 
 NA 
 
 
 all beta 
 1.1524684 
 0.1437857 
 NA 
 NA 
 NA 
 NA 
 NA 
 
 
 
 
 
 Shifts 
      
 A shows the locations of the selection regimes on the phylogeny. B shows the optimum regression lines associated with each regime. C and D show the posterior distribution of  \(\beta\)  and  \(\theta\)  values associated with each regime. 
   
 Plot shows the median values for parameters associated with each selection regime, along with the posterior probability associeated with each regime.  \(\theta\)  is the intercept of the optimum regression line, and  \(\beta\)  is the coefficient of the predictor. 
 
 
 
 OU parameters 
 
 
 
  
 chain1 
 chain2 
 combined chains 
 
 
 
 
 alpha 
 6.8740976 
 5.7780187 
 4.3403781 
 
 
 sigma squared 
 0.1275789 
 0.1065654 
 0.0795458 
 
 
 stationary variance 
 0.0092797 
 0.0092216 
 0.0091635 
 
 
 phylogenetic halflife 
 0.1008346 
 0.1199628 
 0.1596974 
 
 
 phylogenetic half life in units of tree height 
 0.0013812 
 0.0016433 
 0.0021875 
 
 
 
 
 
 OU Weighted Predictor 
 
 Convergence 
   
 Plots show Gelman’s R statistics for log likelihood,  \(\alpha\) , and  \(\sigma^2\) . Solid lines indicate values Gelman’s R, and dashed lines indicate 95% CIs. 
 
 
 Likelihood 
   
 Plot shows the posterior probabilities of a shift occuring on each branch for each chain prior to merging. 
 
 
 Summary Statistics 
 
 chain1 
 
 
  
 Mean 
 SD 
 Naive SE 
 Time-series SE 
 Effective Size 
 HPD95Lower 
 HPD95Upper 
 
 
 
 
 lnL 
 49.3481274 
 2.7736407 
 0.0039206 
 0.0517473 
 2872.9267 
 44.9024246 
 54.2997755 
 
 
 prior 
 -19.6678169 
 4.1849779 
 0.0059155 
 0.0888331 
 2219.4042 
 -27.4896496 
 -13.7336059 
 
 
 alpha 
 3.7221887 
 5.8580373 
 0.0082804 
 0.4490953 
 170.1482 
 0.0861110 
 11.8009314 
 
 
 sigma^2 
 0.0686369 
 0.1055998 
 0.0001493 
 0.0102375 
 106.4000 
 0.0016914 
 0.2156493 
 
 
 shift number 
 1.8430573 
 0.9128674 
 0.0012903 
 0.0152115 
 3601.4190 
 1.0000000 
 4.0000000 
 
 
 N theta 
 2.8430573 
 0.9128674 
 0.0012903 
 0.0152115 
 3601.4190 
 2.0000000 
 5.0000000 
 
 
 root theta 
 -0.8220552 
 0.1245090 
 0.0001760 
 0.0076035 
 268.1453 
 -1.0632893 
 -0.5772153 
 
 
 root beta 
 1.1169222 
 0.0329857 
 0.0000466 
 0.0019887 
 275.1177 
 1.0503486 
 1.1789120 
 
 
 all theta 
 -0.8348596 
 0.5893023 
 NA 
 NA 
 NA 
 NA 
 NA 
 
 
 all beta 
 1.1494329 
 0.1432998 
 NA 
 NA 
 NA 
 NA 
 NA 
 
 
 
 
 chain2 
 
 
  
 Mean 
 SD 
 Naive SE 
 Time-series SE 
 Effective Size 
 HPD95Lower 
 HPD95Upper 
 
 
 
 
 lnL 
 49.2633328 
 11.9856559 
 0.0169418 
 0.1048105 
 13077.1885 
 44.4475506 
 54.6753513 
 
 
 prior 
 -19.5913878 
 4.1938216 
 0.0059280 
 0.0836046 
 2516.2815 
 -27.3412844 
 -13.6981301 
 
 
 alpha 
 3.0033150 
 3.1793302 
 0.0044940 
 0.1988068 
 255.7459 
 0.1220655 
 8.8839884 
 
 
 sigma^2 
 0.0554521 
 0.0580222 
 0.0000820 
 0.0047153 
 151.4141 
 0.0022603 
 0.1624690 
 
 
 shift number 
 1.8739583 
 0.9437113 
 0.0013339 
 0.0160975 
 3436.8470 
 1.0000000 
 4.0000000 
 
 
 N theta 
 2.8739583 
 0.9437113 
 0.0013339 
 0.0160975 
 3436.8470 
 2.0000000 
 5.0000000 
 
 
 root theta 
 -0.8433433 
 0.1415927 
 0.0002001 
 0.0095398 
 220.2925 
 -1.1111085 
 -0.5703628 
 
 
 root beta 
 1.1226095 
 0.0395323 
 0.0000559 
 0.0018656 
 449.0013 
 1.0502957 
 1.1927854 
 
 
 all theta 
 -0.8553063 
 0.6042763 
 NA 
 NA 
 NA 
 NA 
 NA 
 
 
 all beta 
 1.1545158 
 0.1485375 
 NA 
 NA 
 NA 
 NA 
 NA 
 
 
 
 
 combined chains 
 
 
  
 Mean 
 SD 
 Naive SE 
 Time-series SE 
 Effective Size 
 HPD95Lower 
 HPD95Upper 
 
 
 
 
 lnL 
 49.3834736 
 2.3793226 
 0.0028424 
 0.0377764 
 3967.0407 
 44.7301931 
 54.5654094 
 
 
 prior 
 -19.5954467 
 4.1241172 
 0.0049268 
 0.0724996 
 3235.8677 
 -27.3738546 
 -13.7444268 
 
 
 alpha 
 3.2850853 
 4.4575868 
 0.0053252 
 0.2784352 
 256.3016 
 0.0949506 
 9.8046308 
 
 
 sigma^2 
 0.0607616 
 0.0831734 
 0.0000994 
 0.0066471 
 156.5705 
 0.0017156 
 0.1805526 
 
 
 shift number 
 1.8560947 
 0.9205252 
 0.0010997 
 0.0132498 
 4826.7162 
 1.0000000 
 4.0000000 
 
 
 N theta 
 2.8560947 
 0.9205252 
 0.0010997 
 0.0132498 
 4826.7162 
 2.0000000 
 5.0000000 
 
 
 root theta 
 -0.8378266 
 0.1351284 
 0.0001614 
 0.0074360 
 330.2258 
 -1.0776892 
 -0.5727548 
 
 
 root beta 
 1.1210664 
 0.0361976 
 0.0000432 
 0.0020173 
 321.9858 
 1.0518719 
 1.1855103 
 
 
 all theta 
 -0.8414627 
 0.5941776 
 NA 
 NA 
 NA 
 NA 
 NA 
 
 
 all beta 
 1.1507620 
 0.1444982 
 NA 
 NA 
 NA 
 NA 
 NA 
 
 
 
 
 
 Shifts 
      
 A shows the locations of the selection regimes on the phylogeny. B shows the optimum regression lines associated with each regime. C and D show the posterior distribution of  \(\beta\)  and  \(\theta\)  values associated with each regime. 
   
 Plot shows the median values for parameters associated with each selection regime, along with the posterior probability associeated with each regime.  \(\theta\)  is the intercept of the optimum regression line, and  \(\beta\)  is the coefficient of the predictor. 
 
 
 
 OU parameters 
 
 
 
  
 chain1 
 chain2 
 combined chains 
 
 
 
 
 alpha 
 3.7221887 
 3.0033150 
 3.2850853 
 
 
 sigma squared 
 0.0686369 
 0.0554521 
 0.0607616 
 
 
 stationary variance 
 0.0092200 
 0.0092318 
 0.0092481 
 
 
 phylogenetic halflife 
 0.1862203 
 0.2307940 
 0.2109982 
 
 
 phylogenetic half life in units of tree height 
 0.0025509 
 0.0031614 
 0.0028903 
 
 
 
 
 
 BM 
 
 Convergence 
   
 Plots show Gelman’s R statistics for log likelihood,  \(\alpha\) , and  \(\sigma^2\) . Solid lines indicate values Gelman’s R, and dashed lines indicate 95% CIs. 
 
 
 Likelihood 
   
 Plot shows the posterior probabilities of a shift occuring on each branch for each chain prior to merging. 
 
 
 Summary Statistics 
 
 chain1 
 
 
  
 Mean 
 SD 
 Naive SE 
 Time-series SE 
 Effective Size 
 HPD95Lower 
 HPD95Upper 
 
 
 
 
 lnL 
 27.7751952 
 2.4020119 
 0.0033953 
 0.0459851 
 2728.4557 
 25.2802762 
 29.4104014 
 
 
 prior 
 0.3937618 
 0.2363242 
 0.0003340 
 0.0136611 
 299.2596 
 -0.0585239 
 0.7062204 
 
 
 alpha 
 0.0000010 
 0.0000000 
 0.0000000 
 0.0000000 
 0.0000 
 0.0000010 
 0.0000010 
 
 
 sigma^2 
 0.0010826 
 0.0120831 
 0.0000171 
 0.0001197 
 10198.0175 
 0.0006101 
 0.0013692 
 
 
 shift number 
 0.0000000 
 0.0000000 
 0.0000000 
 0.0000000 
 0.0000 
 0.0000000 
 0.0000000 
 
 
 N theta 
 1.0000000 
 0.0000000 
 0.0000000 
 0.0000000 
 0.0000 
 1.0000000 
 1.0000000 
 
 
 root theta 
 -0.5335550 
 0.2761243 
 0.0003903 
 0.0183661 
 226.0361 
 -1.0676802 
 0.0195057 
 
 
 root beta 
 1.0371505 
 0.0694229 
 0.0000981 
 0.0031915 
 473.1717 
 0.9005716 
 1.1748000 
 
 
 all theta 
 -0.5335550 
 0.2761243 
 NA 
 NA 
 NA 
 NA 
 NA 
 
 
 all beta 
 1.0371505 
 0.0694229 
 NA 
 NA 
 NA 
 NA 
 NA 
 
 
 
 
 chain2 
 
 
  
 Mean 
 SD 
 Naive SE 
 Time-series SE 
 Effective Size 
 HPD95Lower 
 HPD95Upper 
 
 
 
 
 lnL 
 27.7948634 
 2.2579275 
 0.0031916 
 0.0416064 
 2945.1048 
 25.2930660 
 29.4091996 
 
 
 prior 
 0.4024882 
 0.2212113 
 0.0003127 
 0.0125847 
 308.9778 
 -0.0115160 
 0.7062136 
 
 
 alpha 
 0.0000010 
 0.0000000 
 0.0000000 
 0.0000000 
 0.0000 
 0.0000010 
 0.0000010 
 
 
 sigma^2 
 0.0010673 
 0.0121688 
 0.0000172 
 0.0000996 
 14925.6437 
 0.0006119 
 0.0013760 
 
 
 shift number 
 0.0000000 
 0.0000000 
 0.0000000 
 0.0000000 
 0.0000 
 0.0000000 
 0.0000000 
 
 
 N theta 
 1.0000000 
 0.0000000 
 0.0000000 
 0.0000000 
 0.0000 
 1.0000000 
 1.0000000 
 
 
 root theta 
 -0.5423194 
 0.2701564 
 0.0003819 
 0.0170906 
 249.8696 
 -1.0794213 
 -0.0185238 
 
 
 root beta 
 1.0386770 
 0.0680850 
 0.0000962 
 0.0030871 
 486.4145 
 0.9043142 
 1.1715899 
 
 
 all theta 
 -0.5423194 
 0.2701564 
 NA 
 NA 
 NA 
 NA 
 NA 
 
 
 all beta 
 1.0386770 
 0.0680850 
 NA 
 NA 
 NA 
 NA 
 NA 
 
 
 
 
 combined chains 
 
 
  
 Mean 
 SD 
 Naive SE 
 Time-series SE 
 Effective Size 
 HPD95Lower 
 HPD95Upper 
 
 
 
 
 lnL 
 27.8347888 
 1.2748328 
 0.0015230 
 0.0145494 
 7677.4573 
 25.3257300 
 29.4090188 
 
 
 prior 
 0.4060624 
 0.2165916 
 0.0002587 
 0.0117126 
 341.9621 
 -0.0058340 
 0.7061951 
 
 
 alpha 
 0.0000010 
 0.0000000 
 0.0000000 
 0.0000000 
 0.0000 
 0.0000010 
 0.0000010 
 
 
 sigma^2 
 0.0009620 
 0.0002027 
 0.0000002 
 0.0000010 
 37254.5836 
 0.0006154 
 0.0013760 
 
 
 shift number 
 0.0000000 
 0.0000000 
 0.0000000 
 0.0000000 
 0.0000 
 0.0000000 
 0.0000000 
 
 
 N theta 
 1.0000000 
 0.0000000 
 0.0000000 
 0.0000000 
 0.0000 
 1.0000000 
 1.0000000 
 
 
 root theta 
 -0.5446117 
 0.2660055 
 0.0003178 
 0.0144844 
 337.2734 
 -1.0719802 
 -0.0308299 
 
 
 root beta 
 1.0392547 
 0.0673263 
 0.0000804 
 0.0025535 
 695.1869 
 0.9079109 
 1.1714135 
 
 
 all theta 
 -0.5446117 
 0.2660055 
 NA 
 NA 
 NA 
 NA 
 NA 
 
 
 all beta 
 1.0392547 
 0.0673263 
 NA 
 NA 
 NA 
 NA 
 NA 
 
 
 
 
 
 Shifts 
No shifts were detected with a posterior probability above the cutoff.
 
 OU parameters 
 
 
 
  
 chain1 
 chain2 
 combined chains 
 
 
 
 
 alpha 
 1.000000e-06 
 1.000000e-06 
 1.000000e-06 
 
 
 sigma squared 
 1.082600e-03 
 1.067300e-03 
 9.620000e-04 
 
 
 stationary variance 
 5.412753e+02 
 5.336342e+02 
 4.809976e+02 
 
 
 phylogenetic halflife 
 6.931472e+05 
 6.931472e+05 
 6.931472e+05 
 
 
 phylogenetic half life in units of tree height 
 9.494774e+03 
 9.494774e+03 
 9.494774e+03 
 
 
 
 
 
 Model Selection 
 
 Bayes Factors 
 
 
  
 OU un-weighted predictor 
 OU weighted predictor 
 BM 
 
 
 
 
 OU un-weighted predictor 
 0.0000000 
 -0.1973664 
 -10.65050 
 
 
 OU weighted predictor 
 0.1973664 
 0.0000000 
 -10.45313 
 
 
 BM 
 10.6504986 
 10.4531321 
 0.00000 
 
 
 
 
 
 
 Medulla ~ Body Mass 
 
 OU Un-weighted Predictor 
 
 Convergence 
   
 Plots show Gelman’s R statistics for log likelihood,  \(\alpha\) , and  \(\sigma^2\) . Solid lines indicate values Gelman’s R, and dashed lines indicate 95% CIs. 
 
 
 Likelihood 
   
 Plot shows the posterior probabilities of a shift occuring on each branch for each chain prior to merging. 
 
 
 Summary Statistics 
 
 chain1 
 
 
  
 Mean 
 SD 
 Naive SE 
 Time-series SE 
 Effective Size 
 HPD95Lower 
 HPD95Upper 
 
 
 
 
 lnL 
 41.5090395 
 3.8289470 
 0.0054122 
 0.1171954 
 1067.4259 
 35.8256584 
 49.7403512 
 
 
 prior 
 -13.2359031 
 4.2147195 
 0.0059575 
 0.0975562 
 1866.4989 
 -21.1753098 
 -7.5672757 
 
 
 alpha 
 3.3050082 
 3.6660083 
 0.0051819 
 0.2550397 
 206.6196 
 0.1363300 
 10.3375381 
 
 
 sigma^2 
 0.0480708 
 0.0487685 
 0.0000689 
 0.0043616 
 125.0206 
 0.0026400 
 0.1485004 
 
 
 shift number 
 0.9079163 
 0.9474716 
 0.0013393 
 0.0188478 
 2527.0234 
 0.0000000 
 3.0000000 
 
 
 N theta 
 1.9079163 
 0.9474716 
 0.0013393 
 0.0188478 
 2527.0234 
 1.0000000 
 4.0000000 
 
 
 root theta 
 1.0677990 
 0.0784913 
 0.0001109 
 0.0023994 
 1070.1740 
 0.9133303 
 1.2251224 
 
 
 root beta 
 0.5870589 
 0.0223279 
 0.0000316 
 0.0006535 
 1167.5238 
 0.5429637 
 0.6312144 
 
 
 all theta 
 1.0574266 
 0.4913659 
 NA 
 NA 
 NA 
 NA 
 NA 
 
 
 all beta 
 0.5829843 
 0.1672830 
 NA 
 NA 
 NA 
 NA 
 NA 
 
 
 
 
 chain2 
 
 
  
 Mean 
 SD 
 Naive SE 
 Time-series SE 
 Effective Size 
 HPD95Lower 
 HPD95Upper 
 
 
 
 
 lnL 
 41.3631024 
 266.9873493 
 0.3773881 
 0.7808967 
 116894.5314 
 36.3959447 
 51.5809923 
 
 
 prior 
 -14.2426511 
 4.7314415 
 0.0066879 
 0.1238551 
 1459.3499 
 -22.8511111 
 -7.5456825 
 
 
 alpha 
 4.6785248 
 6.8827947 
 0.0097289 
 0.5139241 
 179.3625 
 0.0000963 
 15.8941958 
 
 
 sigma^2 
 0.0683668 
 0.1023175 
 0.0001446 
 0.0100184 
 104.3035 
 0.0002943 
 0.2297625 
 
 
 shift number 
 1.0391208 
 1.0308954 
 0.0014572 
 0.0225655 
 2087.0883 
 0.0000000 
 3.0000000 
 
 
 N theta 
 2.0391208 
 1.0308954 
 0.0014572 
 0.0225655 
 2087.0883 
 1.0000000 
 4.0000000 
 
 
 root theta 
 1.0785224 
 0.0819400 
 0.0001158 
 0.0026366 
 965.8252 
 0.9241345 
 1.2383921 
 
 
 root beta 
 0.5848160 
 0.0231503 
 0.0000327 
 0.0007174 
 1041.1920 
 0.5402858 
 0.6285270 
 
 
 all theta 
 1.0486085 
 0.5214857 
 NA 
 NA 
 NA 
 NA 
 NA 
 
 
 all beta 
 0.5846585 
 0.1845006 
 NA 
 NA 
 NA 
 NA 
 NA 
 
 
 
 
 combined chains 
 
 
  
 Mean 
 SD 
 Naive SE 
 Time-series SE 
 Effective Size 
 HPD95Lower 
 HPD95Upper 
 
 
 
 
 lnL 
 41.9231253 
 3.8363852 
 0.0045831 
 0.1238482 
 959.5443 
 36.1171349 
 51.0201071 
 
 
 prior 
 -13.6856649 
 4.4820591 
 0.0053544 
 0.0951877 
 2217.1404 
 -22.0645257 
 -7.5456825 
 
 
 alpha 
 3.7860633 
 5.5320481 
 0.0066087 
 0.3588624 
 237.6381 
 0.0000963 
 12.1936336 
 
 
 sigma^2 
 0.0551960 
 0.0811289 
 0.0000969 
 0.0067506 
 144.4347 
 0.0002899 
 0.1765419 
 
 
 shift number 
 0.9848495 
 0.9948119 
 0.0011884 
 0.0179993 
 3054.7043 
 0.0000000 
 3.0000000 
 
 
 N theta 
 1.9848495 
 0.9948119 
 0.0011884 
 0.0179993 
 3054.7043 
 1.0000000 
 4.0000000 
 
 
 root theta 
 1.0745056 
 0.0792044 
 0.0000946 
 0.0020732 
 1459.4939 
 0.9194360 
 1.2309349 
 
 
 root beta 
 0.5855490 
 0.0225094 
 0.0000269 
 0.0005687 
 1566.8394 
 0.5411605 
 0.6294026 
 
 
 all theta 
 1.0552870 
 0.5152106 
 NA 
 NA 
 NA 
 NA 
 NA 
 
 
 all beta 
 0.5832427 
 0.1810461 
 NA 
 NA 
 NA 
 NA 
 NA 
 
 
 
 
 
 Shifts 
No shifts were detected with a posterior probability above the cutoff.
 
 OU parameters 
 
 
 
  
 chain1 
 chain2 
 combined chains 
 
 
 
 
 alpha 
 3.3050082 
 4.6785248 
 3.7860633 
 
 
 sigma squared 
 0.0480708 
 0.0683668 
 0.0551960 
 
 
 stationary variance 
 0.0072724 
 0.0073064 
 0.0072894 
 
 
 phylogenetic halflife 
 0.2097263 
 0.1481551 
 0.1830786 
 
 
 phylogenetic half life in units of tree height 
 0.0028728 
 0.0020294 
 0.0025078 
 
 
 
 
 
 OU Weighted Predictor 
 
 Convergence 
   
 Plots show Gelman’s R statistics for log likelihood,  \(\alpha\) , and  \(\sigma^2\) . Solid lines indicate values Gelman’s R, and dashed lines indicate 95% CIs. 
 
 
 Likelihood 
   
 Plot shows the posterior probabilities of a shift occuring on each branch for each chain prior to merging. 
 
 
 Summary Statistics 
 
 chain1 
 
 
  
 Mean 
 SD 
 Naive SE 
 Time-series SE 
 Effective Size 
 HPD95Lower 
 HPD95Upper 
 
 
 
 
 lnL 
 41.2835153 
 13.7754579 
 0.0194717 
 0.0830299 
 27525.98063 
 35.7122525 
 49.9597488 
 
 
 prior 
 -13.6388157 
 4.3165686 
 0.0061015 
 0.0992767 
 1890.52593 
 -21.5471531 
 -7.5688246 
 
 
 alpha 
 4.2604272 
 4.4098640 
 0.0062334 
 0.3547219 
 154.55194 
 0.1307626 
 13.0623479 
 
 
 sigma^2 
 0.0632336 
 0.0605777 
 0.0000856 
 0.0117015 
 26.80038 
 0.0029137 
 0.1846570 
 
 
 shift number 
 0.8863359 
 0.9398395 
 0.0013285 
 0.0173897 
 2920.92708 
 0.0000000 
 3.0000000 
 
 
 N theta 
 1.8863359 
 0.9398395 
 0.0013285 
 0.0173897 
 2920.92708 
 1.0000000 
 4.0000000 
 
 
 root theta 
 1.0628151 
 0.0790345 
 0.0001117 
 0.0024745 
 1020.15246 
 0.9071124 
 1.2185394 
 
 
 root beta 
 0.5884737 
 0.0227734 
 0.0000322 
 0.0006632 
 1179.28354 
 0.5447026 
 0.6336061 
 
 
 all theta 
 1.0306371 
 0.4868995 
 NA 
 NA 
 NA 
 NA 
 NA 
 
 
 all beta 
 0.5931910 
 0.1673254 
 NA 
 NA 
 NA 
 NA 
 NA 
 
 
 
 
 chain2 
 
 
  
 Mean 
 SD 
 Naive SE 
 Time-series SE 
 Effective Size 
 HPD95Lower 
 HPD95Upper 
 
 
 
 
 lnL 
 41.5112926 
 15.1526428 
 0.0214183 
 0.1079103 
 19717.46412 
 35.9587216 
 50.9632229 
 
 
 prior 
 -14.8072017 
 4.5998722 
 0.0065019 
 0.1174755 
 1533.19221 
 -23.2159396 
 -7.6044929 
 
 
 alpha 
 6.5957192 
 7.4787290 
 0.0105712 
 0.6444929 
 134.65402 
 0.1690026 
 21.1131089 
 
 
 sigma^2 
 0.0982441 
 0.1050929 
 0.0001485 
 0.0224355 
 21.94206 
 0.0035006 
 0.3049199 
 
 
 shift number 
 0.9375865 
 0.9927251 
 0.0014032 
 0.0205929 
 2323.92104 
 0.0000000 
 3.0000000 
 
 
 N theta 
 1.9375865 
 0.9927251 
 0.0014032 
 0.0205929 
 2323.92104 
 1.0000000 
 4.0000000 
 
 
 root theta 
 1.0684390 
 0.0821825 
 0.0001162 
 0.0025050 
 1076.35167 
 0.9122302 
 1.2245628 
 
 
 root beta 
 0.5873688 
 0.0235899 
 0.0000333 
 0.0006783 
 1209.47255 
 0.5432975 
 0.6302896 
 
 
 all theta 
 1.0416593 
 0.4914309 
 NA 
 NA 
 NA 
 NA 
 NA 
 
 
 all beta 
 0.5890748 
 0.1662890 
 NA 
 NA 
 NA 
 NA 
 NA 
 
 
 
 
 combined chains 
 
 
  
 Mean 
 SD 
 Naive SE 
 Time-series SE 
 Effective Size 
 HPD95Lower 
 HPD95Upper 
 
 
 
 
 lnL 
 41.4593996 
 3.6096713 
 0.0043122 
 0.1089838 
 1097.01189 
 35.8481951 
 50.5724231 
 
 
 prior 
 -14.1893126 
 4.5347975 
 0.0054174 
 0.0990316 
 2096.85432 
 -22.5942861 
 -7.5687857 
 
 
 alpha 
 5.5380599 
 6.3335626 
 0.0075663 
 0.4609355 
 188.80603 
 0.1310891 
 17.7483074 
 
 
 sigma^2 
 0.0818695 
 0.0862560 
 0.0001030 
 0.0148852 
 33.57929 
 0.0029077 
 0.2550722 
 
 
 shift number 
 0.9019543 
 0.9517985 
 0.0011370 
 0.0160179 
 3530.83604 
 0.0000000 
 3.0000000 
 
 
 N theta 
 1.9019543 
 0.9517985 
 0.0011370 
 0.0160179 
 3530.83604 
 1.0000000 
 4.0000000 
 
 
 root theta 
 1.0661482 
 0.0799066 
 0.0000955 
 0.0021172 
 1424.37666 
 0.9119163 
 1.2294966 
 
 
 root beta 
 0.5878956 
 0.0222287 
 0.0000266 
 0.0005490 
 1639.57971 
 0.5433200 
 0.6319634 
 
 
 all theta 
 1.0401616 
 0.4894724 
 NA 
 NA 
 NA 
 NA 
 NA 
 
 
 all beta 
 0.5901547 
 0.1660488 
 NA 
 NA 
 NA 
 NA 
 NA 
 
 
 
 
 
 Shifts 
No shifts were detected with a posterior probability above the cutoff.
 
 OU parameters 
 
 
 
  
 chain1 
 chain2 
 combined chains 
 
 
 
 
 alpha 
 4.2604272 
 6.5957192 
 5.5380599 
 
 
 sigma squared 
 0.0632336 
 0.0982441 
 0.0818695 
 
 
 stationary variance 
 0.0074210 
 0.0074476 
 0.0073915 
 
 
 phylogenetic halflife 
 0.1626943 
 0.1050905 
 0.1251607 
 
 
 phylogenetic half life in units of tree height 
 0.0022286 
 0.0014395 
 0.0017145 
 
 
 
 
 
 BM 
 
 Convergence 
   
 Plots show Gelman’s R statistics for log likelihood,  \(\alpha\) , and  \(\sigma^2\) . Solid lines indicate values Gelman’s R, and dashed lines indicate 95% CIs. 
 
 
 Likelihood 
   
 Plot shows the posterior probabilities of a shift occuring on each branch for each chain prior to merging. 
 
 
 Summary Statistics 
 
 chain1 
 
 
  
 Mean 
 SD 
 Naive SE 
 Time-series SE 
 Effective Size 
 HPD95Lower 
 HPD95Upper 
 
 
 
 
 lnL 
 39.2152166 
 5.3639924 
 0.0075820 
 0.3038042 
 311.7373 
 36.7448552 
 41.1466455 
 
 
 prior 
 0.6788686 
 0.1204073 
 0.0001702 
 0.0043025 
 783.1856 
 0.6330636 
 0.7062662 
 
 
 alpha 
 0.0000010 
 0.0000000 
 0.0000000 
 0.0000000 
 0.0000 
 0.0000010 
 0.0000010 
 
 
 sigma^2 
 0.0011216 
 0.0287079 
 0.0000406 
 0.0007992 
 1290.1996 
 0.0001907 
 0.0004964 
 
 
 shift number 
 0.0000000 
 0.0000000 
 0.0000000 
 0.0000000 
 0.0000 
 0.0000000 
 0.0000000 
 
 
 N theta 
 1.0000000 
 0.0000000 
 0.0000000 
 0.0000000 
 0.0000 
 1.0000000 
 1.0000000 
 
 
 root theta 
 1.1952786 
 0.1189474 
 0.0001681 
 0.0033782 
 1239.7808 
 0.9562364 
 1.4226816 
 
 
 root beta 
 0.5441943 
 0.0294542 
 0.0000416 
 0.0006725 
 1918.4505 
 0.4873376 
 0.6020805 
 
 
 all theta 
 1.1952786 
 0.1189474 
 NA 
 NA 
 NA 
 NA 
 NA 
 
 
 all beta 
 0.5441943 
 0.0294542 
 NA 
 NA 
 NA 
 NA 
 NA 
 
 
 
 
 chain2 
 
 
  
 Mean 
 SD 
 Naive SE 
 Time-series SE 
 Effective Size 
 HPD95Lower 
 HPD95Upper 
 
 
 
 
 lnL 
 39.2401535 
 5.2602146 
 0.0074353 
 0.2921217 
 324.2496 
 36.7917790 
 41.1481149 
 
 
 prior 
 0.6801823 
 0.1247115 
 0.0001763 
 0.0046029 
 734.0918 
 0.6372407 
 0.7062661 
 
 
 alpha 
 0.0000010 
 0.0000000 
 0.0000000 
 0.0000000 
 0.0000 
 0.0000010 
 0.0000010 
 
 
 sigma^2 
 0.0011499 
 0.0297980 
 0.0000421 
 0.0008503 
 1227.9455 
 0.0001909 
 0.0005005 
 
 
 shift number 
 0.0000000 
 0.0000000 
 0.0000000 
 0.0000000 
 0.0000 
 0.0000000 
 0.0000000 
 
 
 N theta 
 1.0000000 
 0.0000000 
 0.0000000 
 0.0000000 
 0.0000 
 1.0000000 
 1.0000000 
 
 
 root theta 
 1.1881919 
 0.1198608 
 0.0001694 
 0.0034659 
 1195.9835 
 0.9607080 
 1.4220706 
 
 
 root beta 
 0.5460381 
 0.0290105 
 0.0000410 
 0.0006493 
 1996.5603 
 0.4887957 
 0.6019384 
 
 
 all theta 
 1.1881919 
 0.1198608 
 NA 
 NA 
 NA 
 NA 
 NA 
 
 
 all beta 
 0.5460381 
 0.0290105 
 NA 
 NA 
 NA 
 NA 
 NA 
 
 
 
 
 combined chains 
 
 
  
 Mean 
 SD 
 Naive SE 
 Time-series SE 
 Effective Size 
 HPD95Lower 
 HPD95Upper 
 
 
 
 
 lnL 
 39.5398235 
 1.3237531 
 0.0015814 
 0.0206836 
 4096.012 
 36.9633492 
 41.1481359 
 
 
 prior 
 0.6843679 
 0.0222519 
 0.0000266 
 0.0004679 
 2261.199 
 0.6398485 
 0.7062674 
 
 
 alpha 
 0.0000010 
 0.0000000 
 0.0000000 
 0.0000000 
 0.000 
 0.0000010 
 0.0000010 
 
 
 sigma^2 
 0.0003298 
 0.0000817 
 0.0000001 
 0.0000019 
 1931.510 
 0.0001955 
 0.0004949 
 
 
 shift number 
 0.0000000 
 0.0000000 
 0.0000000 
 0.0000000 
 0.000 
 0.0000000 
 0.0000000 
 
 
 N theta 
 1.0000000 
 0.0000000 
 0.0000000 
 0.0000000 
 0.000 
 1.0000000 
 1.0000000 
 
 
 root theta 
 1.1910668 
 0.1129721 
 0.0001350 
 0.0025704 
 1931.692 
 0.9652457 
 1.4101063 
 
 
 root beta 
 0.5454135 
 0.0279189 
 0.0000334 
 0.0005067 
 3036.491 
 0.4910910 
 0.6011421 
 
 
 all theta 
 1.1910668 
 0.1129721 
 NA 
 NA 
 NA 
 NA 
 NA 
 
 
 all beta 
 0.5454135 
 0.0279189 
 NA 
 NA 
 NA 
 NA 
 NA 
 
 
 
 
 
 Shifts 
No shifts were detected with a posterior probability above the cutoff.
 
 OU parameters 
 
 
 
  
 chain1 
 chain2 
 combined chains 
 
 
 
 
 alpha 
 1.000000e-06 
 1.000000e-06 
 1.000000e-06 
 
 
 sigma squared 
 1.121600e-03 
 1.149900e-03 
 3.298000e-04 
 
 
 stationary variance 
 5.608190e+02 
 5.749726e+02 
 1.648884e+02 
 
 
 phylogenetic halflife 
 6.931472e+05 
 6.931472e+05 
 6.931472e+05 
 
 
 phylogenetic half life in units of tree height 
 9.494774e+03 
 9.494774e+03 
 9.494774e+03 
 
 
 
 
 
 Model Selection 
 
 Bayes Factors 
 
 
  
 OU un-weighted predictor 
 OU weighted predictor 
 BM 
 
 
 
 
 OU un-weighted predictor 
 0.000000 
 2.781395 
 10.203771 
 
 
 OU weighted predictor 
 -2.781395 
 0.000000 
 7.422375 
 
 
 BM 
 -10.203771 
 -7.422375 
 0.000000 
 
 
 
 
 
 
 Medulla ~ Brain - Medulla 
 
 OU Un-weighted Predictor 
 
 Convergence 
   
 Plots show Gelman’s R statistics for log likelihood,  \(\alpha\) , and  \(\sigma^2\) . Solid lines indicate values Gelman’s R, and dashed lines indicate 95% CIs. 
 
 
 Likelihood 
   
 Plot shows the posterior probabilities of a shift occuring on each branch for each chain prior to merging. 
 
 
 Summary Statistics 
 
 chain1 
 
 
  
 Mean 
 SD 
 Naive SE 
 Time-series SE 
 Effective Size 
 HPD95Lower 
 HPD95Upper 
 
 
 
 
 lnL 
 1.7522090 
 3.7995359 
 0.0053707 
 0.0776254 
 2395.81636 
 -4.5470349 
 10.0104748 
 
 
 prior 
 -22.4496164 
 7.5935414 
 0.0107335 
 0.1237780 
 3763.58437 
 -35.9808618 
 -7.5510880 
 
 
 alpha 
 1.8312202 
 5.4169974 
 0.0076570 
 0.3958960 
 187.22117 
 0.0018216 
 5.2534384 
 
 
 sigma^2 
 0.1965629 
 0.5648455 
 0.0007984 
 0.0604427 
 87.33156 
 0.0016171 
 0.5731199 
 
 
 shift number 
 3.2620115 
 1.8079498 
 0.0025555 
 0.0278850 
 4203.70457 
 0.0000000 
 6.0000000 
 
 
 N theta 
 4.2620115 
 1.8079498 
 0.0025555 
 0.0278850 
 4203.70457 
 1.0000000 
 7.0000000 
 
 
 root theta 
 0.0386984 
 0.1469366 
 0.0002077 
 0.0049100 
 895.55421 
 -0.2463257 
 0.3293682 
 
 
 root beta 
 2.0211784 
 0.0989513 
 0.0001399 
 0.0033985 
 847.73257 
 1.8246235 
 2.2197310 
 
 
 all theta 
 -0.0000263 
 0.5695715 
 NA 
 NA 
 NA 
 NA 
 NA 
 
 
 all beta 
 2.0143173 
 0.3559705 
 NA 
 NA 
 NA 
 NA 
 NA 
 
 
 
 
 chain2 
 
 
  
 Mean 
 SD 
 Naive SE 
 Time-series SE 
 Effective Size 
 HPD95Lower 
 HPD95Upper 
 
 
 
 
 lnL 
 1.6152515 
 4.0382400 
 0.0057081 
 0.0762111 
 2807.6801 
 -4.7612341 
 9.9148239 
 
 
 prior 
 -22.3536421 
 7.3950545 
 0.0104530 
 0.1184226 
 3899.5440 
 -35.5151023 
 -7.5568869 
 
 
 alpha 
 1.6134493 
 2.4163646 
 0.0034155 
 0.1557324 
 240.7505 
 0.0077718 
 4.9349811 
 
 
 sigma^2 
 0.1748316 
 0.2657741 
 0.0003757 
 0.0228881 
 134.8354 
 0.0027762 
 0.5349240 
 
 
 shift number 
 3.1977419 
 1.7763906 
 0.0025109 
 0.0261610 
 4610.7304 
 0.0000000 
 6.0000000 
 
 
 N theta 
 4.1977419 
 1.7763906 
 0.0025109 
 0.0261610 
 4610.7304 
 1.0000000 
 7.0000000 
 
 
 root theta 
 0.0328168 
 0.1477508 
 0.0002088 
 0.0050882 
 843.2126 
 -0.2506327 
 0.3332717 
 
 
 root beta 
 2.0255728 
 0.0978225 
 0.0001383 
 0.0034046 
 825.5666 
 1.8298877 
 2.2177652 
 
 
 all theta 
 -0.0096880 
 0.5575420 
 NA 
 NA 
 NA 
 NA 
 NA 
 
 
 all beta 
 2.0231221 
 0.3577688 
 NA 
 NA 
 NA 
 NA 
 NA 
 
 
 
 
 combined chains 
 
 
  
 Mean 
 SD 
 Naive SE 
 Time-series SE 
 Effective Size 
 HPD95Lower 
 HPD95Upper 
 
 
 
 
 lnL 
 1.7491591 
 3.8474998 
 0.0045963 
 0.0681550 
 3186.8492 
 -4.7366259 
 10.1403367 
 
 
 prior 
 -22.3965279 
 7.5550059 
 0.0090254 
 0.1058632 
 5093.0693 
 -35.8767328 
 -7.5518260 
 
 
 alpha 
 1.8112893 
 4.8132570 
 0.0057501 
 0.3052256 
 248.6772 
 0.0016737 
 5.1942068 
 
 
 sigma^2 
 0.1947053 
 0.5049689 
 0.0006033 
 0.0434987 
 134.7647 
 0.0015708 
 0.5593491 
 
 
 shift number 
 3.2251050 
 1.8005597 
 0.0021510 
 0.0236563 
 5793.2264 
 0.0000000 
 6.0000000 
 
 
 N theta 
 4.2251050 
 1.8005597 
 0.0021510 
 0.0236563 
 5793.2264 
 1.0000000 
 7.0000000 
 
 
 root theta 
 0.0379746 
 0.1478402 
 0.0001766 
 0.0043464 
 1156.9536 
 -0.2504921 
 0.3326066 
 
 
 root beta 
 2.0219536 
 0.0985401 
 0.0001177 
 0.0029649 
 1104.5665 
 1.8254760 
 2.2181488 
 
 
 all theta 
 -0.0018032 
 0.5640189 
 NA 
 NA 
 NA 
 NA 
 NA 
 
 
 all beta 
 2.0164429 
 0.3569081 
 NA 
 NA 
 NA 
 NA 
 NA 
 
 
 
 
 
 Shifts 
No shifts were detected with a posterior probability above the cutoff.
 
 OU parameters 
 
 
 
  
 chain1 
 chain2 
 combined chains 
 
 
 
 
 alpha 
 1.8312202 
 1.6134493 
 1.8112893 
 
 
 sigma squared 
 0.1965629 
 0.1748316 
 0.1947053 
 
 
 stationary variance 
 0.0536699 
 0.0541795 
 0.0537477 
 
 
 phylogenetic halflife 
 0.3785166 
 0.4296058 
 0.3826816 
 
 
 phylogenetic half life in units of tree height 
 0.0051849 
 0.0058848 
 0.0052420 
 
 
 
 
 
 OU Weighted Predictor 
 
 Convergence 
   
 Plots show Gelman’s R statistics for log likelihood,  \(\alpha\) , and  \(\sigma^2\) . Solid lines indicate values Gelman’s R, and dashed lines indicate 95% CIs. 
 
 
 Likelihood 
   
 Plot shows the posterior probabilities of a shift occuring on each branch for each chain prior to merging. 
 
 
 Summary Statistics 
 
 chain1 
 
 
  
 Mean 
 SD 
 Naive SE 
 Time-series SE 
 Effective Size 
 HPD95Lower 
 HPD95Upper 
 
 
 
 
 lnL 
 1.6870223 
 6.4239754 
 0.0090803 
 0.0806102 
 6350.79032 
 -4.5209183 
 9.7571172 
 
 
 prior 
 -22.8214888 
 8.0430091 
 0.0113688 
 0.1381083 
 3391.54429 
 -37.1071472 
 -7.5475393 
 
 
 alpha 
 0.8953529 
 0.7386092 
 0.0010440 
 0.0476398 
 240.37498 
 0.0050705 
 2.2895193 
 
 
 sigma^2 
 0.0937568 
 0.0717741 
 0.0001015 
 0.0233151 
 9.47674 
 0.0024696 
 0.2249473 
 
 
 shift number 
 3.4919511 
 1.9689116 
 0.0027831 
 0.0335574 
 3442.50539 
 0.0000000 
 7.0000000 
 
 
 N theta 
 4.4919511 
 1.9689116 
 0.0027831 
 0.0335574 
 3442.50539 
 1.0000000 
 8.0000000 
 
 
 root theta 
 0.0248345 
 0.1556828 
 0.0002201 
 0.0053766 
 838.43474 
 -0.2825694 
 0.3339287 
 
 
 root beta 
 2.0288008 
 0.1038741 
 0.0001468 
 0.0036624 
 804.42878 
 1.8130806 
 2.2263659 
 
 
 all theta 
 -0.0130095 
 0.5930876 
 NA 
 NA 
 NA 
 NA 
 NA 
 
 
 all beta 
 2.0217922 
 0.3737887 
 NA 
 NA 
 NA 
 NA 
 NA 
 
 
 
 
 chain2 
 
 
  
 Mean 
 SD 
 Naive SE 
 Time-series SE 
 Effective Size 
 HPD95Lower 
 HPD95Upper 
 
 
 
 
 lnL 
 1.8445543 
 15.6968246 
 0.0221875 
 0.1206296 
 16932.28259 
 -4.6937270 
 10.1958533 
 
 
 prior 
 -22.7489227 
 8.3598458 
 0.0118167 
 0.1454702 
 3302.54166 
 -37.8490750 
 -7.5504050 
 
 
 alpha 
 0.2823932 
 0.2564308 
 0.0003625 
 0.0173332 
 218.86754 
 0.0029359 
 0.7942673 
 
 
 sigma^2 
 0.0289217 
 0.0233267 
 0.0000330 
 0.0080629 
 8.36999 
 0.0024580 
 0.0803564 
 
 
 shift number 
 3.7266799 
 2.0425844 
 0.0028872 
 0.0349737 
 3410.96417 
 0.0000000 
 7.0000000 
 
 
 N theta 
 4.7266799 
 2.0425844 
 0.0028872 
 0.0349737 
 3410.96417 
 1.0000000 
 8.0000000 
 
 
 root theta 
 0.0323440 
 0.1572310 
 0.0002222 
 0.0053410 
 866.63044 
 -0.2792059 
 0.3391053 
 
 
 root beta 
 2.0226985 
 0.1046363 
 0.0001479 
 0.0036066 
 841.73636 
 1.8171688 
 2.2287718 
 
 
 all theta 
 -0.0123135 
 0.6117893 
 NA 
 NA 
 NA 
 NA 
 NA 
 
 
 all beta 
 2.0166927 
 0.3750270 
 NA 
 NA 
 NA 
 NA 
 NA 
 
 
 
 
 combined chains 
 
 
  
 Mean 
 SD 
 Naive SE 
 Time-series SE 
 Effective Size 
 HPD95Lower 
 HPD95Upper 
 
 
 
 
 lnL 
 1.8177312 
 3.7712204 
 0.0045052 
 0.0766203 
 2422.56664 
 -4.6856370 
 10.0190088 
 
 
 prior 
 -22.6661914 
 8.0869967 
 0.0096610 
 0.1176754 
 4722.84043 
 -37.1673859 
 -7.5519056 
 
 
 alpha 
 0.6444566 
 0.6271871 
 0.0007493 
 0.0382279 
 269.17373 
 0.0031093 
 1.9495866 
 
 
 sigma^2 
 0.0673232 
 0.0619972 
 0.0000741 
 0.0187136 
 10.97568 
 0.0024284 
 0.2001058 
 
 
 shift number 
 3.5607389 
 1.9872417 
 0.0023740 
 0.0286543 
 4809.74818 
 0.0000000 
 7.0000000 
 
 
 N theta 
 4.5607389 
 1.9872417 
 0.0023740 
 0.0286543 
 4809.74818 
 1.0000000 
 8.0000000 
 
 
 root theta 
 0.0266911 
 0.1558989 
 0.0001862 
 0.0045077 
 1196.11490 
 -0.2792444 
 0.3398225 
 
 
 root beta 
 2.0273719 
 0.1044117 
 0.0001247 
 0.0031082 
 1128.44610 
 1.8109234 
 2.2274215 
 
 
 all theta 
 -0.0158730 
 0.5957897 
 NA 
 NA 
 NA 
 NA 
 NA 
 
 
 all beta 
 2.0223107 
 0.3721234 
 NA 
 NA 
 NA 
 NA 
 NA 
 
 
 
 
 
 Shifts 
No shifts were detected with a posterior probability above the cutoff.
 
 OU parameters 
 
 
 
  
 chain1 
 chain2 
 combined chains 
 
 
 
 
 alpha 
 0.8953529 
 0.2823932 
 0.6444566 
 
 
 sigma squared 
 0.0937568 
 0.0289217 
 0.0673232 
 
 
 stationary variance 
 0.0523574 
 0.0512082 
 0.0522326 
 
 
 phylogenetic halflife 
 0.7741609 
 2.4545467 
 1.0755530 
 
 
 phylogenetic half life in units of tree height 
 0.0106045 
 0.0336225 
 0.0147330 
 
 
 
 
 
 BM 
 
 Convergence 
   
 Plots show Gelman’s R statistics for log likelihood,  \(\alpha\) , and  \(\sigma^2\) . Solid lines indicate values Gelman’s R, and dashed lines indicate 95% CIs. 
 
 
 Likelihood 
   
 Plot shows the posterior probabilities of a shift occuring on each branch for each chain prior to merging. 
 
 
 Summary Statistics 
 
 chain1 
 
 
  
 Mean 
 SD 
 Naive SE 
 Time-series SE 
 Effective Size 
 HPD95Lower 
 HPD95Upper 
 
 
 
 
 lnL 
 -3.1043191 
 4.9844194 
 0.0070455 
 0.3717504 
 179.77376 
 -5.6082686 
 -0.7790303 
 
 
 prior 
 0.0007215 
 0.5969700 
 0.0008438 
 0.0597636 
 99.77721 
 -1.0837300 
 0.7060184 
 
 
 alpha 
 0.0000010 
 0.0000000 
 0.0000000 
 0.0000000 
 0.00000 
 0.0000010 
 0.0000010 
 
 
 sigma^2 
 0.0046895 
 0.0451218 
 0.0000638 
 0.0021074 
 458.45575 
 0.0016114 
 0.0040762 
 
 
 shift number 
 0.0000000 
 0.0000000 
 0.0000000 
 0.0000000 
 0.00000 
 0.0000000 
 0.0000000 
 
 
 N theta 
 1.0000000 
 0.0000000 
 0.0000000 
 0.0000000 
 0.00000 
 1.0000000 
 1.0000000 
 
 
 root theta 
 0.5914128 
 0.3553966 
 0.0005024 
 0.0282264 
 158.53195 
 -0.1616797 
 1.2283366 
 
 
 root beta 
 1.6128707 
 0.2279454 
 0.0003222 
 0.0230127 
 98.11330 
 1.2001830 
 2.0837462 
 
 
 all theta 
 0.5914128 
 0.3553966 
 NA 
 NA 
 NA 
 NA 
 NA 
 
 
 all beta 
 1.6128707 
 0.2279454 
 NA 
 NA 
 NA 
 NA 
 NA 
 
 
 
 
 chain2 
 
 
  
 Mean 
 SD 
 Naive SE 
 Time-series SE 
 Effective Size 
 HPD95Lower 
 HPD95Upper 
 
 
 
 
 lnL 
 -3.2260003 
 5.0025544 
 0.0070711 
 0.3792135 
 174.02681 
 -6.1554430 
 -0.7650817 
 
 
 prior 
 -0.1125735 
 0.7441400 
 0.0010518 
 0.0877217 
 71.96071 
 -1.6193891 
 0.7055136 
 
 
 alpha 
 0.0000010 
 0.0000000 
 0.0000000 
 0.0000000 
 0.00000 
 0.0000010 
 0.0000010 
 
 
 sigma^2 
 0.0044840 
 0.0399889 
 0.0000565 
 0.0015834 
 637.79695 
 0.0015618 
 0.0042720 
 
 
 shift number 
 0.0000000 
 0.0000000 
 0.0000000 
 0.0000000 
 0.00000 
 0.0000000 
 0.0000000 
 
 
 N theta 
 1.0000000 
 0.0000000 
 0.0000000 
 0.0000000 
 0.00000 
 1.0000000 
 1.0000000 
 
 
 root theta 
 0.6250258 
 0.4270128 
 0.0006036 
 0.0403877 
 111.78477 
 -0.2627850 
 1.4495799 
 
 
 root beta 
 1.5974439 
 0.2674953 
 0.0003781 
 0.0315393 
 71.93302 
 1.0813234 
 2.1265179 
 
 
 all theta 
 0.6250258 
 0.4270128 
 NA 
 NA 
 NA 
 NA 
 NA 
 
 
 all beta 
 1.5974439 
 0.2674953 
 NA 
 NA 
 NA 
 NA 
 NA 
 
 
 
 
 combined chains 
 
 
  
 Mean 
 SD 
 Naive SE 
 Time-series SE 
 Effective Size 
 HPD95Lower 
 HPD95Upper 
 
 
 
 
 lnL 
 -2.8217676 
 1.4453849 
 0.0017267 
 0.0513652 
 791.82508 
 -5.6473066 
 -0.7748568 
 
 
 prior 
 -0.0812564 
 0.6907863 
 0.0008252 
 0.0710773 
 94.45503 
 -1.4578125 
 0.7059570 
 
 
 alpha 
 0.0000010 
 0.0000000 
 0.0000000 
 0.0000000 
 0.00000 
 0.0000010 
 0.0000010 
 
 
 sigma^2 
 0.0027455 
 0.0006764 
 0.0000008 
 0.0000286 
 559.47729 
 0.0015901 
 0.0040769 
 
 
 shift number 
 0.0000000 
 0.0000000 
 0.0000000 
 0.0000000 
 0.00000 
 0.0000000 
 0.0000000 
 
 
 N theta 
 1.0000000 
 0.0000000 
 0.0000000 
 0.0000000 
 0.00000 
 1.0000000 
 1.0000000 
 
 
 root theta 
 0.6301677 
 0.3939463 
 0.0004706 
 0.0292737 
 181.10074 
 -0.2056259 
 1.3646437 
 
 
 root beta 
 1.5939555 
 0.2496945 
 0.0002983 
 0.0232804 
 115.03656 
 1.1096241 
 2.1067321 
 
 
 all theta 
 0.6301677 
 0.3939463 
 NA 
 NA 
 NA 
 NA 
 NA 
 
 
 all beta 
 1.5939555 
 0.2496945 
 NA 
 NA 
 NA 
 NA 
 NA 
 
 
 
 
 
 Shifts 
No shifts were detected with a posterior probability above the cutoff.
 
 OU parameters 
 
 
 
  
 chain1 
 chain2 
 combined chains 
 
 
 
 
 alpha 
 1.000000e-06 
 1.000000e-06 
 1.000000e-06 
 
 
 sigma squared 
 4.689500e-03 
 4.484000e-03 
 2.745500e-03 
 
 
 stationary variance 
 2.344731e+03 
 2.241980e+03 
 1.372757e+03 
 
 
 phylogenetic halflife 
 6.931472e+05 
 6.931472e+05 
 6.931472e+05 
 
 
 phylogenetic half life in units of tree height 
 9.494774e+03 
 9.494774e+03 
 9.494774e+03 
 
 
 
 
 
 Model Selection 
 
 Bayes Factors 
 
 
  
 OU un-weighted predictor 
 OU weighted predictor 
 BM 
 
 
 
 
 OU un-weighted predictor 
 0.000000 
 -38.11658 
 3.868261 
 
 
 OU weighted predictor 
 38.116580 
 0.00000 
 41.984840 
 
 
 BM 
 -3.868261 
 -41.98484 
 0.000000 
 
 
 
 
 


 
 

 

 

 
 

 
 
